# The discovery of pyridinium 1,2,4-triazines with enhanced performance in bioconjugation reactions†
†Electronic supplementary information (ESI) available: Experimental procedures; NMR; LC-MS; UV-Vis and fluorescence. See DOI: 10.1039/c6sc05442k


**DOI:** 10.1039/c6sc05442k

**Published:** 2017-03-01

**Authors:** Sebastian J. Siegl, Rastislav Dzijak, Arcadio Vázquez, Radek Pohl, Milan Vrabel

**Affiliations:** a Institute of Organic Chemistry and Biochemistry of the Czech Academy of Sciences , Flemingovo nám. 2 , 16610 , Prague , Czech Republic . Email: vrabel@uochb.cas.cz

## Abstract

1,2,4-Triazines have recently been identified as versatile dienes participating in the inverse electron-demand Diels–Alder reaction with strained dienophiles. However, their widespread utility in bioconjugation reactions is still limited. Herein, we report a systematic study on the reactivity of various 1,2,4-triazines with *trans*-cyclooctenes showing that the structure of both the triazine and the dienophile significantly affect the reaction rate. Our kinetic study led to the discovery of novel cationic 1,2,4-triazines with superior properties for bioconjugation reactions. We have developed an efficient method that enables their late-stage functionalization and allows for easy access to various useful heterobifunctional scaffolds. In addition, these charged dienes form unprecedented fluorescent products upon reaction with *trans*-cyclooctenes and can be used for fluorogenic labeling of subcellular compartments in live cells.

## Abstract 2

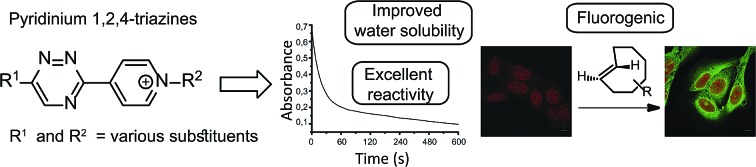
A novel class of pyridinium 1,2,4-triazines with excellent properties for use in bioconjugation reactions was discovered from a systematic kinetic study.

## Introduction

Bioorthogonal reactions have become an invaluable tool for studying biomolecules in their native environment.^1^ The success of these studies strongly depends on the inherent chemical properties of the reagents being employed in them. There has been an extensive effort to develop bioorthogonal probes which are inert to natural functional groups, are sufficiently water soluble, and which react selectively with high rates under strict biological conditions.^2^ The improved design of reagents together with systematic kinetic studies has led to substantial advancements in this regard.^3^

The inverse electron-demand Diels–Alder reaction (IEDDA) of 1,2,4,5-tetrazines with strained alkenes is the reaction of choice when high reaction rates are desirable.^4^ This particular cycloaddition has found numerous applications in biomolecule labeling, live cell labeling as well as in diagnostics.^5^ Unfortunately, the exceptional reactivity of 1,2,4,5-tetrazines is often indispensably connected to their reduced stability toward biological nucleophiles.5c,5f,^6^ 1,2,4-Triazines were recently identified as alternative heterodienes which participate in IEDDA reactions with *trans*-cyclooctene (TCO) and bicyclononyne (BCN) derivatives.^7^ Although 1,2,4-triazines show remarkable stability under biological conditions7*b* their reactivity is considerably reduced when compared to 1,2,4,5-tetrazines. This hampers their broader utility in bioconjugation reactions.

To gain deeper insight into the reactivity of 1,2,4-triazines with TCOs, we decided to systematically investigate the influence of various substituents on the kinetics of the reaction. Our data show that the reaction rates can vary by orders of magnitude with the second-order rate constants ranging from 0.007 M^–1^ s^–1^ up to more than 20 M^–1^ s^–1^ depending on the structure of both the 1,2,4-triazine and the TCO. In addition, our study led to the discovery of a novel class of cationic pyridinium 1,2,4-triazines with enhanced performance in bioconjugation reactions. The advances provided by these charged heterodienes are manifold. Firstly, they are charged, which inherently improves their water solubility. Secondly, when compared to analogous derivatives bearing an unsubstituted pyridine moiety the reactivity of the pyridinium triazines increases significantly while their stability under biological conditions remains excellent. Thirdly, their structural features in combination with the developed synthetic methodology enable further derivatization of the scaffold and preparation of various useful heterobifunctional probes. The last advance presented herein is the formation of unprecedented fluorescent products upon reaction of the pyridinium 1,2,4-triazines with TCOs. This chemistry can be used for bioimaging as we demonstrate by fluorogenic cell labeling experiments.

## Results and discussion

**Scheme 1:**
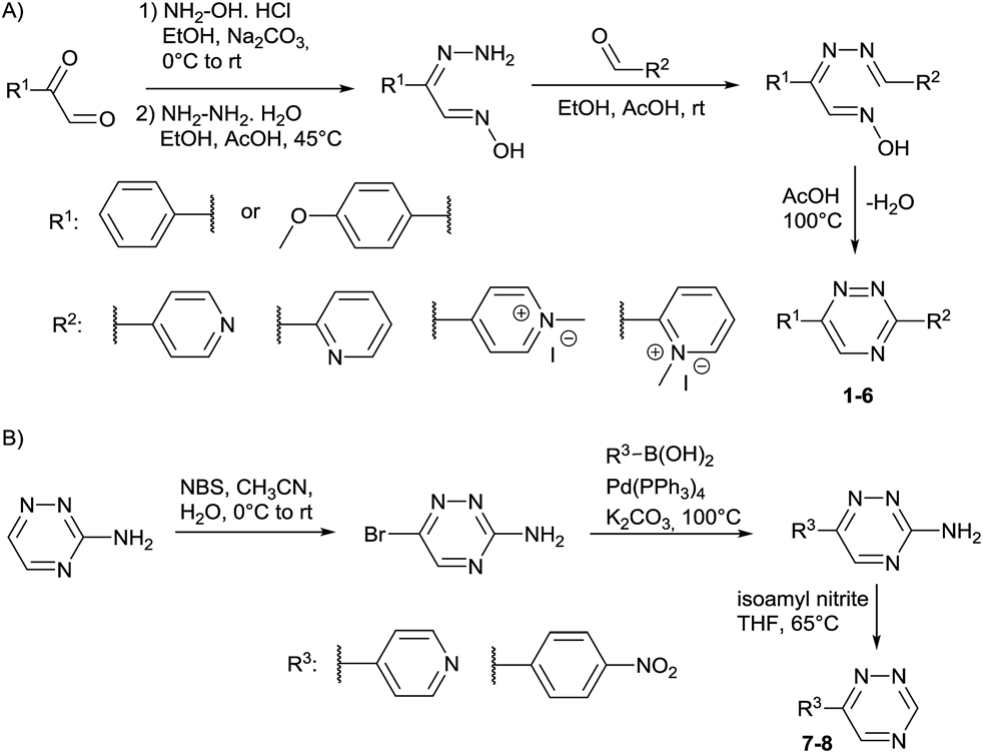
Synthesis of mono- and bis-substituted 1,2,4-triazines.

Our study began with the synthesis of various 1,2,4-triazines. We first prepared 3,6-bisaryl substituted 1,2,4-triazines starting from the respective glyoxal derivatives in a sequence of reactions depicted in Scheme 1A.^8^ It is worth mentioning that the success of the last dehydration–cyclization step strongly depends on the carboxaldehyde used. While 2- and 4-pyridyl derivatives led to the formation of the desired cyclized products, for example simple benzaldehyde did not.8*b* On the other hand, reaction with 1-methylpyridinium 2- or 4-carboxaldehydes proceeded smoothly yielding a new type of charged pyridinium 1,2,4-triazines. For comparison, we also prepared monosubstituted 6-aryl 1,2,4-triazines starting from commercially available 3-amino 1,2,4-triazine utilizing a cross coupling–deamination reaction sequence reported previously (Scheme 1B).7*b*

To study the reactivity of 1,2,4-triazines in the IEDDA reaction we next synthesized a series of TCOs using the photochemical protocol developed by Fox and coworkers.^9^ We determined the second-order rate constants in CH_3_CN/H_2_O (1/1) at room temperature under pseudo first-order conditions using an excess of the corresponding TCO. Since the reactivity of various triazines as well as of TCOs differed significantly, we followed the progress of the reaction either by HPLC, for slower derivatives, or by UV/Vis spectroscopy, for faster derivatives (for details see ESI†). All experiments were performed in triplicate and the results are summarized in Table 1.

**Table 1:** Second-order rate constants (in M^–1^ s^–1^ × 10^–2^) of the reaction between 1,2,4-triazines and TCOs^*a*^

	Triazine/TCO	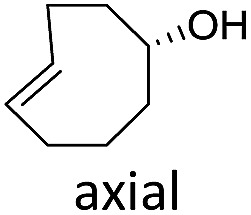	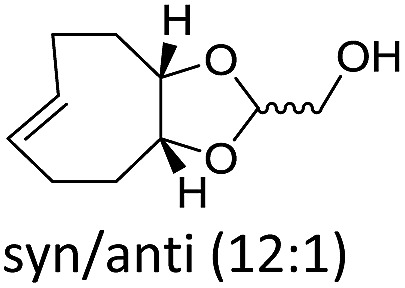	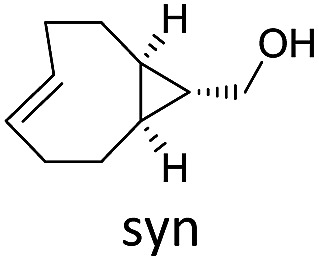
**1**	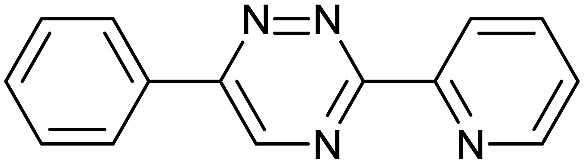	2.5 ± 0.1	56 ± 0.8	190 ± 3.0
**2**	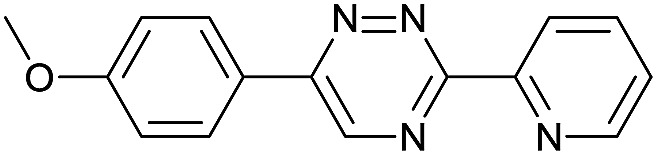	2.1 ± 0.2	36 ± 0.3	120 ± 1.0
**3**	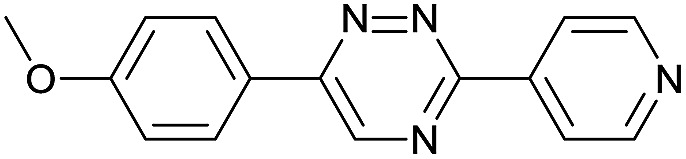	1.7 ± 0.4	35 ± 2.0	140 ± 3.0
**4**	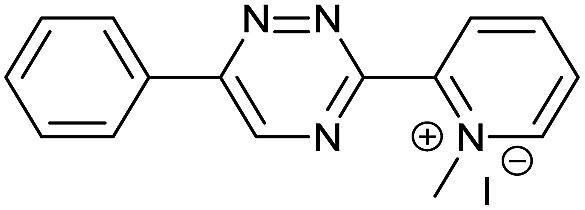	0.7 ± 0.1	8.3 ± 1.3	30 ± 1.0
**5**	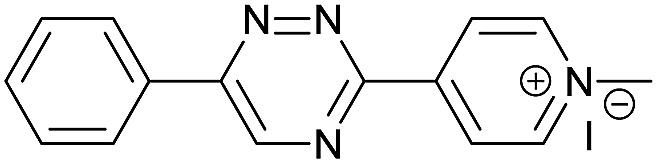	19.3 ± 0.2	260 ± 4.0	990 ± 30.0
**6**	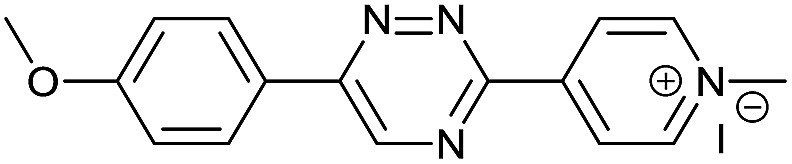	9.1 ± 0.3	190 ± 6.0	640 ± 20.0
**7**	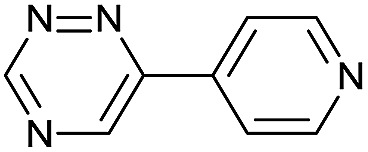	79.0 ± 5.0	940 ± 20.0	2020 ± 32.0
**8**	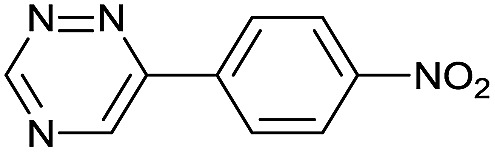	84.0 ± 7.0	1130 ± 50.0	2450 ± 70.0

^*a*^All reactions were performed in H_2_O/CH_3_CN = 1/1 at room temperature under pseudo first-order conditions using an excess of the corresponding TCO.

The data show that the presence of an electron donating *para*-methoxy substituent in **2** led to a slight decrease in reactivity, consistent with the inverse electron-demand nature of the cycloaddition. In contrast, the presence of the pyridinium group in **6** resulted in a greater than five-fold increase in reaction rate when compared to the analogous derivative bearing an unsubstituted pyridyl group (**3**). The position of the alkylation turned out to be very important. Triazine **5** bearing the alkyl group at the *para* position is about 30-times more reactive than the corresponding *ortho*-alkylated derivative **4**. This indicates that the electron withdrawing ability of the substituent is compromised by the increased steric demand in this case. Our data show that 3,6-bisaryl substituted 1,2,4-triazines (**1–6**) react with TCOs slower than the corresponding mono-substituted 6-aryl derivatives (**7**, **8**). Although the previously reported compound **8** (ref. 7b) was found to be the most reactive among the series, this particular derivative cannot be further modified and thus its utility in bioconjugation reactions is limited. It is reasonable to assume that alkylation of the pyridyl group of compound **7** could further increase its reactivity. Unfortunately, our attempts to prepare the corresponding derivative were unsuccessful.

The structure and configuration of the TCO also plays an important role. Simple *trans*-cyclooctene-ols are the slowest reaction partners (Table 1 and S2†). The dioxolane-fused TCO (d-TCO) is known for its improved reactivity in IEDDA reaction with 1,2,4,5-tetrazines.^10^ This was also confirmed in our study and all triazines reacted with this dienophile about 10–20 times faster. A further increase in reactivity afforded the *trans*-bicyclononene derivative (s-TCO).3b,^10^ This compound led to an impressive increase (2–4 orders of magnitude) in reaction rate when compared to TCO or BCN derivatives used in previous studies.^7^ In fact, by reacting 1,2,4-triazines with d-TCO or s-TCO it is possible to reach reaction rates of the more reactive 1,2,4,5-tetrazines combined with less strained systems such as cyclopropene, norbornene or BCN derivatives, which are routinely employed in bioconjugation reactions.3e–g,5d,^11^

The excellent reactivity of pyridinium triazines drew our attention so we decided to study these compounds in more detail. We found that the pyridinium-modified 1,2,4-triazines are remarkably stable. Compound **6** was stable for over one week in PBS buffer at 37 °C even in the presence of an equimolar amount of l-cysteine (Fig. S5–S7†). In contrast, the most reactive 1,2,4,5-tetrazines are known to rapidly hydrolyze or degrade under similar conditions.7b,^9^,11a,^12^ However, it should be noted that the stability of tetrazines also strongly depends on the substitution pattern.^6^ The observed high reactivity of the presented 1,2,4-triazines is associated with the use of the most reactive TCOs (d-TCO or s-TCO) which isomerize to the much less reactive *cis* isomer more rapidly when compared to less strained TCOs.^10^ This may become a problem for certain applications of the developed chemistry. It is therefore important to consider this particular limitation and accordingly design and plan the experiment. Further development of highly reactive and stable TCO derivatives would obviously provide an ideal solution to this obstacle. First attempts toward this direction has already been reported.^13^

**Scheme 2:**
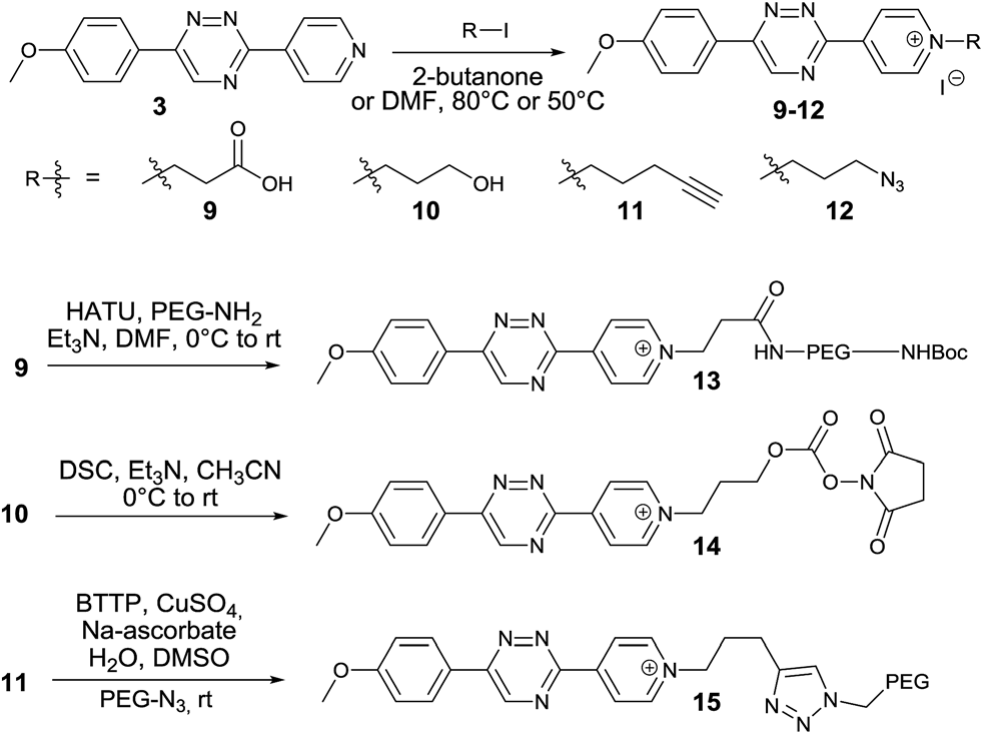
Synthesis of heterobifunctional pyridinium 1,2,4-triazines.

We next aimed to exploit the structural features of the pyridinium 1,2,4-triazines for the synthesis of heterobifunctional probes. The possibility of further derivatization is an important step toward successful use of these compounds in bioconjugation reactions, where the attachment of an additional functionality is often desirable. We thought that a late-stage alkylation of the pyridyl group would provide an elegant approach in this sense. To investigate if the alkylation can proceed with sufficient selectivity on the pyridine nitrogen atom we reacted **3** with 5 equivalents of 3-iodopropionic acid. To our delight the reaction afforded the desired pyridinium compound **9** in 71% isolated yield without noticeable alkylation of the triazine heterocycle. Using this strategy, we synthesized a series of *N*-alkylated pyridinium 1,2,4-triazines depicted in Scheme 2.

The carboxyl group of **9** can be used for peptide coupling under standard conditions. The hydroxyl group of **10** can be converted to the corresponding active ester **14**. The terminal alkyne group of **11** can be employed in the Cu(i)-catalyzed azide–alkyne cycloaddition reaction with azides (Scheme 2 and ESI†). The developed late-stage functionalization thus represents a simple and efficient synthetic methodology for preparation of various useful derivatives of 1,2,4-triazines.

**Scheme 3:**
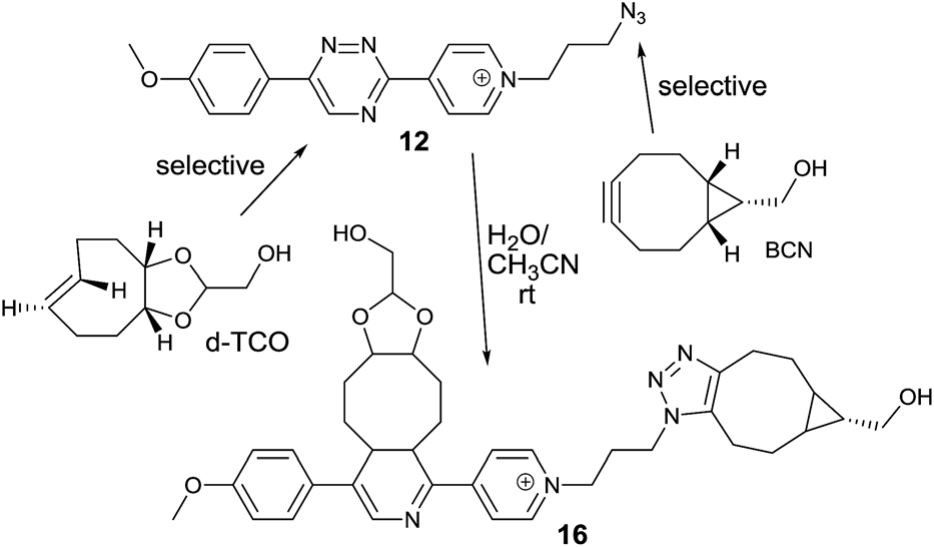
Single-step double click labeling of azido functionalized 1,2,4-triazine. Only one regioisomer is shown.

We next speculated that the low reactivity of the triazine scaffold toward BCN7*a* should allow for its use in a one-pot double click-labeling reaction in combination with the strain-promoted azide–alkyne cycloaddition.3*a* To explore this, we synthesized compound **12**. The azido group of **12** could in principle also react with the olefinic bond of TCO in a 1,3-dipolar cycloaddition. However, the reported second-order rate constant for a similar reaction^14^ was found to be ∼0.02 M^–1^ s^–1^ which is two orders of magnitude lower than the determined second-order rate constant of the reaction between **6** and *e.g.* d-TCO (1.9 M^–1^ s^–1^). We hypothesized that the difference in reactivity will provide the required selectivity. Indeed, reaction of **12** with d-TCO afforded the respective Diels–Alder product as confirmed by HPLC-MS. The azido group of the intermediate was subsequently modified with BCN to give **16** (Fig. S8†). Similarly, reaction of **12** with BCN provided only the 1,3-dipolar cycloaddition adduct that was further reacted with d-TCO to afford the same doubly modified product **16** (Fig. S9†). Reaction of **12** with a mixture of d-TCO and BCN afforded the intended doubly-modified product **16** in a single step (Scheme 3 and Fig. S10†). This experiment demonstrates that the difference in reactivity of the two functional groups of **12** can be exploited for an efficient sequential and even single-step double labeling by two metal-free bioconjugation reactions. The 1,2,4-triazine and the azide group thus represent a new type of mutually orthogonal–bioorthogonal functional groups with potential utility in *e.g.* double-labeling of biomolecules,^15^ simultaneous examination of multiple biological targets, or macromolecule assembly.15b,^16^

**Fig. 1:**
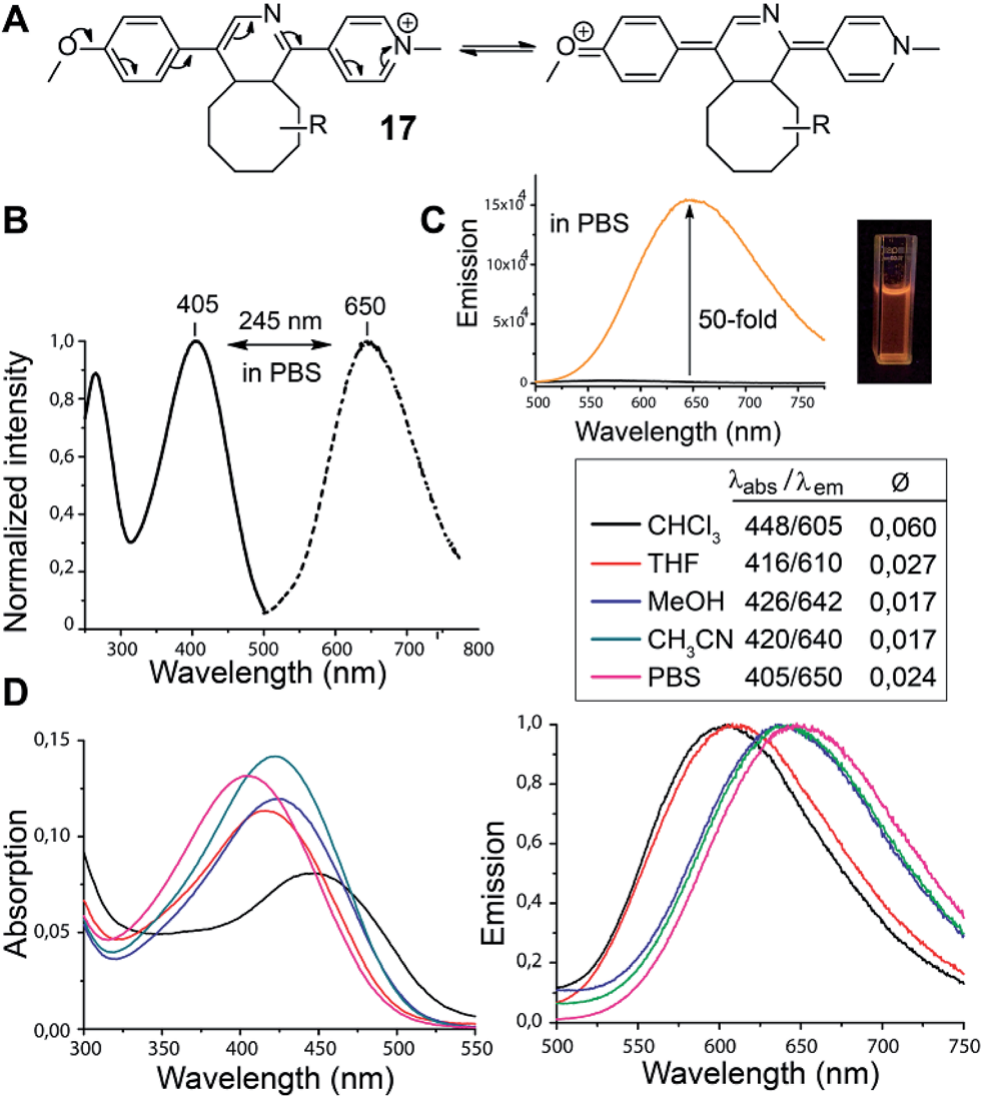
(A) Proposed formation of the push–pull fluorescent click product. R = dioxolane moiety of d-TCO (as drawn for compound **16** in Scheme 3). (B) Normalized absorption (solid line) and emission spectra (dashed line) of the click product in PBS buffer. (C) Fluorescence intensity increase upon click reaction (black line for **6** and orange line for **6** + d-TCO). (D) Absorption and emission spectra of **17** in different solvents. The quantum yields were determined using Rhodamine 6G as standard (*Ø* = 0.94 in EtOH).

In the course of our study we discovered that the pyridinium substituted 1,2,4-triazines produce fluorescent products in Diels–Alder reaction with various TCOs. Such reagents are essential for the development of so-called fluorogenic reactions.^17^ These reactions have great potential for bioimaging as they provide an improved signal-to-noise ratio.^18^ By simply mixing **6** with d-TCO in phosphate-buffered saline (PBS) a fluorescent product is formed with absorption and emission maxima at 405 nm and 650 nm respectively, yielding a new type of dye with an impressively large Stokes shift of 245 nm. We propose that the observed fluorescence results from the formation of a push–pull system arising from the initially formed dihydropyridine (Fig. 1). The structure of **17** was resolved by 1D and 2D NMR experiments (Fig. S11 and S12†). These experiments confirmed that it is the dihydropyridine and not the oxidized pyridine product that is fluorescent. As the starting triazine **6** is also not fluorescent one can conclude that the presence of a fully aromatic system abolishes the fluorescence even though a similar push–pull system can be drawn from these compounds as well.

**Fig. 2:**
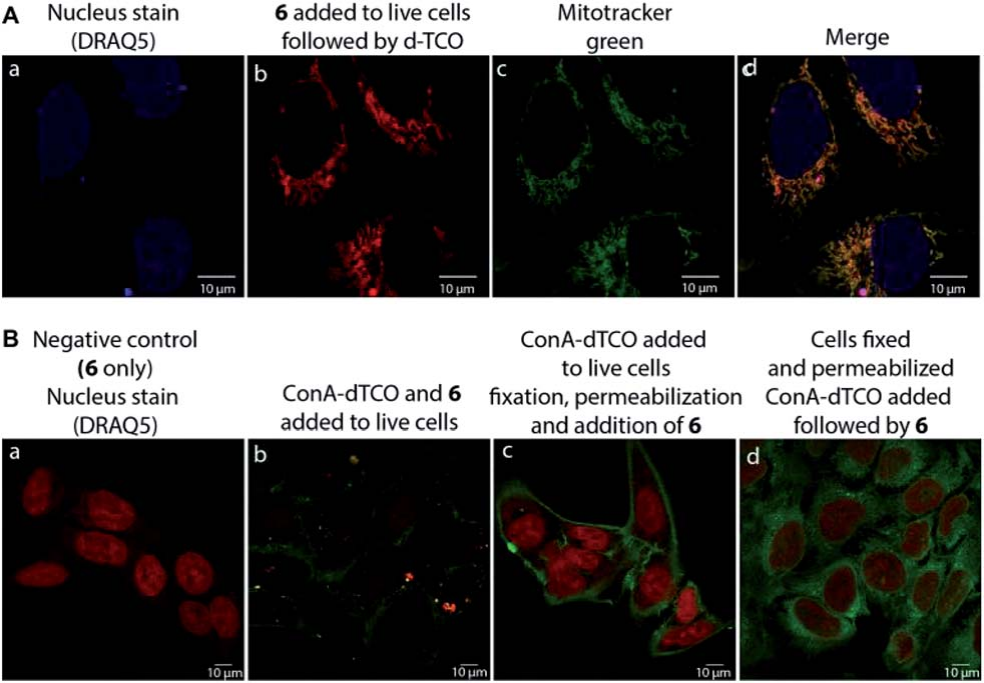
Confocal microscope images of (A) fluorogenic click labeling of mitochondria in live U2OS cells treated with **6** and subsequently with d-TCO; targeting of **6** to mitochondria was confirmed by co-localization experiment using Mitotracker green (c and d); (B) fluorogenic labeling using α-d-mannose- and α-d-glucose-specific ConA-dTCO conjugate: (a) negative control (cells treated only with **6**, nucleus staining with DRAQ5), (b) live cells labelling with ConA-dTCO followed by addition of **6**, (c) live cells incubated with ConA-dTCO, then fixed, permeabilized and treated with **6**, (d) cells fixed, permeabilized and treated with ConA-dTCO followed by **6**. Click product (ex.: 405 nm, em.: 560–666 nm); DRAQ5 (ex.: 633 nm, em.: 653–732 nm); Mitotracker green (ex.: 496 nm, em.: 505–588 nm). Pictures were processed using LAS AF Lite program.

We found that **17** is reasonably stable and only slowly started to oxidize/decompose over the course of a couple of days during incubation in PBS/CH_3_CN mixture at room temperature in the presence of air (Fig. S14†). We also found that **17** is a solvatochromic fluorophore with emissions ranging from 605 to 650 nm depending on the polarity of the solvent (Fig. 1D and S18†). In comparison to other commonly used fluorophores **17** has relatively low quantum yield. However, the strong increase in fluorescence upon the Diels–Alder reaction with TCOs (50-fold) together with the absorption maxima at 405 nm, which is a standard excitation wavelength used in *e.g.* confocal scanning microscopy, makes **17** a potential candidate for bioimaging applications. We therefore decided to investigate if the fluorogenic properties of the reaction will be preserved under biological conditions. We speculated that **6** as a positively charged and lipophilic molecule could be directly targeted to mitochondria.^19^ To verify this, we incubated live U2OS cancer cells with **6** and then added d-TCO to initiate the fluorogenic reaction. We chose d-TCO instead of the more reactive s-TCO because of its improved stability under biological conditions.^10^ When inspecting the cells under a confocal microscope, no fluorescence formed in cells treated only with **6**, while fluorescence was clearly visible in the live cells after addition of d-TCO (Fig. 2A-b and S19†). The targeting of **6** to mitochondria was confirmed by co-staining experiment using commercially available Mitotracker green (Fig. 2A and S20†). The inherent structural properties of **6** thus make this compound a new type of cell permeable, mitochondria-selective, chemically-activatable fluorogenic probe which may offer unique opportunities for dissecting the function of this vital cellular organelle.^20^

To further probe the potential of **6** for bioimaging, we conjugated the d-TCO moiety to concanavalin A (ConA-dTCO), a lectin with high specificity for α-d-mannose- and α-d-glucose-containing glycoconjugates.^21^ We first added ConA-dTCO to live U2OS cells where it binds glycoconjugates on the cell membrane. Addition of **6** directly to live cells resulted into only a relatively weak fluorescent signal (Fig. 2B-b and S21†). However, after fixation and permeabilization, addition of **6** yielded a clear staining of the cellular membrane (Fig. 2B-c and S22†). In addition, when the cells were fixed and permeabilized first and subsequently incubated with ConA-dTCO, the addition of **6** resulted into perspicuous fluorescent staining of the internal glycosylated compartments (Fig. 2B-d and S23†). These experiments clearly express that the reaction proceeds efficiently under biological conditions and that the fluorogenic nature of the reaction can be used for bioimaging.

## Conclusions

In conclusion, our systematic study on the reactivity of various 1,2,4-triazines with TCOs in the IEDDA reaction shows that the reaction rates strongly depend on the substitution pattern of both the 1,2,4-triazine and the TCO. This study led to the discovery of novel *N*-alkyl pyridinium triazines with superb properties for use in bioconjugate chemistry. We have developed an efficient and modular synthetic strategy which enables the construction of various useful heterobifunctional probes based on the pyridinium 1,2,4-triazine scaffold. In addition, we describe the unprecedented fluorogenic nature of the reaction between these heterodienes and TCOs and demonstrate the potential of the chemistry for fluorogenic cell labeling. With this study, we have demonstrated pyridinium 1,2,4-triazines to be versatile and robust reagents with a prospective future in chemical biology and bioimaging. Further applications of these unique heterodienes for labeling of biomolecules as well as work toward improvement and modulation of their fluorogenic properties are currently underway.

## Supplementary Material

Supplementary information
