# Supplementary material for: The discovery of pyridinium 1,2,4-triazines with enhanced performance in bioconjugation reactions
Source: Chem Sci. 2017 Mar 1;8(5):3593–8. doi: 10.1039/c6sc05442k (PMC6092722; doi:10.1039/c6sc05442k)
Supplement: Supplementary file 1 [file SC-008-C6SC05442K-s001.pdf]

Electronic Supplementary Material (ESI) for Chemical Science.
This journal is © The Royal Society of Chemistry 2017

The Discovery of Pyridinium 1,2,4-Triazines with Enhanced Performance in Bioconjugation Ractions

Sebastian J. Siegl, Rastislav Dzijak, Arcadio Vázquez, Radek Pohl and Milan Vrabel

Institute of Organic Chemistry and Biochemistry of the Czech Academy of Sciences

vrabel@uochb.cas.cz

Supporting Information

Content

General information 2

Synthetic procedures........ 2

Determination of the second-order rate constants 13

Stability studies of compound 6 17

Double click-labeling of 12 by two orthogonal metal-free bioconjugations........ 19

Synthesis and characterization of the click product 17........ 22

Stability of the click product 17 27

Photophysical properties of the click product 17 28

Cell labeling experiments 32

References........ 35

Copies of NMR spectra 36

General information

All chemicals were obtained from *Sigma Aldrich*, *Alfa Aesar*, *Acros Organics*, *ABCR* or *VWR* and were used without further purification. Reactions with air- and moisture-sensitive reactants were performed under nitrogen- or argon- atmosphere and in anhydrous solvents from *VWR*.

Solutions were concentrated on a rotary evaporator from *Heidolph* equipped with a PC3001 VARIOpro pump from *Vacuubrand*. Photochemical reactions were performed in a RPR-200 Rayonet reaction chamber equipped with 16 Hg-quartz iodine lamps (2537 Å) from *Southern New England Ultraviolet Company*. The continuous flow system during the photoreaction was produced by a STEPDOS 03 RC membrane-metering pump from *KNF*. Column chromatography was carried out on silica gel 60A (particle size: 40-60 µm) from *Acros Organics*. Solvents in the p.a. quality from *Lech-Ner* and *Penta* were used for elution. Mixtures of solvents are each stated as volume fractions. For flash column chromatography a Combi*Flash*® Rf+ from *Teledyne ISCO* was used. Thin-layer chromatography was performed on aluminium sheets from *Merck* (silica gel 60 F254, 20x20 cm). Chromatograms were visualized by UV light ($\lambda = 254$ nm/ 366 nm) or by staining with $KMnO_4$ or anisaldehyde solutions. ^{1}H- and ^{13}C-NMR spectra were measured on a Bruker Avance III™ HD 400 MHz NMR system equipped with Prodigy cryo-probe or on a Bruker Avance III™ HD 500 MHz Cryo. $CDCl_3$, MeOH-d_4 and DMSO-d_6 from *Sigma Aldrich* or *Eurisotop* were used as solvents. Chemical shifts δ are quoted in ppm in relation to the chemical shift of the residual non-deuterated solvent peak ($CDCl_3$: $\delta(^{1}H) = 7.26$, $\delta(^{13}C) = 77.2$; MeOH-d_4: $\delta(^{1}H) = 3.31$, $\delta(^{13}C) = 49.0$; DMSO-d_6: $\delta(^{1}H) = 2.50$, $\delta(^{13}C) = 39.5$). High-resolution mass spectra were recorded on an *Agilent* 5975C MSD Quadrupol, Q-Tof micro from *Waters* or LTQ Orbitrap XL from *Thermo Fisher Scientific*. UV/VIS spectroscopy was performed on a Cary 60 UV/Vis spectrophotometer from *Agilent Technologies*. HPLC-MS measurements were performed on a LCMS-2020 system from *Shimadzu* equipped with a Luna® C18 column (3u, 100A, 100 x 4.6 mm). The samples were eluted using a linear gradient of CH_3CN + 0.05% HCOOH (5→95% in 9 min) in H_2O + 0.05% HCOOH at a flow rate of 1.0 mL/min. Data from kinetic experiments were processed using OriginPro 9.1 software.

Synthetic procedures

The following compounds were prepared according to literature procedures.

trans-cyclooct-4-enol[1]

((2s,3a*R*,9a*S*,*E*)-3a,4,5,8,9,9a-hexahydrocycloocta[*d*][1,3]dioxol-2-yl)methanol[2]

(*rel*-1*R*,8*S*,9*R*,4*E*)-Bicyclo[6.1.0]non-4-ene-9-yl-methanol[3]

4-methoxyphenylglyoxal[4]

2-oxo-2-(4-methoxyphenyl)acetaldehyde oxime[5]

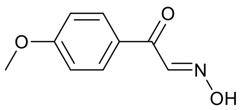

To a solution of 4-methoxyphenylglyoxal (1.73 g, 10.5 mmol) in EtOH (25 mL) H_2O (10 mL), hydroxylamine hydrochloride (879 mg, 12.6 mmol) and anhydrous sodium carbonate (670 mg, 6.32 mmol) were added at 0 °C. After one hour at room temperature the mixture was diluted with brine (50 mL) and then extracted with EtOAc (2x 100 mL). The combined organic layers were washed with sat. aq. $NaHCO_3$ (70 mL), dried over Na_2SO_4 and concentrated *in vacuo*. The crude oxime (1.63 g) was used directly in the next step without further purification.

2-hydrazono-2-(4-methoxyphenyl)acetaldehyde oxime[6]

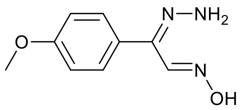

To a solution of 2-oxo-2-(4-methoxyphenyl)acetaldehyde oxime (1.63 g, 9.10 mmol) in EtOH (25 mL) hydrazine monohydrate (758 µL, 10.0 mmol) and a few drops of acetic acid were added at 45 °C. The solution was stirred at 45 °C for 15 h. After the starting material has disappeared, the crude product was concentrated *in vacuo* and purified by flash column chromatography (eluting with 0-25% EtOAc in CH_2Cl_2 + 1% MeOH) to provide the oximinohydrazone as an orange solid (1.02 mg, 58%).
The identity of the purified product was verified by HPLC-MS on a Luna® C18 column (3u, 100A, 100 x 4.6 mm) using a linear gradient of CH_3CN (5-95% in 9 min and 1mL/min flow) in H_2O + 0.05% HCOOH. The purification provided two isomers of the oximinohydrazone eluting at 6.42 min and 8.06 min, respectively (Figure S1).
MS (ESI): m/z calcd. for $C_9H_{12}N_3O_2$ $[MH]^+$ 194.1, found 194.1.

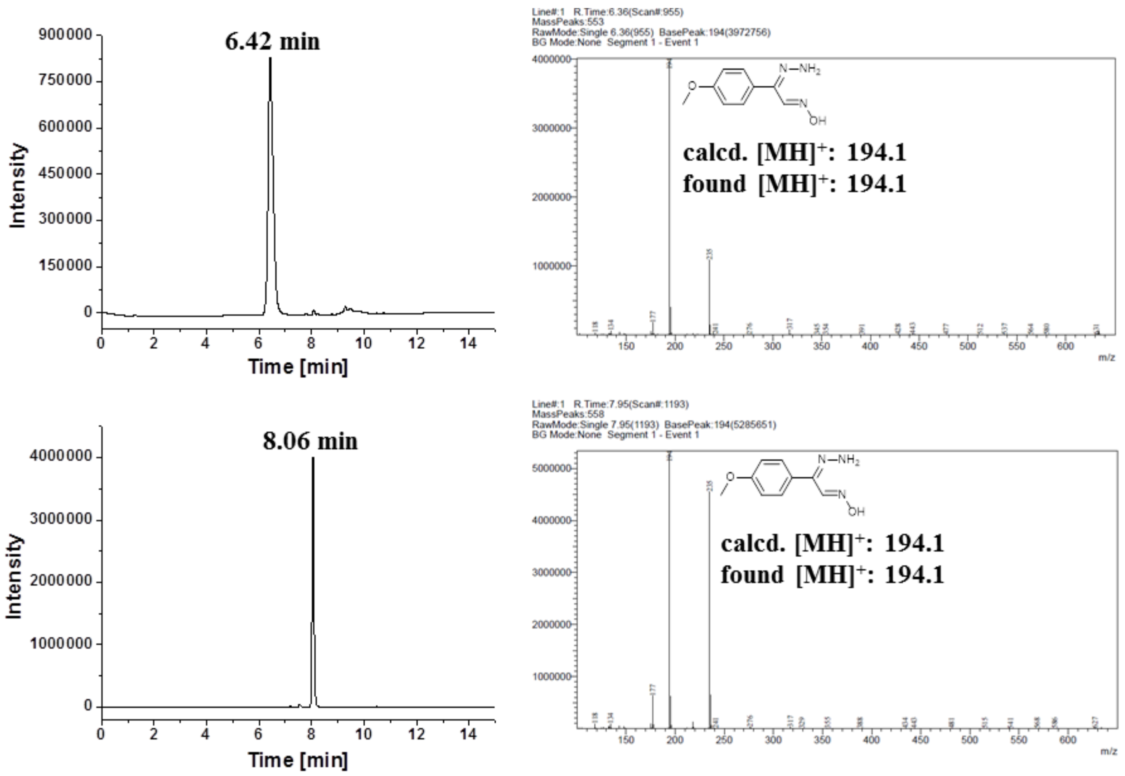

Figure S1. HPLC-MS analysis of the two isomers of 2-hydrazono-2-(4-methoxyphenyl)acetaldehyde oxime obtained after purification by flash column chromatography.

<u>2-hydrazono-2-phenylacetaldehyde oxime</u>[6]

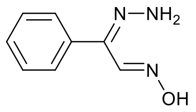

2-Isonitrosoacetophenone (2.50 g, 16.8 mmol) and a few drops of acetic acid were added to a solution of hydrazine monohydrate (1.27 mL, 16.8 mmol) in EtOH (25 mL) at 45 °C. The mixture was stirred at 45 °C for 20 h. After the starting material has disappeared, the crude product was concentrated *in vacuo* and purified by flash column chromatography (eluting with 0-20% EtOAc in CH_2Cl_2 + 1% MeOH) to provide the oximinohydrazone as a yellow solid (2.26 g, 82%).
Analytical data matched with literature.[7]

<u>3-(2-pyridyl)-6-phenyl-1,2,4-triazine</u> (1)[6]

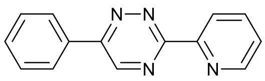

To a solution of 2-hydrazono-2-phenylacetaldehyde oxime (167 mg, 1.02 mmol) in EtOH (7 mL) a few drops of acetic acid and 2-pyridinecarboxaldehyde (117 µL, 1.23 mmol) were added. The solution was stirred at room temperature for 21 h. The formed bisaryl substituted intermediate was concentrated *in vacuo*, dissolved in acetic acid (2 mL) and heated at 100 °C under reflux for 1 h. After the complete intermediate was cyclized (verified by HPLC-MS),

the mixture was concentrated *in vacuo*, diluted with H_2O (10 mL) and the pH was adjusted to ~8 by addition of sat. aq. $NaHCO_3$. The suspension was extracted with CH_2Cl_2 (3x 60 mL) and the combined organic layers were washed with brine (70 mL), dried over Na_2SO_4 and concentrated *in vacuo*. The crude triazine was purified by flash column chromatography (eluting with 0-30% MeOH in CH_2Cl_2) and further by recrystallization from EtOAc to provide **1** as a light-brown solid (154 mg, 64%).
HRMS (EI+): m/z calcd. for $C_{14}H_{10}N_4$ $[MH]^+$ 234.0905, found 234.0904. Spectral data matched with literature.[6]

3-(2-pyridyl)-6-(4-methoxyphenyl)-1,2,4-triazine (2)[6]

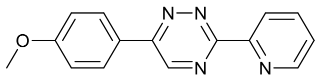

A few drops of acetic acid and 2-pyridinecarboxaldehyde (292 µL, 3.07 mmol) were added to a solution of 2-hydrazono-2-(4-methoxyphenyl)acetaldehyde oxime (362 mg, 1.87 mmol) in EtOH (10 mL). The solution was stirred at room temperature for 20 h. The formed bisaryl substituted intermediate was concentrated *in vacuo*, dissolved in acetic acid (5 mL) and heated at 100 °C under reflux for 1 h. After the complete intermediate was cyclized (verified by HPLC-MS), the mixture was concentrated *in vacuo*, diluted with H_2O (10 mL) and the pH was adjusted to ~8 by addition of sat. aq. $NaHCO_3$. The suspension was extracted with CH_2Cl_2 (3x 60 mL) and the combined organic layers were washed with brine (70 mL), dried over Na_2SO_4 and concentrated *in vacuo*. The crude product was purified by flash column chromatography (eluting with 0-90% EtOAc in CH_2Cl_2 + 1% MeOH) and recrystallization from EtOAc to provide **2** as a brown solid (339 mg, 69%).
HRMS (EI+): m/z calcd. for $C_{15}H_{12}N_4O$ $[MH]^+$ 264.1011, found 264.1009. Spectral data matched with literature.[6]

3-(4-pyridyl)-6-(4-methoxyphenyl)-1,2,4-triazine (3)

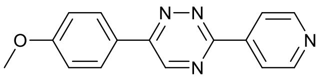

To a solution of 2-hydrazono-2-(4-methoxyphenyl)acetaldehyde oxime (420 mg, 2.17 mmol) in EtOH (15 mL) were added a few drops of acetic acid and 4-pyridinecarboxaldehyde (205 µL, 2.17 mmol). The solution was stirred at room temperature for 28 h. The formed bisaryl substituted intermediate was concentrated *in vacuo*, dissolved in acetic acid (6 mL) and heated at 100 °C under reflux for 1 h. After the complete intermediate was cyclized (verified by HPLC-MS), the mixture was concentrated *in vacuo*, diluted with H_2O (10 mL) and the pH was adjusted to ~8 by addition of sat. aq. $NaHCO_3$. The suspension was extracted with CH_2Cl_2 (3x 60 mL) and the combined organic layers were washed with brine (70 mL), dried over Na_2SO_4 and concentrated *in vacuo*. The crude triazine was purified by flash column chromatography (eluting with 0-65% EtOAc in CH_2Cl_2 + 1% MeOH) to provide **3** as a yellow solid (316 mg, 55%).

^{1}H NMR (401 MHz, $CDCl_3$): δ 9.06 (s, 1H), 8.88 -8.79 (m, 2H), 8.42-8.35 (m, 2H), 8.20-8.08 (m, 2H), 7.16-7.04 (m, 2H), 3.91 (s, 3H).
^{13}C NMR (101 MHz, $CDCl_3$): δ 162.56, 160.27, 155.82, 150.90, 146.13, 142.25, 128.57, 125.20, 121.62, 115.12, 55.65.
HRMS (EI+): m/z calcd. for $C_{15}H_{12}N_4O$ $[MH]^+$ 264.1011, found 264.1009.

2-formyl-1-methylpyridinium iodide[8]

4-formyl-1-methylpyridinium iodide[8]

1-methyl-2-(6-phenyl-1,2,4-triazin-3-yl)pyridinium iodide (4)

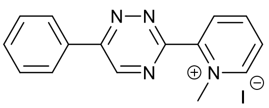

2-formyl-1-methylpyridinium iodide (382 mg, 1.53 mmol) and acetic acid (250 µL) were added to a solution of 2-hydrazono-2-phenylacetaldehyde oxime (250 mg, 1.53 mmol) in EtOH (10 mL). The solution was stirred at room temperature for 23 h. The formed bisaryl substituted intermediate was concentrated *in vacuo*, dissolved in acetic acid (3 mL) and heated at 100 °C under reflux for 3 h. After the complete intermediate was cyclized (verified by HPLC-MS), the mixture was concentrated *in vacuo*, diluted with H_2O (10 mL) and the pH was adjusted to ~8 by addition of sat. aq. $NaHCO_3$. The suspension was extracted with *i*PrOH/CH_2Cl_2 (2:1) (3x 60 mL) and the combined organic layers were washed with brine (70 mL), dried over Na_2SO_4 and concentrated *in vacuo*. The crude triazine was purified twice by flash column chromatography (first eluting with 10% H_2O in CH_3CN and second with 5-60% MeOH in H_2O on a reversed phase C18 column) to provide **4** as a yellow solid (61 mg, 11%).
^{1}H NMR (401 MHz, MeOH-d_4): δ 9.60 (s, 1H), 9.20 (d, J = 6.2 Hz, 1H), 8.83 (td, J = 7.8, 1.4 Hz, 1H), 8.76 (dd, J = 8.1, 1.7 Hz, 1H), 8.40-8.33 (m, 2H), 8.30 (ddd, J = 7.8, 6.1, 1.7 Hz, 1H), 7.72-7.63 (m, 3H), 4.70 (s, 3H).
^{13}C NMR (101 MHz, MeOH-d_4): δ 159.58, 158.14, 150.44, 149.90, 148.90, 147.48, 133.69, 133.26, 132.26, 130.79, 130.70, 130.12, 128.79.
HRMS (ESI): m/z calcd. for $C_{15}H_{13}N_4$ $[MH]^+$ 249.1135, found 249.1135.

1-methyl-4-(6-phenyl-1,2,4-triazin-3-yl)pyridinium iodide (5)

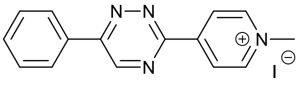

To a solution of 2-hydrazono-2-phenylacetaldehyde oxime (250 mg, 1.53 mmol) in EtOH (10 mL) were added a few drops of acetic acid and 4-formyl-1-methylpyridinium iodide (382 mg, 1.53 mmol). After stirring the solution at room temperature for 9 h, additional acetic acid (250 µL) was added and the mixture was stirred again at room temperature for 19 h. The mixture was concentrated *in vacuo*, diluted with H_2O (10 mL) and the pH was adjusted to ~8

by addition of sat. aq. $NaHCO_3$. The suspension was extracted with *i*PrOH/CH_2Cl_2 (2:1) (3x 60 mL) and the combined organic layers were washed with brine (70 mL), dried over Na_2SO_4 and concentrated *in vacuo*. Purification by flash column chromatography (eluting with 10% H_2O in CH_3CN on normal phase silica gel) provided **5** as a yellow solid (226 mg, 39%).

^{1}H NMR (401 MHz, MeOH-d_4): δ 9.54 (s, 1H), 9.15 (d, J = 6.9 Hz, 2H), 9.09 (d, J = 7.0 Hz, 2H), 8.36-8.29 (m, 2H), 7.69-7.61 (m, 3H), 4.54 (s, 3H).

^{13}C NMR (101 MHz, MeOH-d_4): δ 159.35, 158.56, 151.61, 149.22, 147.78, 133.89, 133.10, 130.64, 128.65, 126.54.

HRMS (ESI): m/z calcd. for $C_{15}H_{13}N_4$ $[MH]^+$ 249.1135, found 249.1136.

4-(6-(4-methoxyphenyl)-1,2,4-triazin-3-yl)-1-methylpyridinium iodide (6)

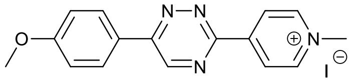

To a solution of 2-hydrazono-2-(4-methoxyphenyl)acetaldehyde oxime (300 mg, 1.55 mmol) in EtOH (10 mL) were added a few drops of acetic acid and 4-formyl-1-methylpyridinium iodide (387 mg, 1.55 mmol). After stirring the solution at room temperature for 20 h, additional acetic acid (250 µL) was added and the mixture was stirred again at room temperature for 23 h. The mixture was concentrated *in vacuo*, diluted with H_2O (10 mL) and the pH was adjusted to ~8 by addition of sat. aq. $NaHCO_3$. The suspension was extracted with *i*PrOH/CH_2Cl_2 (2:1) (3x 60 mL) and the combined organic layers were washed with brine (70 mL), dried over Na_2SO_4 and concentrated *in vacuo*. The crude triazine was purified by flash column chromatography (eluting with 10% H_2O in CH_3CN on normal phase silica gel) to provide **6** as a yellow-brown solid (264 mg, 42%).

^{1}H NMR (401 MHz, DMSO-d_6): δ 9.67 (s, 1H), 9.24 (dd, J = 6.4, 1.1 Hz, 2H), 8.97-8.88 (m, 2H), 8.38-8.27 (m, 2H), 7.25-7.14 (m, 2H), 4.48 (s, 3H), 3.88 (s, 3H).

^{13}C NMR (101 MHz, DMSO-d_6): δ 162.52, 157.27, 155.88, 148.97, 147.70, 146.81, 129.31, 124.74, 124.53, 115.13, 55.70, 48.04.

HRMS (ESI): m/z calcd. for $C_{16}H_{15}N_4O$ $[MH]^+$ 279.1240, found 279.1241.

3-amino-6-bromo-1,2,4-triazine

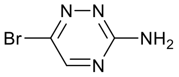

3-amino-6-bromo-1,2,4-triazine was prepared according to a slightly modified literature procedure with an additional purification step by flash column chromatography (eluting with 0-90% EtOAc in CH_2Cl_2 + 1% MeOH) at the end.[9]

Analytical data matched with literature.[10]

6-(4-nitrophenyl)-1,2,4-triazine (8)

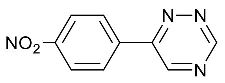

6-(4-nitrophenyl)-1,2,4-triazine was prepared according to a slightly modified literature procedure.[10]

Following the literature procedure, conditions of the Suzuki coupling were modified as follows: the reaction was performed in DMF/H_2O (4:1) mixture and K_2CO_3 was used as base instead of Cs_2CO_3.

Analytical data matched with literature.[10]

6-(4-pyridyl)-1,2,4-triazine (7)

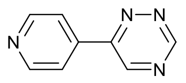

Following the literature procedure,[10] 3-amino-6-bromo-1,2,4-triazine (500 mg, 2.86 mmol), 4-pyridinyl-boronic acid (421 mg, 3.43 mmol) and $Pd(PPh_3)_4$ (165 mg, 0,143 mmol) were used. Unlike in the literature, the Suzuki coupling was performed in DMF/H_2O (4:1) (25 mL) and K_2CO_3 (592 mg, 4.29 mmol) was used as base instead of Cs_2CO_3. The extracted intermediate was purified by flash column chromatography (eluting with 0-10% MeOH in CH_2Cl_2). In the second step 7.5 eq. of isopentyl nitrite (1.84 mL, 13.7 mmol) were added step by step over several hours until the intermediate has disappeared (verified by HPLC-MS). The crude product was purified by flash column chromatography (eluting with 0-10% MeOH in CH_2Cl_2) to provide **7** as an orange solid (53 mg, 19%).

^{1}H NMR (401 MHz, $CDCl_3$): δ 9.76 (s, 1H), 9.09 (s, 1H), 8.88-8.85 (m, 2H), 8.03-7.99 (m, 2H).

^{13}C NMR (101 MHz, $CDCl_3$): δ 157.35, 156.07, 151.20, 146.81, 140.52, 120.85.

HRMS (EI+): m/z calcd. for $C_8H_6N_4$ $[MH]^+$ 158.0592, found 158.0591.

1-(2-carboxyethyl)-4-(6-(4-methoxyphenyl)-1,2,4-triazin-3-yl)pyridinium formate (9)

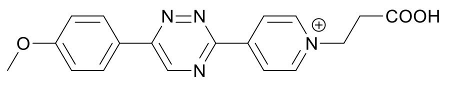

To a suspension of **3** (50 mg, 0.19 mmol) in 2-butanone (2.5 mL) was added 3-iodo-propionic acid (189 mg, 0.95 mmol, 5 eq.) and the reaction mixture was stirred at 80 °C overnight. The solvent was removed *in vacuo* and the product was isolated by normal phase silica gel column chromatography (eluting with CH_3CN/H_2O (4:1) → CH_3CN/H_2O (4:1) + 0.25% HCOOH). The product was isolated as yellow powder and as salt of formic acid (51 mg, 71%).

^{1}H NMR (400 MHz, CD_3CN/D_2O (1:1)): δ 9.35 (s, 1H), 9.00 (d, *J* = 6.8 Hz, 2H), 8.88 (d, *J* = 6.7 Hz, 2H), 8.19 (d, *J* = 8.9 Hz, 2H), 7.15 (d, *J* = 8.9 Hz, 2H), 4.79 (t, *J* = 6.6 Hz, 3H), 3.85 (s, 2H), 2.87 (t, *J* = 6.6 Hz, 2H).

^{13}C NMR (100 MHz, CD_3CN/D_2O (1:1)): δ 172.5, 162.8, 157.2, 156.4, 150.3, 147.7, 146, 129.2, 125.3, 124.4, 115.1, 56.9, 55.4, 34.2.
HRMS (ESI): m/z calcd. for $C_{18}H_{17}O_3N_4$ $[MH]^+$ 337.1295, found 337.1296.

1-(3-hydroxypropyl)-4-(6-(4-methoxyphenyl)-1,2,4-triazin-3-yl)pyridinium chloride (10)

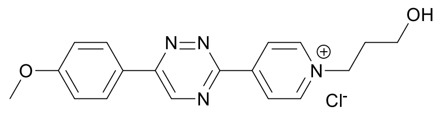

To a suspension of **3** (25 mg, 0.095 mmol) in 2-butanone (1 mL) was added 4-iodo-propanol (45 µL, 0.47 mmol, 5 eq.) and the reaction mixture was stirred at 100 °C for 1 h and then at 80 °C overnight. The solvent was removed under vacuum and the product was isolated by normal phase silica gel column chromatography (eluting with CH_3CN/H_2O (10:1) + 0.1% of sat. aq. NH_4PF_6). Fractions containing the product were collected, evaporated and the solid was transferred into 2 mL polypropylene tube. The solid was repeatedly washed with H_2O (3x 1mL) to remove excess of NH_4PF_6. After each washing step the tube was centrifuged and the liquid discarded. This product (as PF_6^- salt) was further purified by second silica gel column (eluting with DCM/MeOH = 9:1). The product was isolated as a yellow powder (33 mg, 75%). The PF_6^- salt (soluble in CH_3CN or DCM) can be easily converted to the corresponding water soluble Cl^- salt by passing the product through short column of Dowex 50 1X2 in Cl^- form (elution with H_2O) and lyophilization.
^1H NMR (400 MHz, CD_3CN/D_2O (1:1)): δ 9.33 (s, 1H), 8.98 (d, *J* = 6.7 Hz, 2H), 8.88 (d, *J* = 7.0 Hz, 2H), 8.28 (d, *J* = 9.1 Hz, 2H), 7.18 (d, *J* = 9.0 Hz, 2H), 4.83-4.65 (m, 2H), 3.61 (q, *J* = 5.5 Hz, 2H), 2.91 (t, *J* = 4.9 Hz, 2H), 2.18 (m, 2H).
^{13}C NMR (100 MHz, CD_3CN/D_2O (1:1)): δ 164.0, 158.2, 157.5, 151.5, 148.1, 146.6, 130.2, 126.3, 125.6, 116.0, 60.3, 58.4, 56.3, 33.9.
HRMS (ESI): m/z calcd. for $C_{18}H_{19}O_2N_4$ $[MH]^+$ 323.1503, found 323.1503.

4-(6-(4-methoxyphenyl)-1,2,4-triazin-3-yl)-1-(pent-4-yn-1-yl)pyridinium iodide (11)

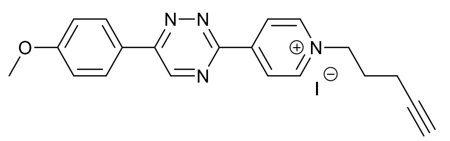

To a Schlenk tube containing a solution of triazine **3** (50 mg, 0.189 mmol) in anhydrous DMF (4 mL) was added step by step over several hours 5-iodo-1-pentyne (312 mg, 1.61 mmol) until the starting material has disappeared (verified by HPLC-MS). The reaction was performed under argon and at 50 °C. The crude product was concentrated *in vacuo* and purified by silica gel column chromatography (eluting with CH_2Cl_2/MeOH (20:1) → (10:1) → (5:1)) to provide the alkylated triazine **11** as an orange solid (58 mg, 67%).

^{1}H NMR (401 MHz, DMSO-d_6): δ 9.69 (s, 1H), 9.29 (d, *J* = 7.0 Hz, 2H), 8.99 (d, *J* = 6.9 Hz, 1H), 8.40-8.33 (m, 2H), 7.25-7.18 (m, 2H), 4.78 (t, *J* = 7.1 Hz, 2H), 3.89 (s, 3H), 2.88 (t, *J* = 2.6 Hz, 1H), 2.35 (td, *J* = 6.9, 2.4 Hz, 2H), 2.21 (p, *J* = 7.0 Hz, 2H).
^{13}C NMR (101 MHz, DMSO-d_6): δ 162.48, 157.16, 155.82, 149.45, 147.63, 146.10, 129.23, 125.09, 124.44, 115.06, 82.43, 72.51, 60.05, 55.62, 29.22, 14.85.
HRMS (ESI): m/z calcd. for $C_{20}H_{19}N_4O$ $[MH]^+$ 331.1553, found 331.1554.

<u>1-azido-3-iodopropane</u>

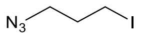

1-azido-3-iodopropane was prepared according to a slightly modified literature procedure.[11] Following the literature procedure, the crude product was purified by silica gel column chromatography (eluting with petroleum ether/EtOAc (4:1)) instead of distillation under reduced pressure.
Analytical data matched with literature.[11]

<u>1-(3-azidopropyl)-4-(6-(4-methoxyphenyl)-1,2,4-triazin-3-yl)pyridinium iodide</u> (12)

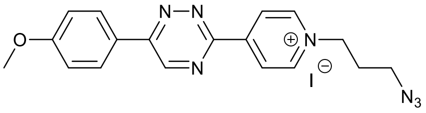

To a Schlenk tube containing a solution of triazine **3** (50 mg, 0.189 mmol) in anhydrous DMF (4 mL) was added 1-azido-3-iodopropane (200 mg, 0.946 mmol). The tube was flushed with argon and heated at 50 °C for 77 h. The crude product was concentrated *in vacuo* and purified by flash column chromatography (eluting with 0-10% MeOH in CH_2Cl_2) to provide the alkylated triazine **12** as an orange brown solid (75 mg, 83%).
^{1}H NMR (401 MHz, $CDCl_3$): δ 9.55-9.49 (m, 1H), 9.21 (s, 1H), 9.09-9.04 (m, 1H), 8.17 (d, *J* = 8.9 Hz, 1H), 7.07 (d, *J* = 9.0 Hz, 1H), 5.12 (t, *J* = 7.2 Hz, 1H), 3.89 (s, 2H), 3.64 (t, *J* = 6.2 Hz, 1H), 2.50-2.39 (m, 1H).
^{13}C NMR (101 MHz, $CDCl_3$): δ 163.34, 156.90, 156.57, 150.58, 146.55, 145.94, 129.34, 125.77, 124.14, 115.37, 59.51, 55.74, 48.08, 30.83.
HRMS (ESI): m/z calcd. for $C_{18}H_{18}N_7O$ $[MH]^+$ 348.1567, found 348.1569.

Synthesis of compound 13

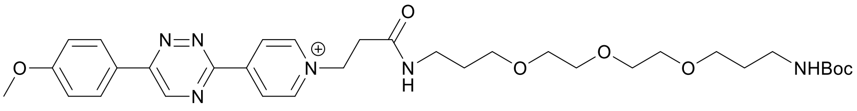

To an ice-water bath cooled suspension of **9** (10 mg, 0.026 mmol) in dry DMF (0.5 mL) was added *N*-Boc-4,7,10-trioxa-1,13-tridecanediamine (12.5 mg, 1.5 eq.) under argon followed by solid HATU (11 mg, 1.2 eq.) and DIPEA (11 µL, 2.5 eq.). The reaction mixture was stirred at room temperature under argon overnight. DMF was removed under vacuum and the residue

was purified by silica gel column chromatography (eluting with DCM/MeOH (9:1) → (5:1)). The product was isolated as yellow viscous oil (10 mg, 56%).
HRMS (ESI): m/z calcd. for $C_{33}H_{47}O_7N_6$ $[MH]^+$ 639.3501, found 639.3502.

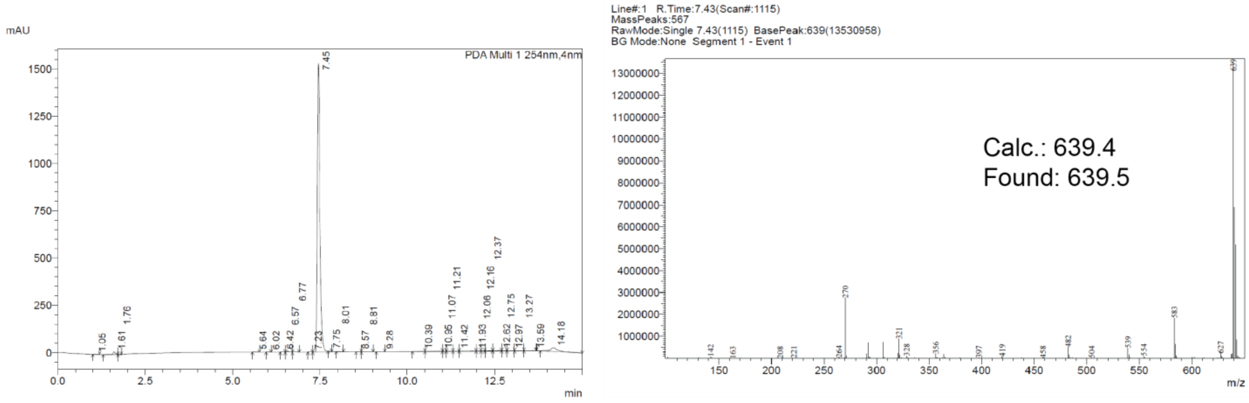

Figure S2. Exported HPLC-MS chromatograms of **13** (solvent A: H_2O + 0.05% HCOOH; solvent B: CH_3CN + 0.05% HCOOH; gradient: 5% B → 95% B in 9 min, then 2 min 95% B and back to 5% B). The product **13** eluates at 7.45 min (Luna® C18 column, 3u, 100A, 100 x 4.6 mm, 1 mL/min flow rate).

Synthesis of compound 14

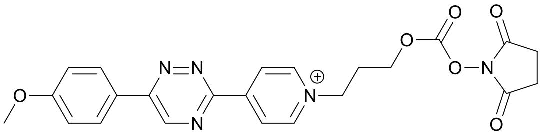

To an ice-cold solution of **10** (as PF_6^- salt, 15 mg, 0.032 mmol) in dry DMF (1 mL) was added solid disuccinimidyl carbonate (DSC, 16.4 mg, 2 eq.) under argon followed by Et_3N (18 µL, 4 eq.). The reaction mixture was stirred at room temperature until starting material disappeared (3 h, TLC in DCM/MeOH (9:1) and/or HPLC-MS). Our attempts to isolate the active ester by column chromatography led to partial decomposition of the product back to the starting material. However, the crude reaction mixture could be used directly in the next reaction step. The yield was ca. 80% (based on integrated peak area, Figure S3).

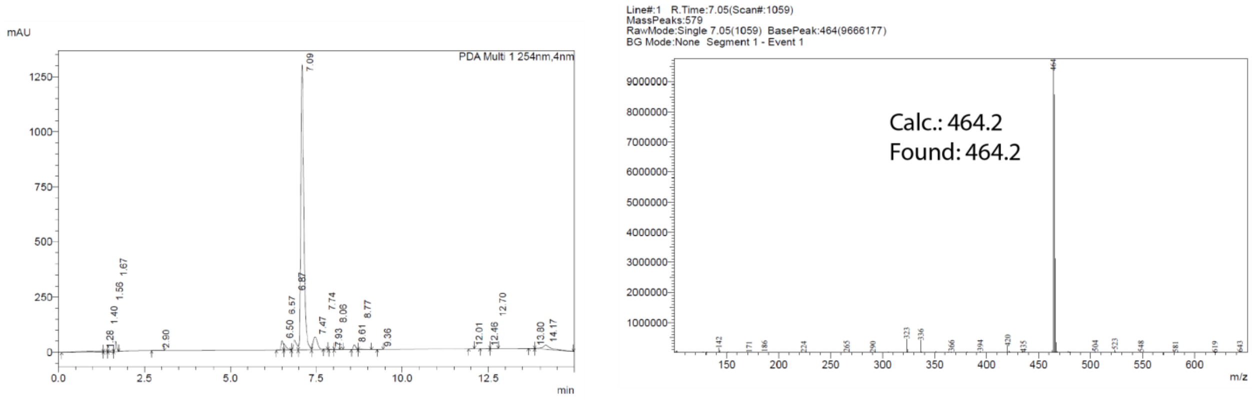

Figure S3. Exported HPLC-MS chromatograms of the crude reaction mixture after 3 h (solvent A: H_2O + 0.05% HCOOH; solvent B: CH_3CN + 0.05% HCOOH; gradient: 5% B → 95% B in 9 min, then 2 min 95% B and back to 5% B). Starting alcohol eluates at 6.35 min and product **14** at 7.1 min (Luna® C18 column, 3u, 100A, 100 x 4.6 mm, 1 mL/min flow rate).

Synthesis of compound 15

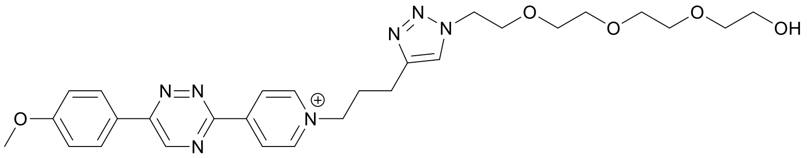

2-(2-(2-(2-azidoethoxy)ethoxy)ethoxy)ethan-1-ol (6 mg, 2.5 eq.) was added to a suspension of **11** (5 mg, 0.011 mmol) in H_2O/DMSO (3:2) (300 µL). In a separate tube aqueous Na-ascorbate solution (0.43 mg in 5 µL of H_2O, 20 mol%,) was added to a mixture of Cu-ligand BTTP[12] (1 mg, 20 mol%) and $CuSO_4$ .5 H_2O (0.3 mg, 10 mol%) in 10 µL of H_2O/DMSO (1:1). The colorless activated solution of the catalyst was added to the suspension of the alkyne and azide reagents and the reaction mixture was stirred at room temperature. The progress of the reaction was followed by HPLC-MS. After 2.5 h the reaction mixture did not change any longer giving 76% of the product (based on integrated peak area, Figure S4).

The formation of product was verified by HPLC-MS and HRMS.

HRMS (ESI): m/z calcd. for $C_{28}H_{36}O_5N_7$ $[M]^+$ 550.27724, found 550.27740.

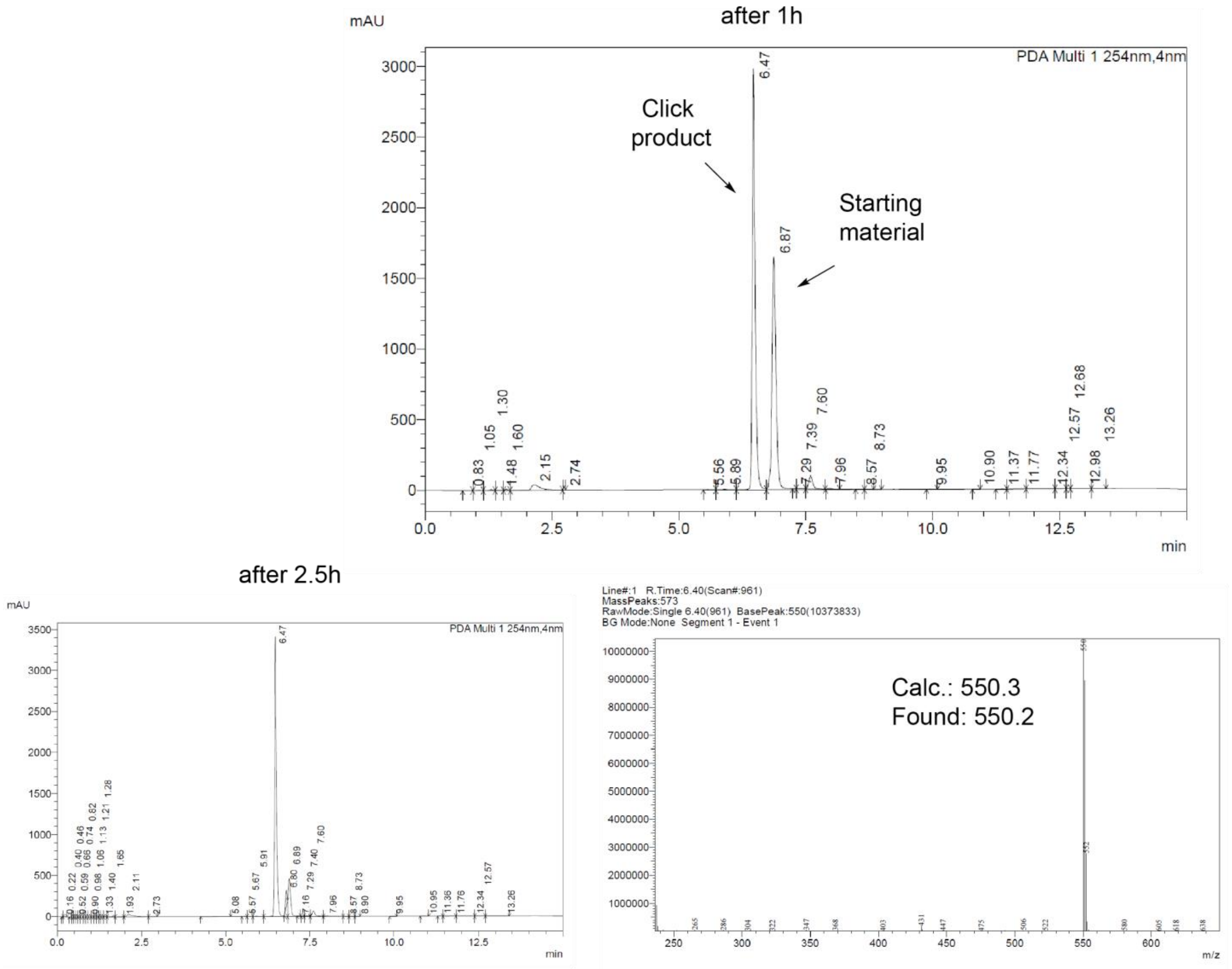

Figure S4. HPLC-MS chromatograms of the crude reaction mixture showing the formation of **15** after 1 h and 2.5 h respectively (solvent A: H_2O + 0.05% HCOOH; solvent B: CH_3CN + 0.05% HCOOH; gradient: 5% B → 95% B in 9 min, then 2 min 95% B and back to 5% B). Starting alkyne eluates at 6.9 min and the product **15** at 6.5 min (Luna® C18 column, 3u, 100A, 100 x 4.6 mm, 1 mL/min flow rate).

Determination of the second-order rate constants

Second order rate constants of the reactions between triazines and *trans*-cyclooctenes (TCO) were determined by following the decrease in the concentration of the starting 1,2,4-triazine over time. The concentration decrease was monitored either by HPLC (for slower derivatives) or by UV/VIS spectroscopy (for faster derivatives). The measurements were performed in a mixture of CH_3CN/H_2O (1:1) at room temperature under pseudo first-order conditions using an excess of the corresponding TCO. All runs were conducted at least three times.

For rate determination using HPLC: 20 µL of a 10 mM solution of the triazine in CH_3CN/H_2O (1:1) (10 µL for **6** + TCO-ol and 5 µL for **4** + d-TCO) were added to 80 µL of a 25 mM solution of the appropriate TCO (for **6** + TCO-ol to 90 µL of a 10 mM solution and for **4** + d-TCO to 20 µL of a 25 mM solution diluted with 75 µL of H_2O). The final

concentration of all triazines was 2 mM using 10 eq. of TCO (1 mM of triazine using 9 eq. of TCO for **6** + TCO-ol and 0.5 mM of triazine using 10 eq. of TCO for **4** + d-TCO). The measurements were performed on a Luna® C18 column (3u, 100A, 100 x 4.6 mm) using a linear gradient of CH_3CN + 0.05% HCOOH in H_2O + 0.05% HCOOH (5→95% in 9 min) at a flow rate of 1.0 mL/min. The MS device was disconnected from the HPLC during measurements. The integral of the absorption of the triazine at 254 nm was measured over 105 or 360 min in 15 or 45 min intervals. By using a calibration curve, the measured integrals were converted into the corresponding concentrations of the triazine, which were plotted against time to provide the observed rate constant from the slope of this plot (fitted with single exponential function: $y = y_0 + Ae^{-k/t}$). The second order rate constants were calculated by dividing the observed rate constants with initial concentration of the TCO.

For UV/VIS-measurements: a solution of the triazine in CH_3CN/H_2O (1:1) (135 μM, 10 mM or 50 mM) and 50 mM solution of the appropriate TCO in CH_3CN/H_2O (1:1) was added to CH_3CN/H_2O (1:1) to a give a final volume of 3 mL and immediately measured on the UV/VIS spectrophotometer. The final concentration of all triazines was either 45 μM or 50 μM using either 5 eq. or 10 eq. of TCO. The decrease in the absorption of the triazine was followed over 5-120 min in intervals of 0.25, 0.5, 1, 2 or 5 min. The time-dependent measurements were performed at the corresponding absorption maxima of the triazine used, which was determined by UV/VIS spectroscopy before the measurement. The measured intensity of the absorption was plotted against time. Fitting the curves with single exponential equation ($y = y_0 + Ae^{-k/t}$) provided the observed rate constants. The second order rate constants were calculated by dividing the observed rate constants with initial concentration of the TCO. All data were processed using Origin or Excel software and are summarized in table S2.

Calculated and found low-resolution masses of the click-products from rate studies.

The following experiments were performed to verify the formation of the corresponding click products during kinetic studies.

30 μL of a 10 mM solution of triazine in CH_3CN/H_2O (1:1) were added to 15 μL of a 25 mM solution of the appropriate TCO in CH_3CN/H_2O (1:1). The mixture was diluted with CH_3CN/H_2O (1:1) to a final volume of 150 μL. The final concentration of all triazines was 1 mM using 1.25 eq. of TCO. The solution was incubated at room temperature for 30 min and then measured by HPLC-MS on a Luna® C18 column (3u, 100A, 100 x 4.6 mm) using a linear gradient of CH_3CN + 0.05% HCOOH (5→95% in 9 min) in H_2O + 0.05% HCOOH at a flow rate of 1.0 mL/min.

During the kinetic measurement we have observed in some cases also the formation of the corresponding oxidized pyridine products together with the dihydropyridine product. These particular cases are indicated in Table S1.

Table S1. Calculated and observed masses of the click products arising from the reaction between 1,2,4-triazines and various TCOs.

**Triazine\TCO**	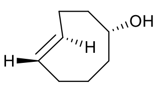 axial		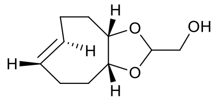 syn:anti 12:1		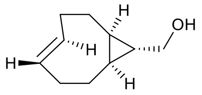 syn	
	calcd.	found	calcd.	found	calcd.	found
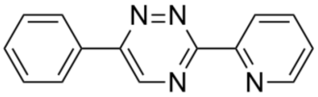 **1**	333.2	333.2 [a]	391.2	391.2[a]	359.2	359.2
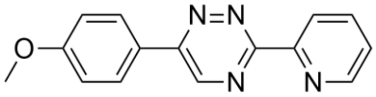 **2**	363.2	363.2 [a]	421.2	421.2[a]	389.2	389.2
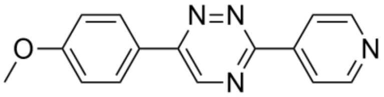 **3**	363.2	363.2 [a]	421.2	421.2	389.2	389.2
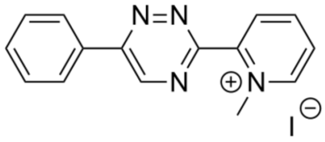 **4**	347.2	347.2 [a]	405.2	405.2	373.2	373.2
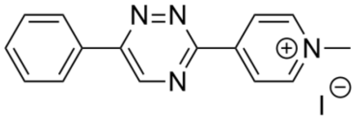 **5**	347.2	347.2 [a]	405.2	405.2	373.2	373.2
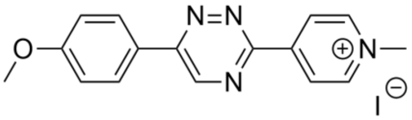 **6**	377.2	377.2 [a]	435.2	435.2	403.2	403.2
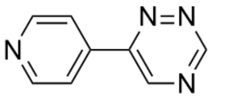 **7**	257.2	257.2 [a]	315.2	315.2	283.2	283.2
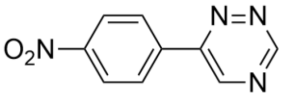 **8**	299.1	299.2[b]	359.2	359.2[a]	327.2	327.2

a) for these compounds the mass of the oxidized form of the click-product was found as well, b) for this compound only the mass of the oxidized form of click-product was found.

Table S2. Second-order rate constants (in M^{-1} s^{-1}) of the reaction between 1,2,4-triazines and various TCOs[a].

Triazine/TCO	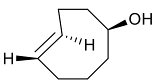 equatorial[b]	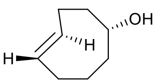 axial[b]	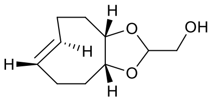 syn:anti 12:1	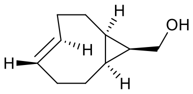 anti	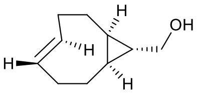 syn
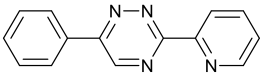 **1**	0.67 ± 0.02	2.5 ± 0.04	0.56 ± 0.008	1.6 ± 0.05	1.9 ± 0.3
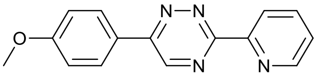 **2**	n.m.	2.1 ± 0.2	0.36 ± 0.003	n.m.	1.2 ± 0.005
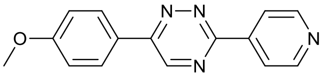 **3**	n.m.	1.7 ± 0.4	0.35 ± 0.02	n.m.	1.4 ± 0.03
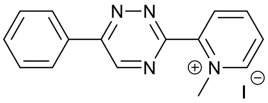 **4**	n.m.	0.66 ± 0.08	0.083 ± 0.013	n.m.	0.30 ± 0.005
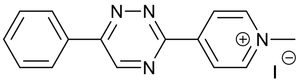 **5**	n.m.	19 ± 2	2.6 ± 0.04	n.m.	9.9 ± 0.3
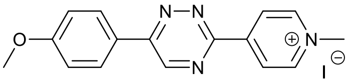 **6**	n.m.	9.1 ± 0.3	1.9 ± 0.06	n.m.	6.4 ± 0.2
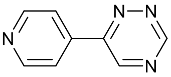 **7**	n.m.	79 ± 5	9.4 ± 0.2	n.m.	20 ± 3
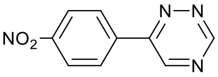 **8**	n.m.	84 ± 7	11 ± 0.5	n.m.	24 ± 0.7

a) all reactions were performed in CH_3CN/H_2O (1:1) at room temperature under pseudo first-order conditions using an excess of the corresponding TCO, b) these rate constants are in M^{-1} s^{-1} $x10^{-2}$, n.m.: not measured

Stability studies of compound 6

The stability studies for triazine **6** were performed in CH_3CN/PBS (1:2) in the absence as well as in the presence of L-cysteine and were monitored by HPLC-MS on a Luna® C18 column (3u, 100A, 100 x 4.6 mm) (solvent A: H_2O + 0.05% HCOOH; solvent B: CH_3CN + 0.05% HCOOH; gradient: 5% B → 95% B in 9 min, then 2 min 95% B and back to 5% B; flow rate: 1.0 mL/min).

For the stability study without L-cysteine, 0.4 mL of a 15 mM solution of triazine **6** in CH_3CN/PBS (1:1) were diluted with PBS to a final volume of 0.6 mL. For the stability study with L-cysteine, 0.2 mL of a 30 mM solution of L-cysteine in PBS were added to 0.4 mL of a 15 mM solution of triazine **6** in CH_3CN/PBS (1:1). The final concentration of all reactants in both studies was 10 mM. In both cases the samples were incubated at 37 °C for 209 h and measured several times on the HPLC-MS (Figure S5 and S6).

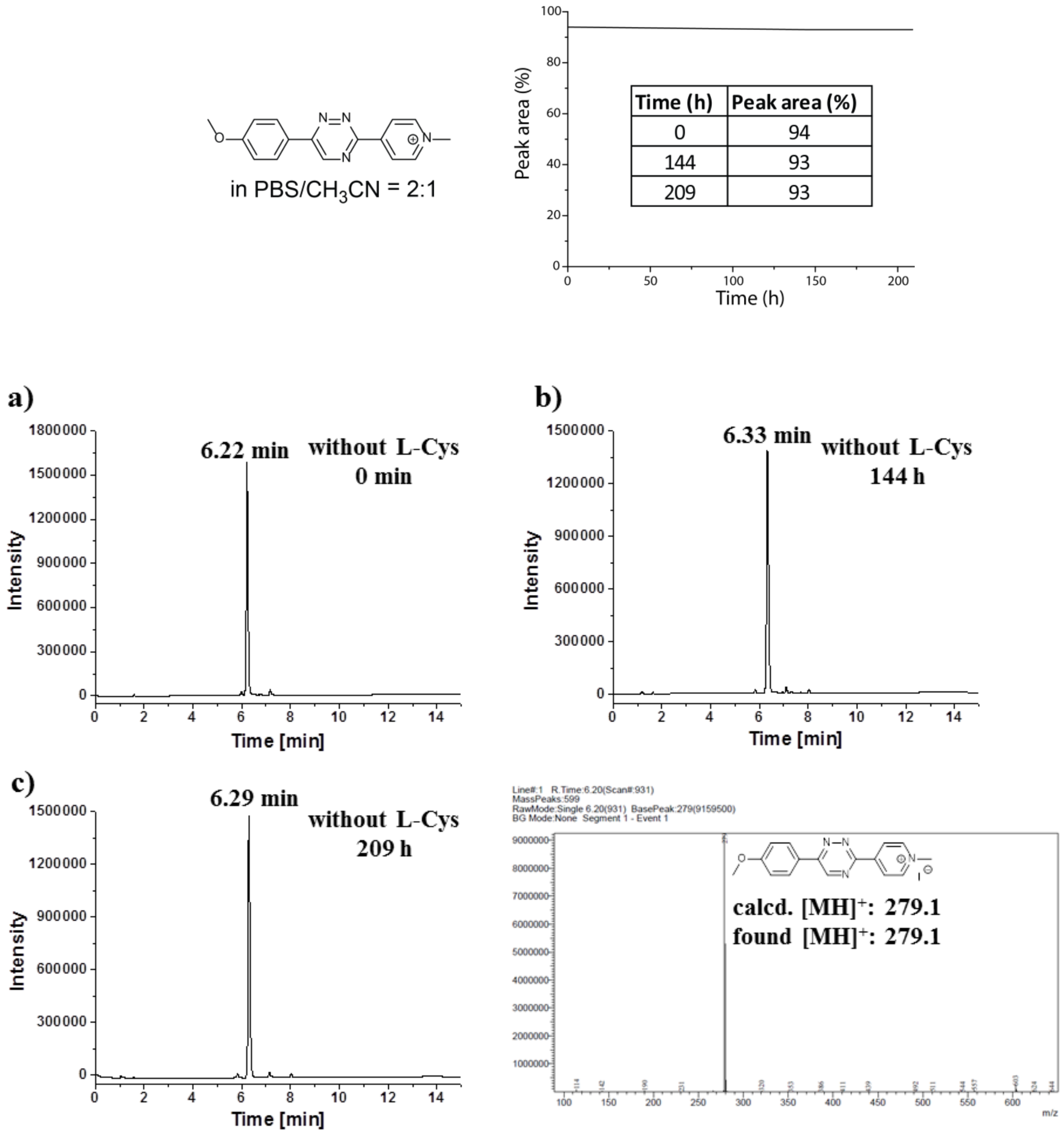

Figure S5. HPLC-MS analysis of **6** in CH_3CN/PBS (1:2) at 37°C after a) 0 min, b) 144 h and c) 209 h. Triazine **6** is stable under these conditions.

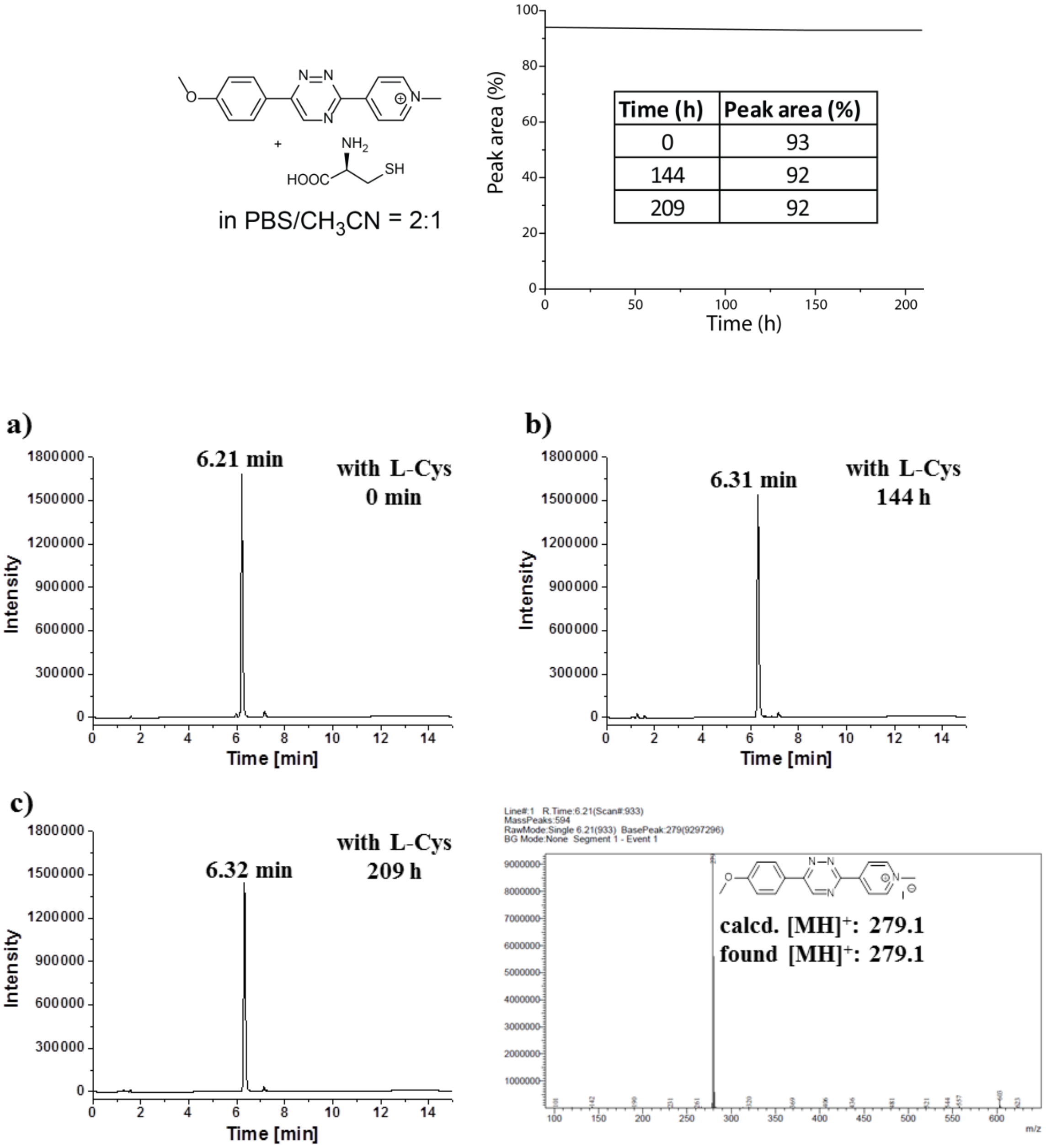

Figure S6. HPLC-MS analysis of **6** in CH_3CN/PBS (1:2) in the presence of L-cysteine at 37°C after a) 0 min, b) 144 h and c) 209 h. Triazine **6** is stable under these conditions.

In addition, the stability of **6** in the presence of L-cysteine was examined in CD_3CN/D_2O (1:1) and followed by NMR spectroscopy. Conditions: 0.4 mL of a 80 mM solution of triazine **6** in CD_3CN/D_2O (1:1) were added to 0.4 mL of a 80 mM solution of L-cysteine in CD_3CN/D_2O (1:1) in order to get 40 mM final concentration of both triazine and L-cysteine. The reaction mixture was incubated at room temperature for 96 h in total. During that time the sample was measured several times by ^{1}H-NMR (Figure S7).

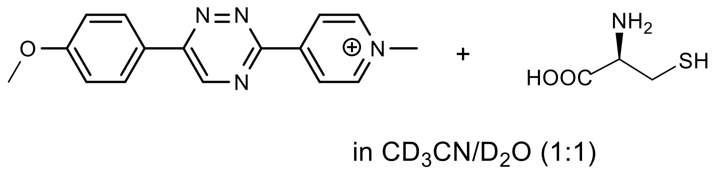

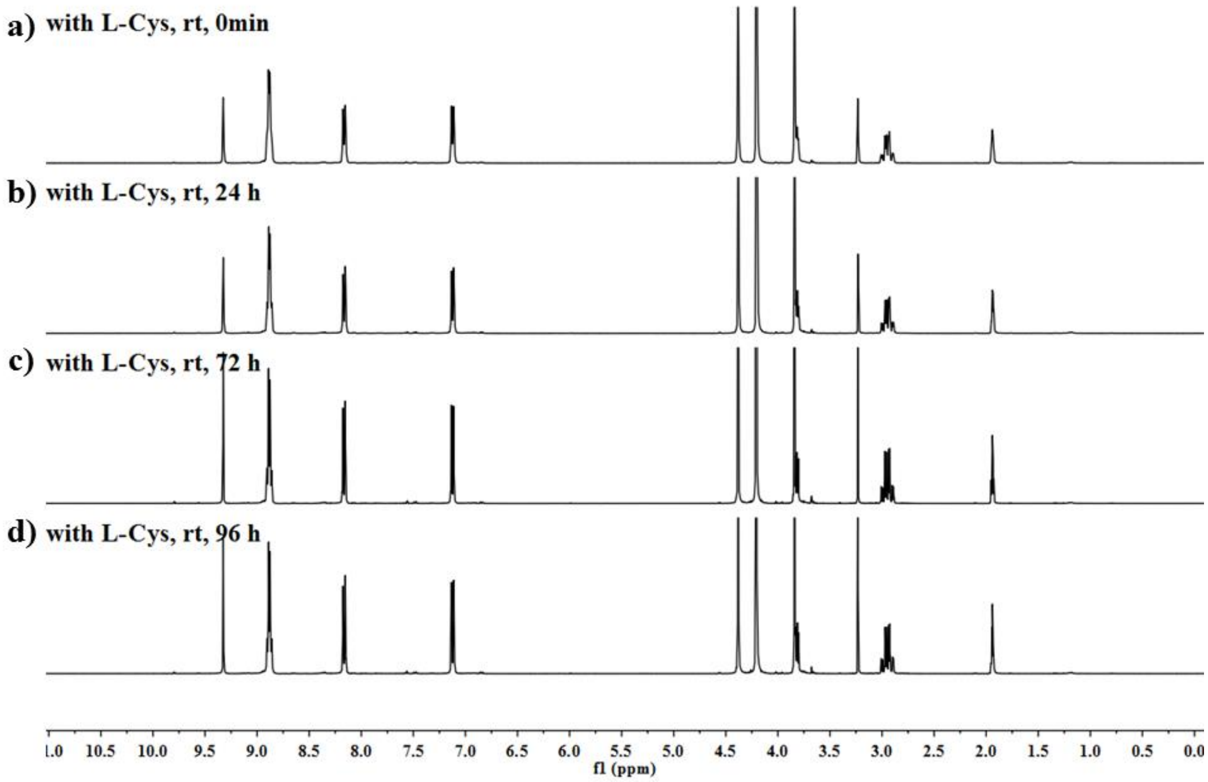

Figure S7. ^{1}H-NMR analysis of **6** in CD_3CN/D_2O (1:1) at room temperature in the presence of L-cysteine after a) 0 min, b) 24 h, c) 72 h and d) 96 h. The measurement further confirmed that **6** is stable in presence of L-cysteine.

Double click-labeling of 12 by two orthogonal metal-free bioconjugations.

A) 50 µL of a 4 mM solution of **12** in CH_3CN/H_2O (1:1) were added to 12 µL of a 25 mM solution of d-TCO in CH_3CN/H_2O (1:1) and diluted with CH_3CN/H_2O (1:1) to a final volume of 100 µL. The final concentration of triazine was 2 mM using 1.5 eq. of d-TCO. The mixture was incubated at room temperature for 1 h and measured by HPLC-MS. After the starting material has disappeared, a 25 mM solution of BCN (2.5 eq. of BCN) in CH_3CN/H_2O (1:1) was added and the mixture was diluted with CH_3CN/H_2O (1:1) to a final concentration of 1.33 mM (based on starting **12**). The mixture was incubated at room temperature for 2.5 h and measured by HPLC-MS (Figure S8).

All HPLC-MS measurements were performed on a Luna® C18 column (3u, 100A, 100 x 4.6 mm) using a linear gradient of CH_3CN + 0.05% HCOOH (5→95% in 9 min) in H_2O + 0.05% HCOOH at a flow rate of 1.0 mL/min.

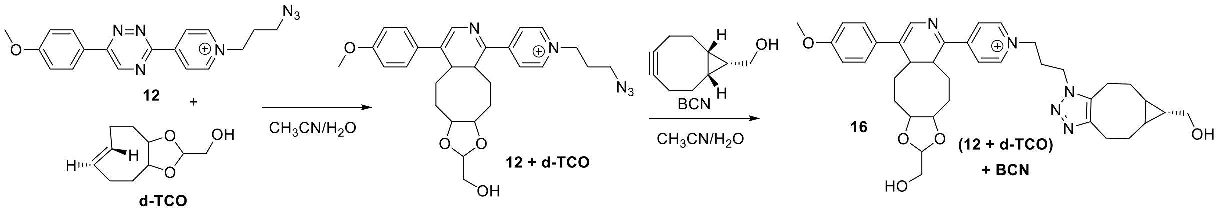

Scheme S1. Sequential double click-labeling of **12** using d-TCO and subsequently BCN.

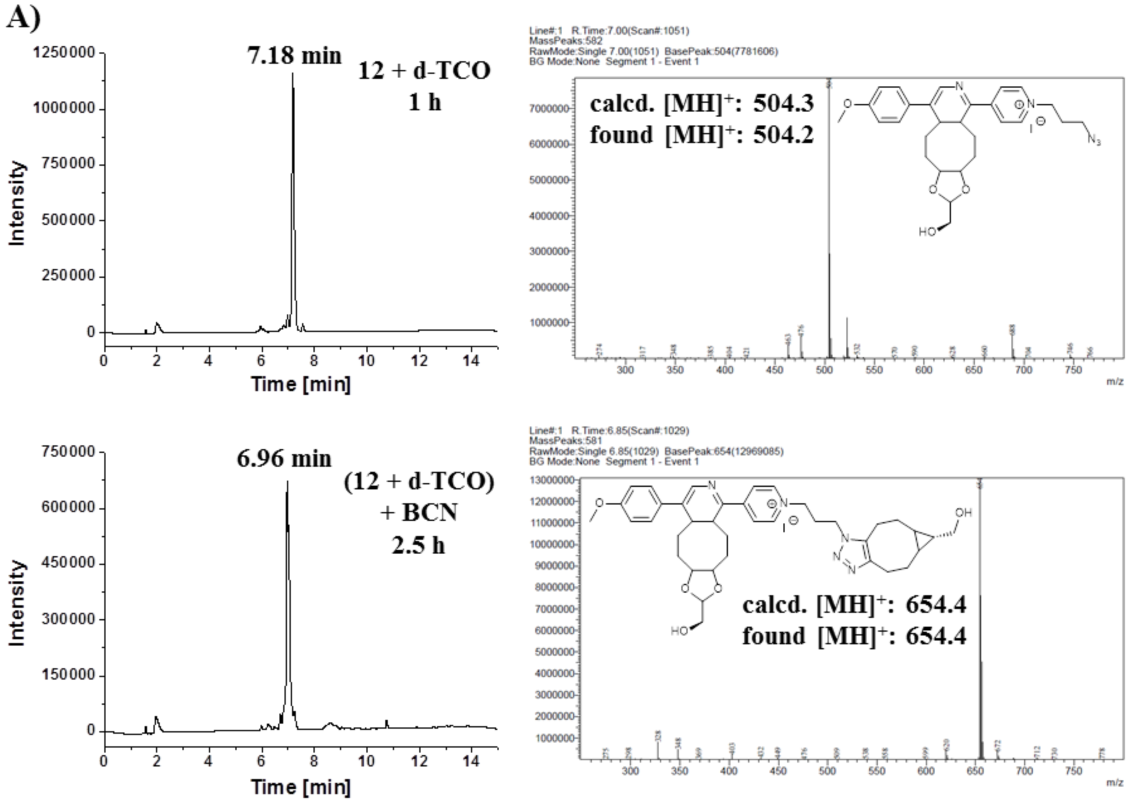

Figure S8. HPLC-MS analysis of sequential double click labeling of **12** incubated first with 1.5 eq. of d-TCO and subsequently with BCN (2.5 eq.) in CH_3CN/H_2O (1:1). The analysis shows that both reactions proceed cleanly and selectively on the respective functional groups.

B) 50 µL of a 4 mM solution of **12** in CH_3CN/H_2O (1:1) were added to 20 µL of a 25 mM solution of BCN in CH_3CN/H_2O (1:1) and diluted with CH_3CN/H_2O (1:1) to a final volume of 100 µL. The final concentration of triazine was 2 mM using 2.5 eq. of BCN. The mixture was incubated at room temperature for 2.5 h and measured by HPLC-MS. After the starting material has disappeared, a 25 mM solution of d-TCO (2.5 eq. of d-TCO) in CH_3CN/H_2O (1:1) was added and the mixture and was diluted with CH_3CN/H_2O (1:1) to a final concentration of 1.33 mM (based on starting **12**). The mixture was incubated at room temperature for 30 min and measured by HPLC-MS (Figure S9).

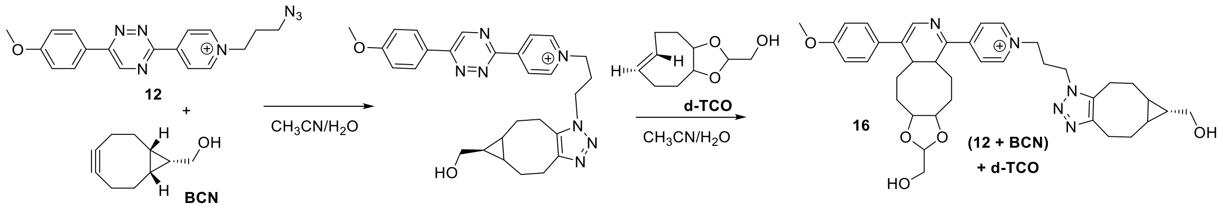

Scheme S2. Sequential double click-labeling of **12** using BCN and subsequently d-TCO.

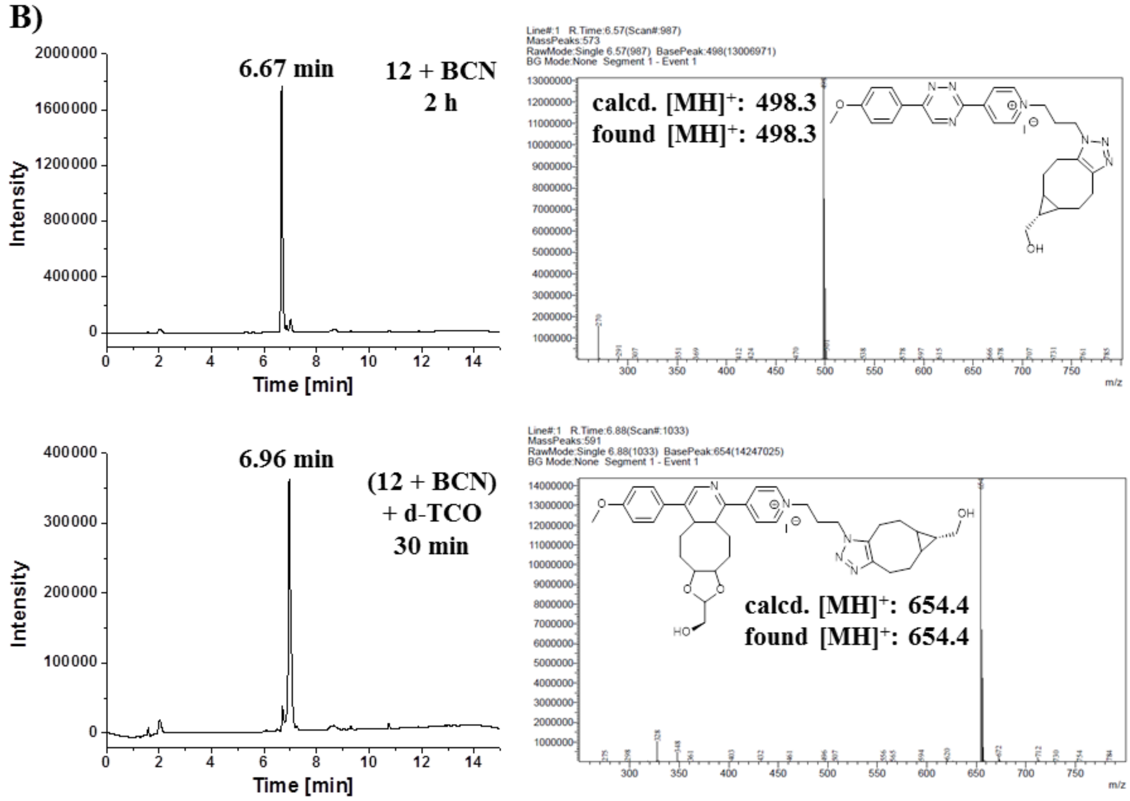

Figure S9. HPLC-MS analysis of sequential double click labeling of **12** incubated first with 2.5 eq. of BCN and subsequently with d-TCO (2.5 eq.) in CH_3CN/H_2O (1:1).

C) 50 µL of a 4 mM solution of **12** in CH_3CN/H_2O (1:1) were added to a mixture of 12 µL of a 25 mM solution of d-TCO in CH_3CN/H_2O (1:1) and 20 µL of a 25 mM solution of BCN in CH_3CN/H_2O (1:1). The mixture was diluted with CH_3CN/H_2O (1:1) to a final volume of 150 µL. The final concentration of triazine was 1.33 mM using 1.5 eq. of d-TCO and 2.5 eq. of BCN. The mixture was incubated at room temperature for 3 h and monitored by HPLC-MS at 30 min, 1.5 h and 3 h (Figure S10).

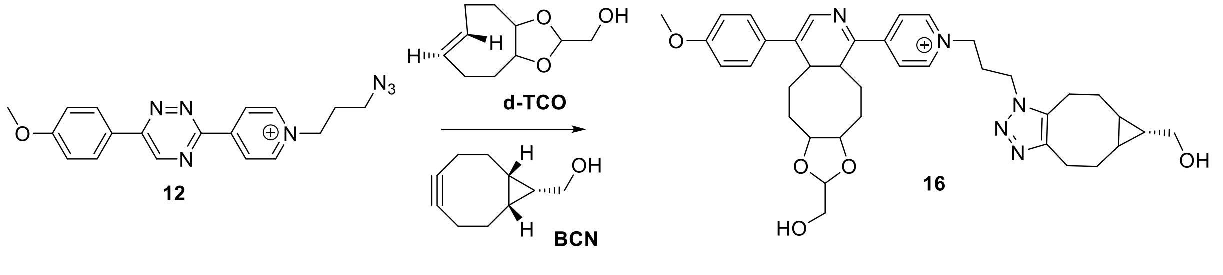

Scheme S3. One-pot double click-labeling of **12** using BCN and d-TCO.

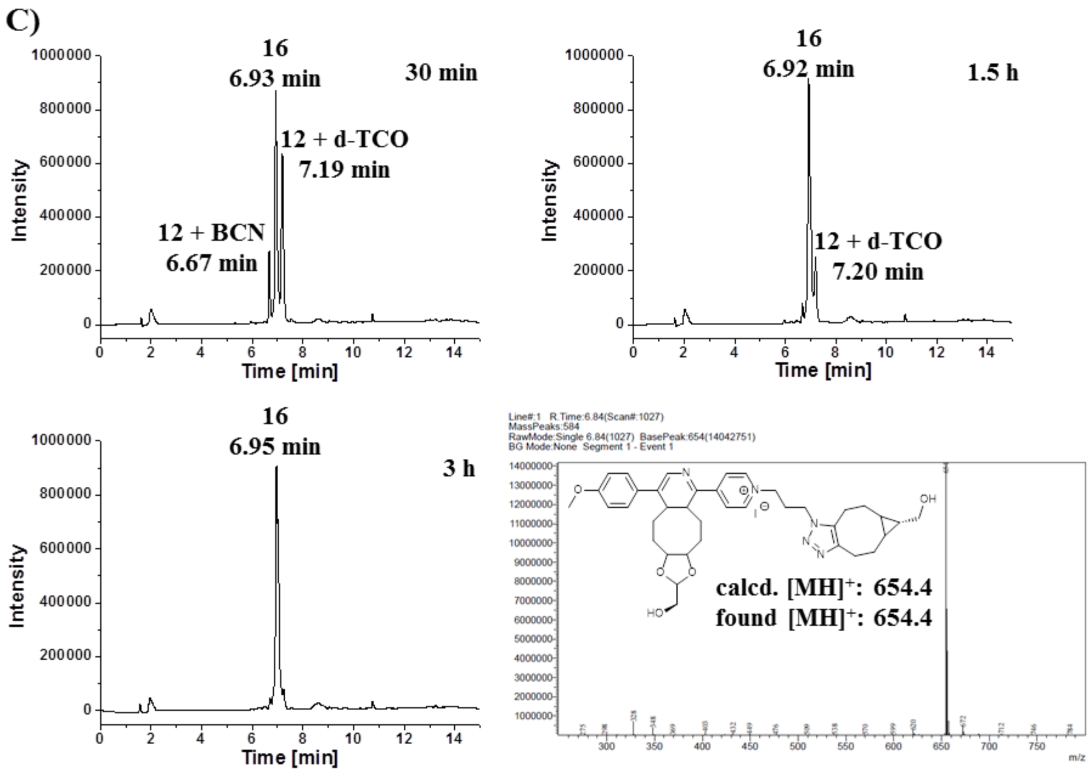

Figure S10. HPLC-MS analysis of the single-step double labeling of **12** with BCN (2.5 eq.) and d-TCO (1.5 eq.) in CH_3CN/H_2O (1:1). The analysis shows that both reactions proceed cleanly and selectively on the respective functional groups.

Synthesis and characterization of the click product 17

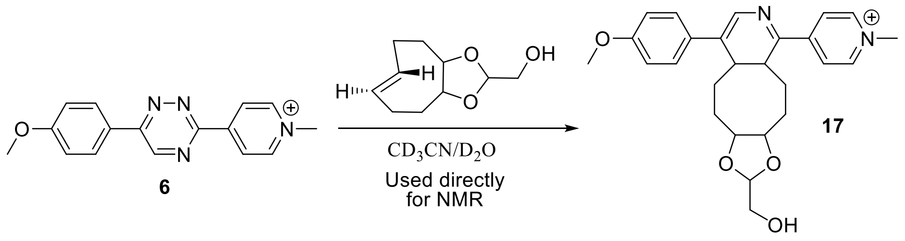

Scheme S4. Reaction scheme for the preparation of **17**.

Solution of d-TCO (5.10 mg, 0.0277 mmol) dissolved in CD_3CN/D_2O (1:1) (500 µL) was added to Triazine **6** (7.5 mg, 0.0185 mmol) dissolved in CD_3CN/D_2O (1:1) (500 µL). The mixture was stirred under argon at room temperature for 1 h. Formation of the click product **17** was verified by HPLC-MS and the crude reaction mixture was directly used for characterization by NMR (Figure S11 and S12).

17 is formed as a mixture of diastereomers (4 in total). Two major diastereomers were assigned based on ROESY experiment. The two minor diastereomers are formed from the anti d-TCO isomer (starting d-TCO was used as an inseparable mixture of syn/anti = 12:1).

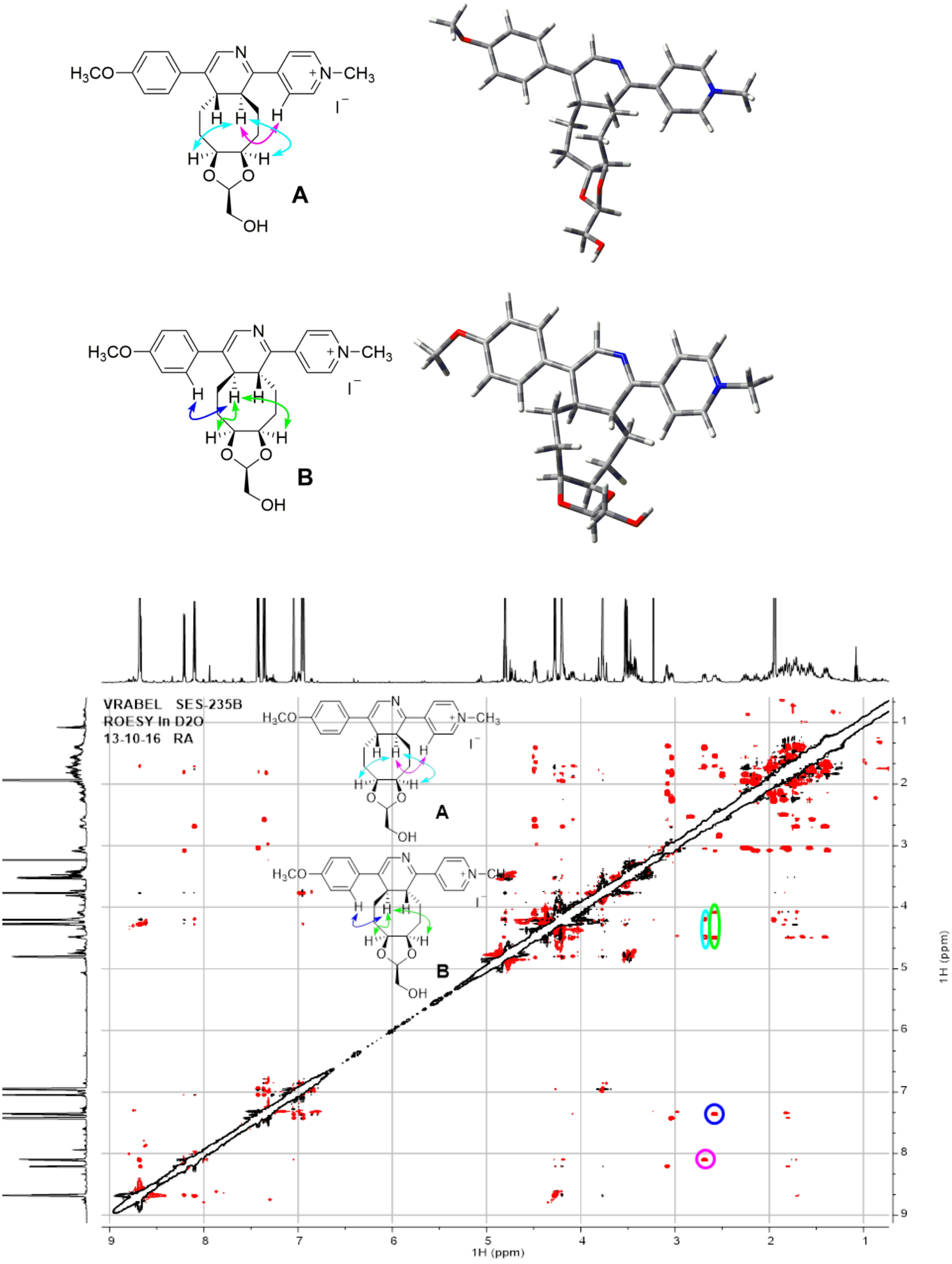

Figure S11. Shown are the key H-H interactions used for the assignment of the two major diastereomers formed upon reaction of **6** with d-TCO.

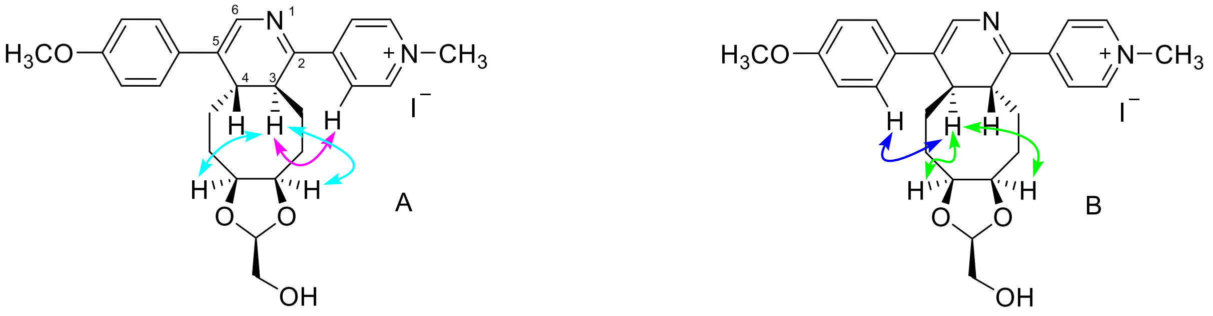

Major NOE signals observed. A mixture of two major diastereoisomers of **17** A:B ~ 5:4.

^{1}H NMR (600.1 MHz, CD_3CN+D_2O (1:1 v/v)): 1.34 – 2.30 (m, 16H, OCHC**H$_2$**C**H$_2$**CH-cyclooct-A,B); 2.57 (m, 1H, H-4-B); 2.69 (ddd, 1H, *J* = 13.8, 6.5, 3.4, H-3-A); 3.04 (ddt, 1H, *J* = 8.7, 6.5, 2.0, H-4-A); 3.09 (m, 1H, H-3-B); 3.50, 3.52 (2 × dd, 2 × 1H, *J* = 12.0, 3.4, C**H$_2$**OH-B); 3.52, 3.54 (2 × dd, 2 × 1H, *J* = 12.2, 3.7, C**H$_2$**OH-A); 3.766 (s, 3H, CH_3O-A); 3.771 (s, 3H, CH_3O-B); 4.09 (m, 1H, OC**H**CH_2CH_2CH-cyclooct-B); 4.19 (m, 1H, OC**H**CH_2CH_2CH-cyclooct-A); 4.27 (s, 3H, CH_3N-B); 4.28 (s, 3H, CH_3N-A); 4.46 – 4.51 (m, 2H, OC**H**CH_2CH_2CH-cyclooct-A,B); 4.80 (t, 1H, *J* = 3.4, OCHO-B); 4.81 (t, 1H, *J* = 3.7, OCHO-A); 6.95 (m, 2H, H-*m*-C_6H_4OMe-A); 6.96 (m, 2H, H-*m*-C_6H_4OMe-B); 7.04 (s, 1H, H-6-B); 7.05 (s, 1H, H-6-A); 7.36 (m, 2H, H-*o*-C_6H_4OMe-B); 7.43 (m, 2H, H-*o*-C_6H_4OMe-A); 8.10 (m, 2H, H-3,5-pyr-A); 8.21 (m, 2H, H-3,5-pyr-B); 8.66 – 8.69 (m, 4H, H-2,6-A,B).

^{13}C NMR (150.9 MHz, CD_3CN+D_2O (1:1 v/v)): 22.68 (OCH**C**H_2CH_2CH-cyclooct-B); 23.49, 23.74 (OCH**C**H_2CH_2CH-cyclooct-A); 24.39 (OCH**C**H_2CH_2CH-cyclooct-B); 29.66 (OCHCH_2**C**H_2CH-cyclooct-A); 30.73, 33.64 (OCHCH_2**C**H_2CH-cyclooct-B); 34.50 (OCHCH_2**C**H_2CH-cyclooct-A); 37.05 (CH-4-A); 37.93 (CH-3-B); 38.20 (CH-4-B); 39.28 (CH-3-A); 48.66 (CH_3N-B); 48.72 (CH_3N-A); 56.16 (CH_3O-A); 56.17 (CH_3O-B); 62.57 (CH_2O-B); 62.89 (CH_2O-A);79.77 (O**C**HCH_2CH_2CH-cyclooct-A); 80.14 (O**C**HCH_2CH_2CH-cyclooct-B); 80.37 (O**C**HCH_2CH_2CH-cyclooct-A); 80.62 (O**C**HCH_2CH_2CH-cyclooct-B); 101.44 (OCHO-B); 101.45 (OCHO-A); 115.24 (CH-*m*-C_6H_4OMe-A); 115.28 (CH-*m*-C_6H_4OMe-B); 125.97 (CH-3,5-pyr-B); 126.07 (CH-3,5-pyr-A); 128.82 (CH-*o*-C_6H_4OMe-A); 128.87 (CH-*o*-C_6H_4OMe-B); 129.77 (CH-6-B); 129.89 (CH-6-A); 130.27 (C-*i*-C_6H_4OMe-B); 130.50 (C-*i*-C_6H_4OMe-A); 133.34 (C-5-B); 133.64 (C-5-A); 146.22 (CH-2,6-pyr-B); 146.32 (CH-2,6-pyr-A); 153.98 (C-4-pyr-A); 154.10 (C-4-pyr-B); 160.72 (C-*p*-C_6H_4OMe-A); 160.77 (C-*p*-C_6H_4OMe-B); 161.88 (C-2-B); 161.94 (C-2-A).

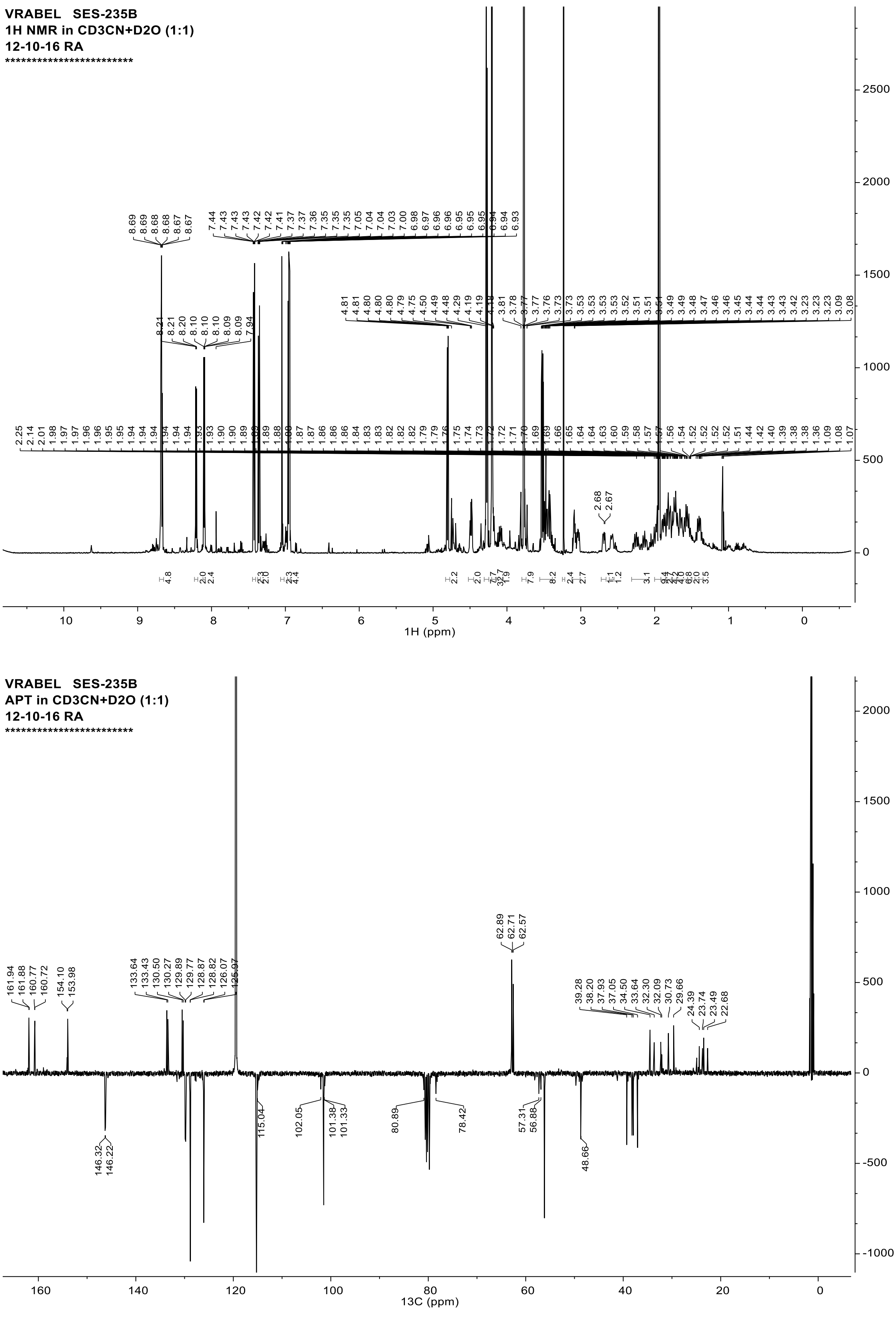

Figure S12. ^{1}H and ^{13}C NMR spectra of the diastereomeric mixture of **17**.

Formation of the oxidized product 17ox during preparative TLC

Our attempt to purify the click product **17** by preparative TLC (eluting with CH_3CN/H_2O (4:1) and washing the silica with CH_3CN/H_2O (4:1) + 0.5% HCOOH led to isolation of the corresponding oxidation click product **17ox**. The identity of the oxidized product was confirmed by HPLC-MS (Figure S13) and NMR.

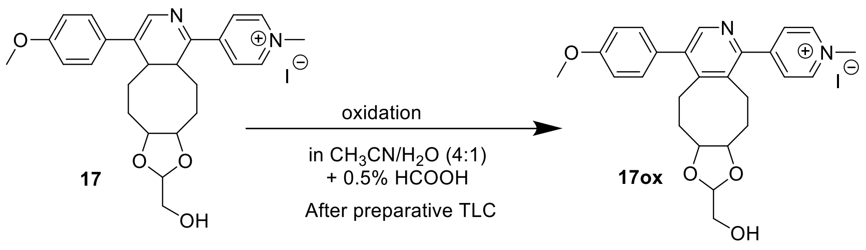

Scheme S5. Formation of **17ox** in CH_3CN/H_2O (4:1) + 0.5% HCOOH during purification by preparative TLC.

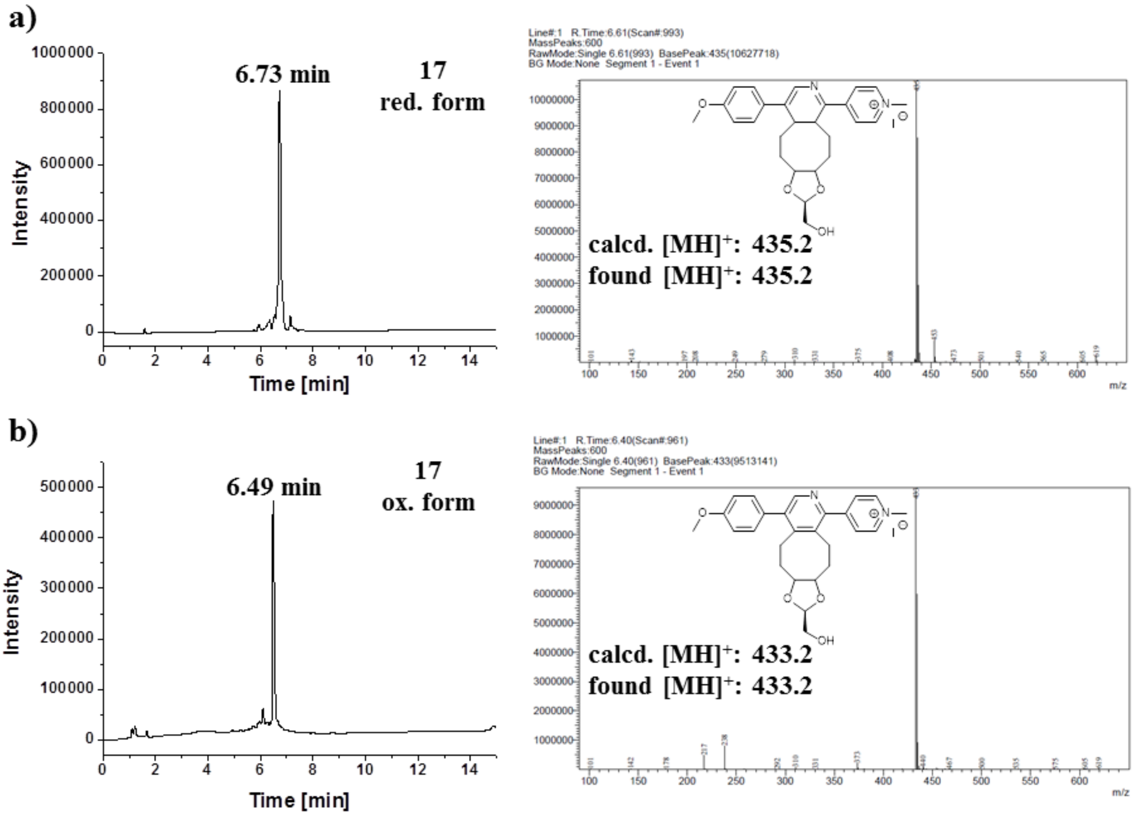

Figure S13. HPLC-MS analysis of a) the crude reaction mixture of compound **17** and b) the oxidized form **17ox**. Conditions: Luna® C18 column (3u, 100A, 100 x 4.6 mm) using a linear gradient of CH_3CN + 0.05% HCOOH (5→95% in 9 min) in H_2O + 0.05% HCOOH at a flow rate of 1.0 mL/min

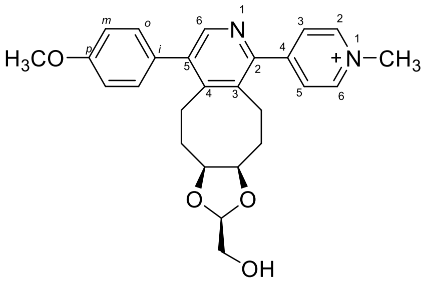

Major diastereomer:

^{1}H NMR (500.0 MHz, DMSO-d_6): 1.91 – 2.12 (m, 4H, $OCHCH_2CH_2C$-cyclooct.); 2.55 – 2.62, 2.78 – 2.87 (2 × m, 2 × 2H, $OCHCH_2CH_2C$-cyclooct.); 3.39 (d, 2H, J = 4.0, CH_2OH); 3.82 (s, 3H, CH_3O); 4.17 – 4.29 (m, 2H, $OCHCH_2CH_2C$-cyclooct.); 4.45 (s, 3H, CH_3N); 4.75 (t, 1H, J = 4.0, OCHO); 7.10 (m, 2H, H-*m*-C_6H_4OMe); 7.29 (m, 2H, H-*o*-C_6H_4OMe); 8.18 (d, 2H, J = 5.3, H-3,5-pyr); 8.42 (s, 1H, H-6); 9.16 (d, 1H, J = 5.3, H-2,6-pyr).

^{13}C NMR (125.7 MHz, DMSO-d_6): 23.47, 23.71 ($OCHCH_2CH_2C$-cyclooct.); 30.47 ($OCHCH_2CH_2C$-cyclooct.); 47.77 (CH_3N); 55.45 (CH_3O); 62.60 (CH_2OH); 78.20 (br, $OCHCH_2CH_2C$-cyclooct.); 102.55 (OCHO); 114.34 (CH-*m*-C_6H_4OMe); 127.68 (CH-3,5-pyr); 129.53 (C-*i*-C_6H_4OMe); 130.58 (CH-*o*-C_6H_4OMe); 136.07 (C-3); 138.03 (C-5); 145.75 (CH-2,6-pyr); 147.92 (CH-6); 150.23 (C-4); 151.72 (C-2); 155.70 (C-4-pyr); 159.27 (C-*p*-C_6H_4OMe).

Stability of the click product 17

The stability studies of the click product **17** were performed in CH_3CN/H_2O (1:1) at room temperature under exposure to air and were monitored by HPLC-MS on a Luna® C18 column (3u, 100A, 100 x 4.6 mm) using a linear gradient of CH_3CN + 0.05% HCOOH (5→95% in 9 min) in H_2O + 0.05% HCOOH at a flow rate of 1.0 mL/min.

0.5 mL of a 10 mM solution of **6** in CH_3CN/H_2O (1:1) were added to 0.3 mL of a 25 mM solution of d-TCO in CH_3CN/H_2O (1:1). The mixture was diluted with CH_3CN/H_2O (1:1) to a final volume of 1 mL in order to get 5 mM final concentration of **6** using 1.5 eq of d-TCO. The solution was stirred at room temperature until starting material has disappeared (5 h, monitored by HPLC-MS, indicated time 0 min in Figure S13). For stability study, the click-product **17** was stirred at room temperature for 97 h in total under exposure to air. During that time the reaction mixture was measured several times by HPLC-MS (Figure S14).

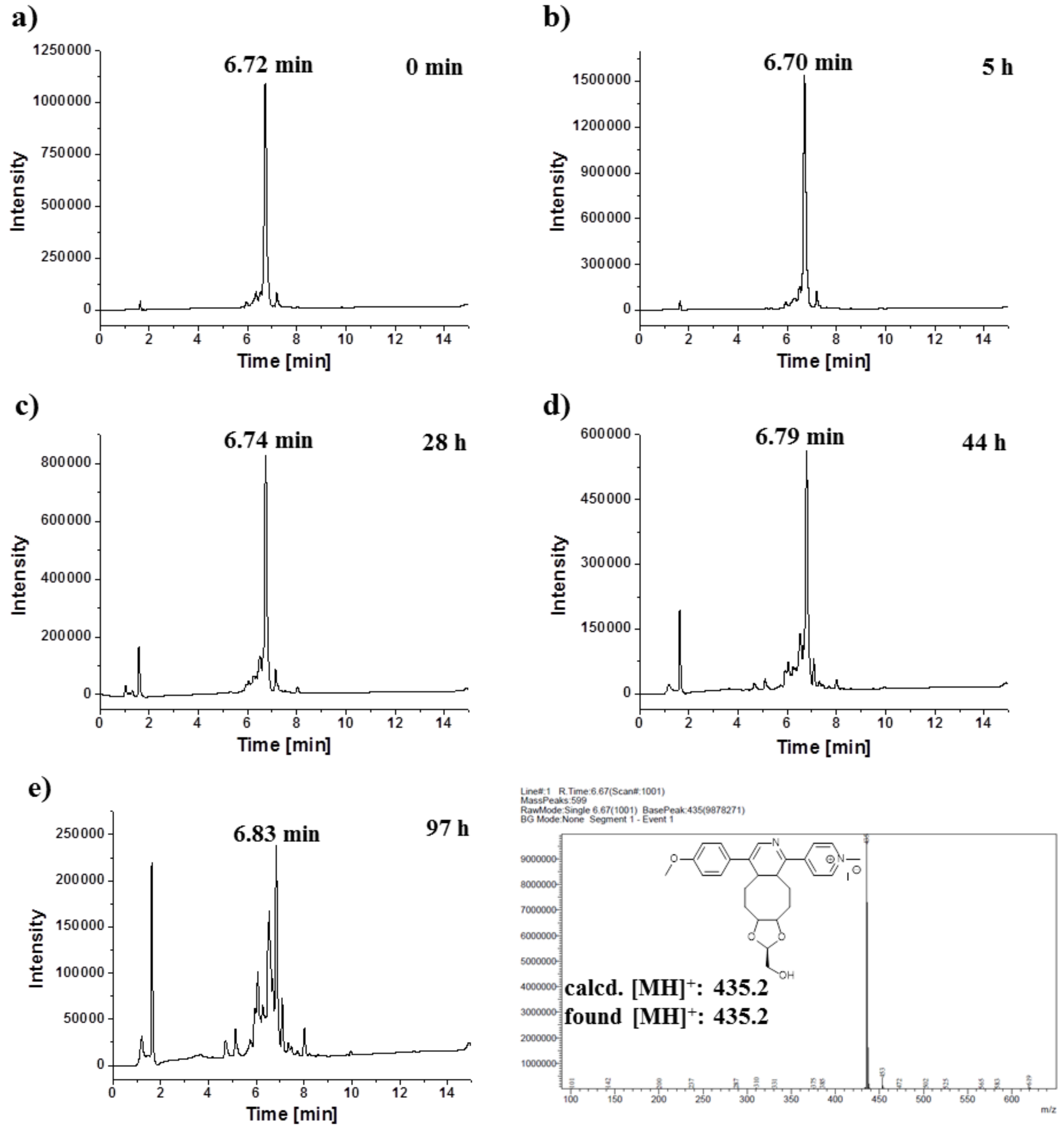

Figure S14. HPLC-MS analysis of **17** in CH_3CN/H_2O (1:1) at room temperature under exposure to air after a) 0 min, b) 5 h, c) 28 h, d) 44 h and e) 97 h.

Photophysical properties of the click product 17

A freshly prepared solution of **17** was used in the following experiments. Conditions: To 50 µL of a solution of **6** in CH_3CN/H_2O (1:1) (stock: 50 mM) were added 50 µL of d-TCO solution in CH_3CN/H_2O (1:1) (stock: 100 mM, 2 equiv.) giving a 25 mM final concentration of **6** and **17** respectively. The reaction mixture was incubated at room temperature in the dark for 0.5 h and then analyzed by HPLC-MS to verify the formation of the click product (Figure S15). This solution was further diluted in different solvents and used as such for absorbance measurements the quantum yield determination measurements (Figure S16-S18).

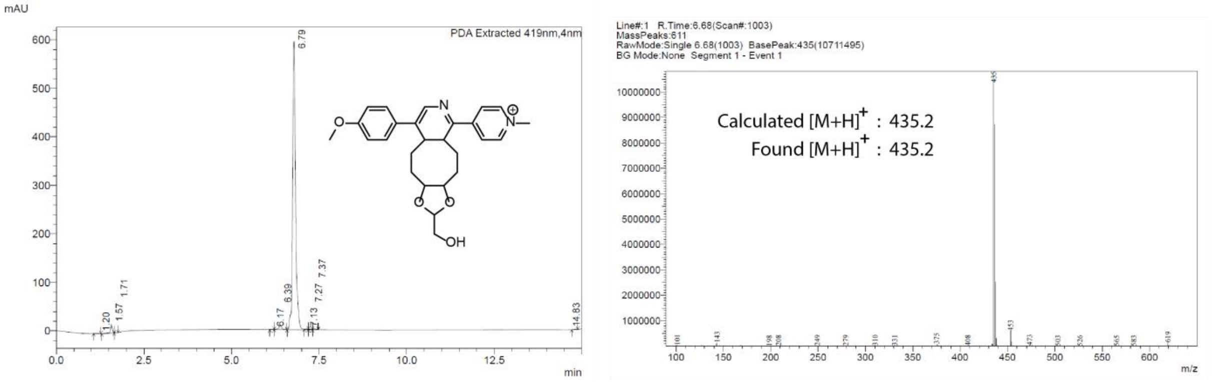

Figure S15. HPLC chromatogram of the reaction mixture between **6** and d-TCO showing the formation of **17** after 0.5 h and the corresponding MS spectra. (solvent A: H_2O + 0.05% HCOOH; solvent B: CH_3CN + 0.05% HCOOH; gradient: 5% B → 95% B in 9 min, then 2 min 95% B and back to 5% B). The product eluates at 6.8 min (Luna® C18 column, 3u, 100A, 100 x 4.6 mm, 1 mL/min flow rate).

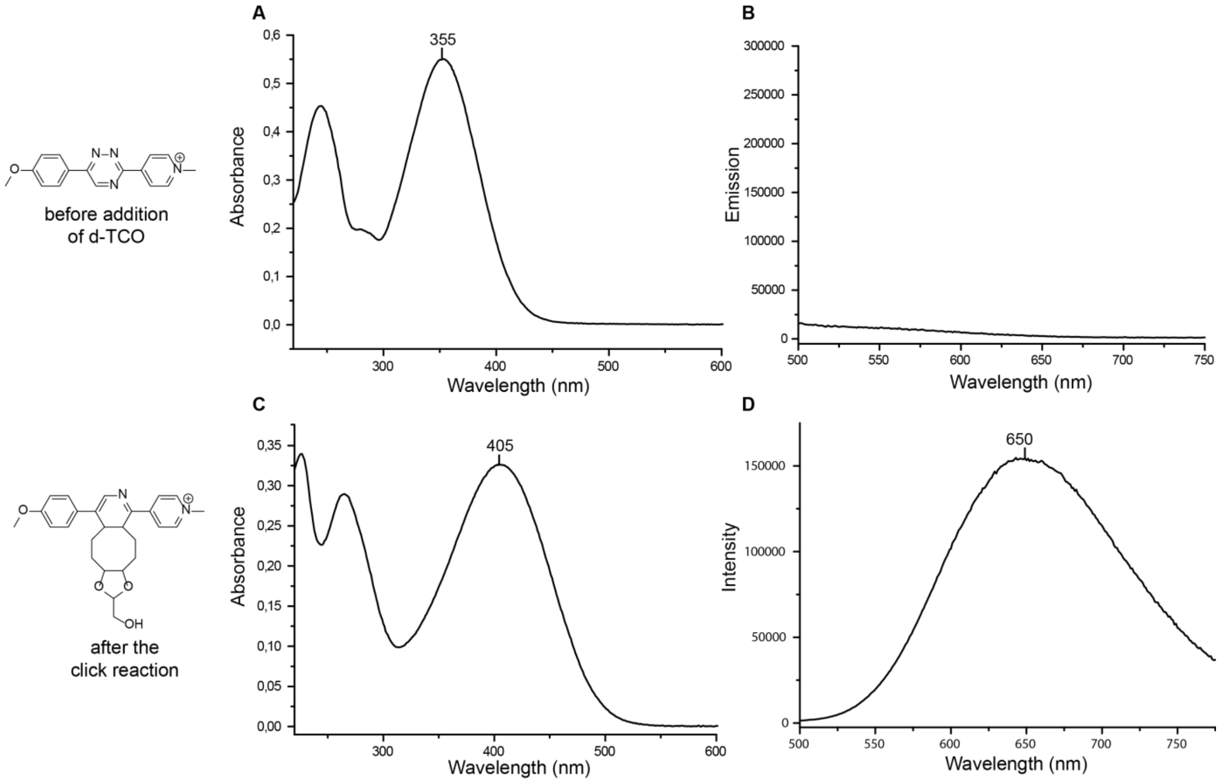

Figure S16. Absorption and emission spectra of **6** (A and B respectively) and of the click product **17** (C and D respectively). Measured in 1x PBS buffer (pH 7.4) at room temperature (25 µM for absorbance and 2.5 µM for fluorescence).

Quantum yield of the click product **17** in different solvents was measured at 22°C in 1 cm quartz cuvette using 5µM final concentration of **17**. The experiment was performed on FluoroMax 4 spectrofluorometer (Jobin Yvon, Horiba) equipped with a 450 W xenon lamp using Rhodamine 6G (solution in EtOH) as reference (ϕ_{QS} = 0.94). The settings were as follows: Excitation wavelength 405 nm, slit 5.0 nm; Emission 500 – 750 nm, increment 1.0 nm, slit 5.0 nm and data algebra formula S1c/R1c. The fluorescence quantum yields were calculated using the following equation:

$$\phi_{sample} = \phi_{ref} \frac{F_{sample}}{F_{ref}} \times \frac{(1\text{-}10^{-abs})_{ref}}{(1\text{-}10^{-abs})_{sample}} \times \frac{n^2_{sample}}{n^2_{ref}}$$

Where:

$\boldsymbol{\phi_{ref}}$ is 0,94 (Quantum yield of Rhodamine 6G in EtOH)[13]

F are the integrated intensities (areas) of standard and the sample fluorescence spectra (integrals calculated using OriginPro software)

abs is the absorbance of standard and sample at the excitation wavelength (405 nm)

n are the refractive indices for standard (EtOH: 1.3616) and the sample solution (CH_3CN: 1.3441; PBS: 1.3284; MeOH: 1.327; THF: 1.4072; $CHCl_3$: 1.4441)

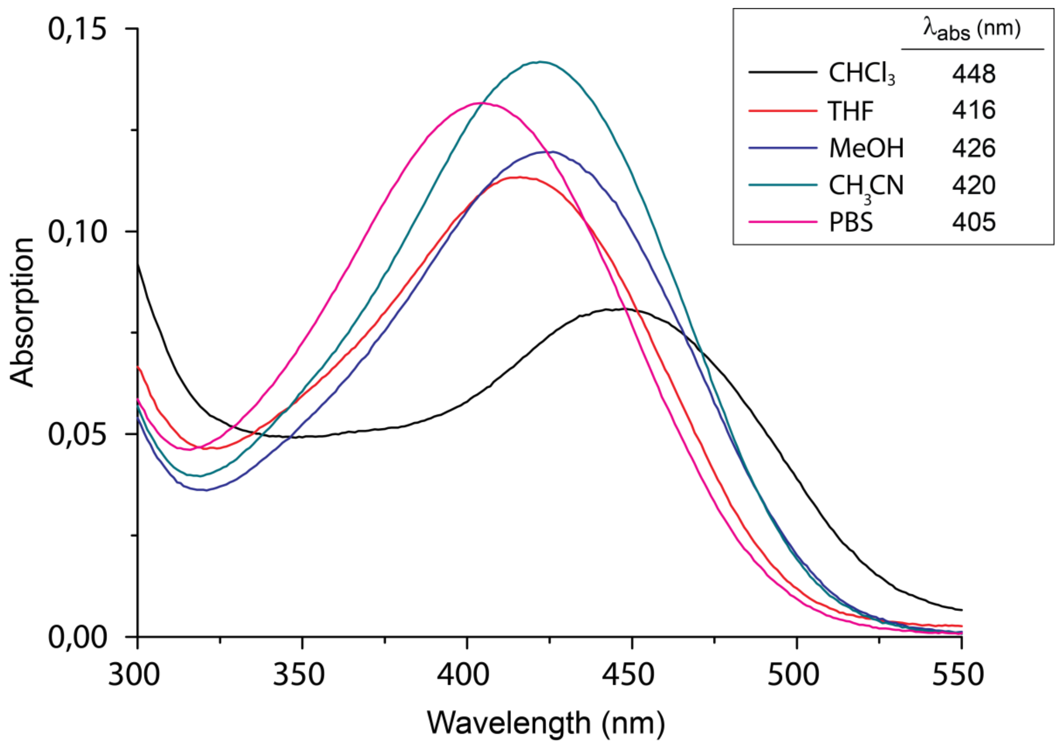

Figure S17. Absorption spectra of the click product **17** in different solvents.

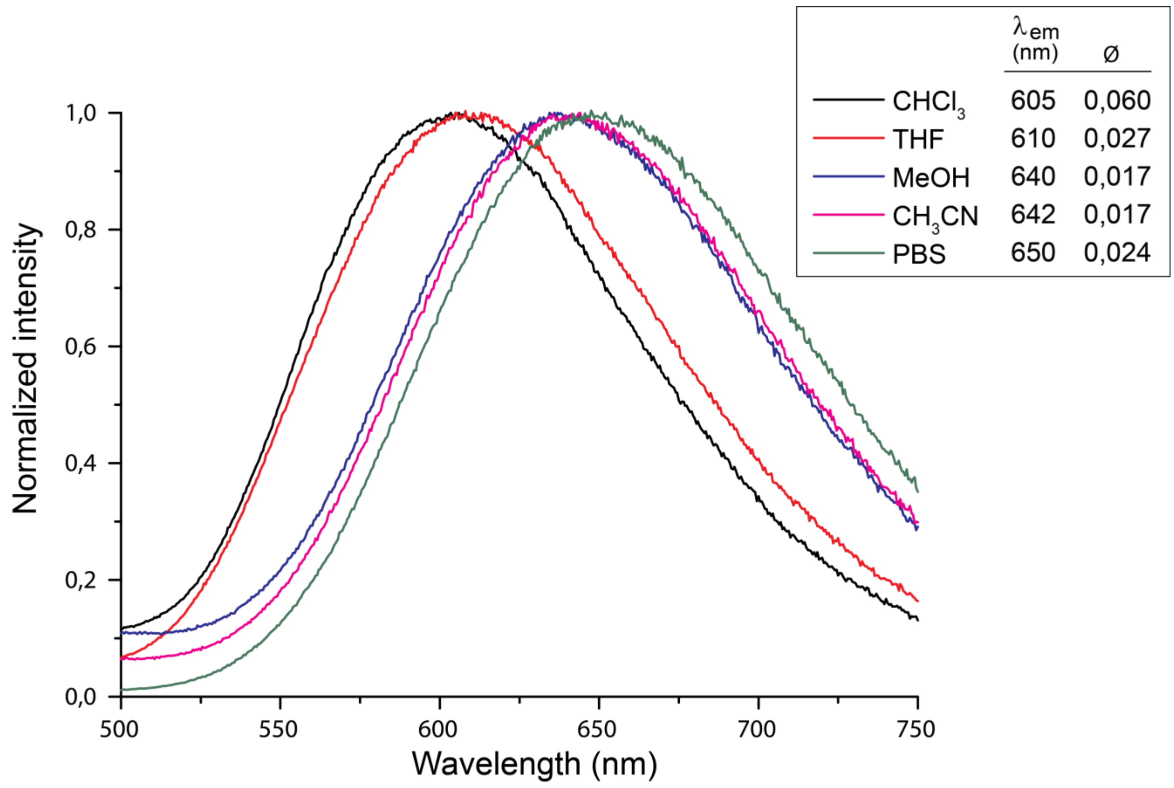

Figure S18. Emission spectra and quantum yield of the click product **17** in different solvents.

Cell labeling experiments

U2OS cells were maintained in high glucose DMEM (*Sigma*) supplemented with 10% FBS (*Biosera*) and antibiotics at 37 °C/5% CO_2. One day before experiment cells were seeded at density $0.3x10^6$ at the glass bottom dishes (*SPL Life Sciences* 3.5 cm diameter). Triazine **6** was dissolved in DMSO/H_2O = 1/1 (5 mM) and further diluted before experiments. d-TCO stock solution was in DMSO (50 mM).

Images were acquired on *Zeiss* LSM 780 or Leica TCS SP5 confocal scanning microscopes and raw pictures were processed by *FIJI* software.[14]

Excitation: 405nm, emission window: 525–648 nm for click products). The nucleus was stained with DRAQ5 (excitation: 633 nm, emission window: 667–748 nm).

Mitochondria labeling in live U2OS cells

U2OS Cells were incubated with 5 µM (final) concentration of **6** in complete media for 3 h/37 °C. Cells were washed once with the media, and incubated for further 30 min in complete DMEM medium without phenol red containing 500 nM DRAQ5 dye (*Thermo Fisher*). The cells were then incubated with d-TCO (25 µM final concentration) for 15 min at 37 °C prior to imaging (Figure S19).

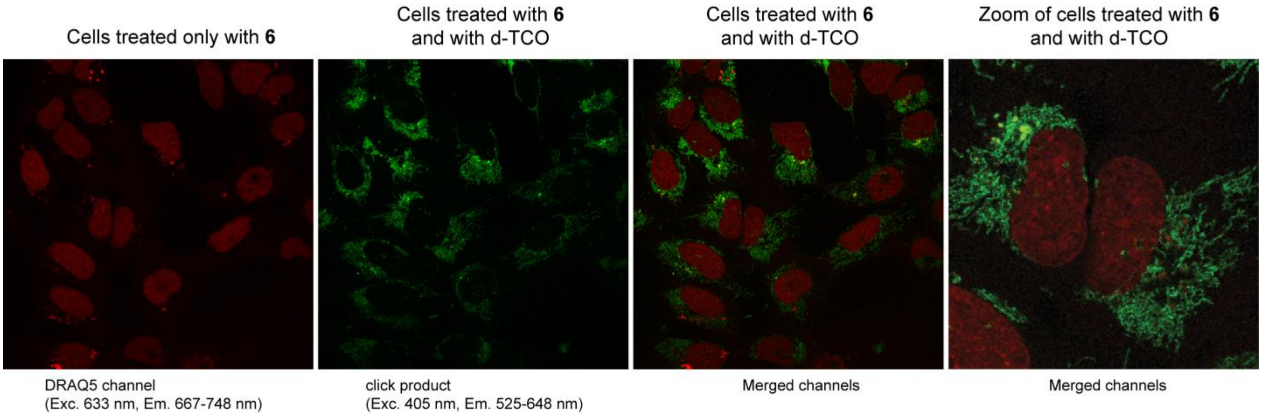

Figure S19. U2OS cell labeling experiment using triazine **6** and d-TCO**.**

Colocalization experiment of 6 + dTCO and Mitotracker green

U2OS Cells were incubated with 25 µM (final) concentration of **6** in complete media for 2 h/37 °C. Cells were washed once with the media, and incubated for 15 min with d-TCO (25 µM final concentration). Then a 100 nM Mitotracker green (*Thermo Fisher*) and 500 nM DRAQ5 (*Thermo Fisher*) solutions were added. The cells were incubated for 10 min at 37 °C, washed several times with DMEM medium, and mounted to the confocal microscope for imaging (Figure S20). The images were acquired sequentially using the following filter set-up: Click product: excitation 405 nm, emission 560-666 nm;   Mitotracker green: excitation

496 nm, emission 505-588 nm; DRAQ5 channel: excitation 633 nm, emission 667-748 nm. Laser intensity was set up to 10%.

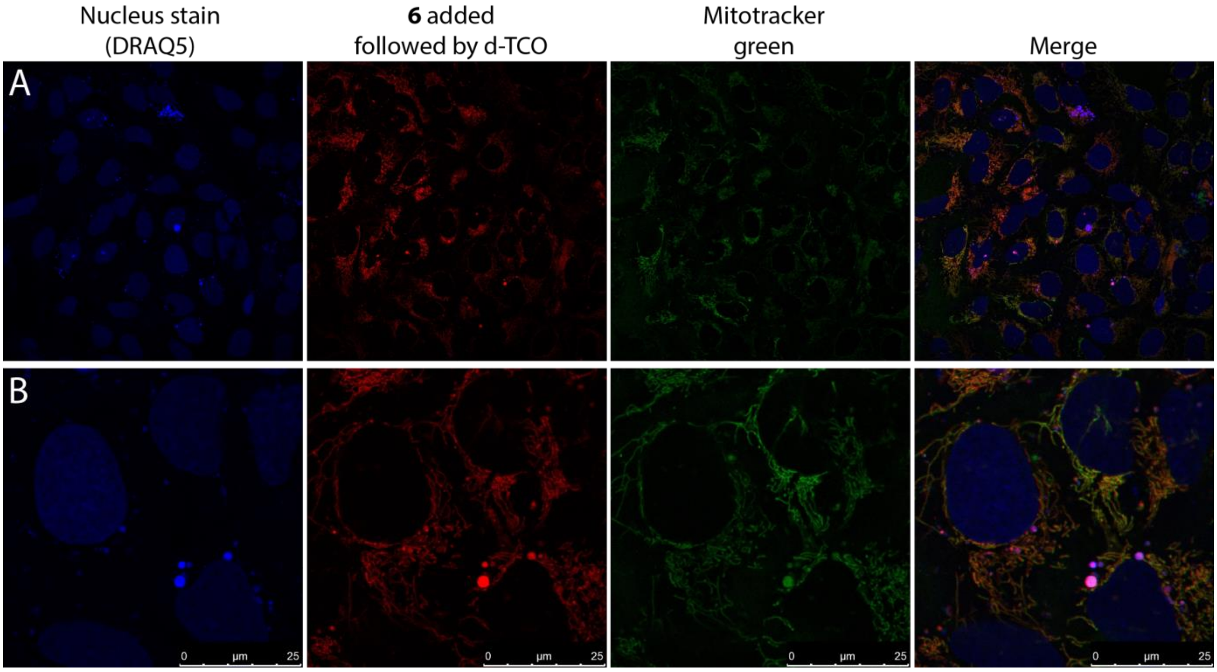

Figure S20. Colocalization experiment using U2OS cells treated with triazine **6** (25 µM), d-TCO (25 µM) and with mitotracker green (100 nM). B) is a zoom of pictures A. The experiment confirms that **6** is targeted to mitochondria where it can be labeled with d-TCO. The pictures were processed using LAS AS Lite and FIJI software. Filter set up: Click product: ex. 405 nm, em. 560-666 nm; Mitotracker green: ex. 496 nm, em. 505-588 nm. DRAQ5 channel: ex. 633 nm, em. 667-748 nm.

Preparation of concanavalin A d-TCO conjugate (ConA-dTCO)

2.5 mg of Concanavalin A (*Sigma* #C2010) were dissolved in 1 ml of 150 mM NaCl, 50 mM HEPES pH 8.3 (to obtain a 2.5 mg/ml solution). 250 µL (0.625 mg) of this solution were combined with 3.6 µL of 100 mM d-TCO NHS active ester (10x molar excess dissolved in dry DMSO). The reaction mixture was incubated at room temperature for one hour with constant shaking. After one hour precipitated material was spun at 25000 rpm for 10 min. Clean supernatant was split and 2x 130 µL was loaded onto two Zeba desalting columns preconditioned with 150 mM NaCl, 50 mM TRIS pH 7.4 (in total 2 columns were used).
Note: The d-TCO active ester partially precipitated when added to ConA but after one hour the solution became clear again.

ConA-dTCO Experiment 1 on live cells

Live U2OS cells were incubated for 10 min with ConA-dTCO (100x diluted) in Dulbeccos PBS (DPBS, with Ca^{2+} and Mg^{2+}). After washing with DPBS cells were incubated with 50 µM **6** in DPBS with 500 nM DRAQ5 for 15 min. Pictures of living cells were taken at

405 nm excitation. Emission was collected at 525-648 nm. DRAQ channel: Exc. 633 nm, Em. 667-748 nm (Figure S21).

Neg. control incubated with ConA at room temp, No triazine **6**

Live cells with ConA-dTCO after addition of **6**

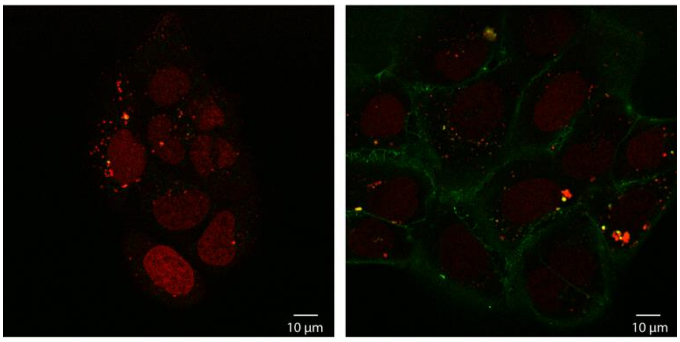

Merged channels

Merged channels

Figure S21. U2OS live cell labeling experiment using ConA-dTCO conjugate and triazine **6**. Only weak signal has been observed when the experiment was performed on live cells.

ConA-dTCO Experiment 2

Live U2OS cells were incubated for 10 min with ConA-dTCO (100x diluted) in Dulbeccos PBS (DPBS, with Ca^{2+} and Mg^{2+}). After fixation with 4% formaldehyde for 10 min and permeabilization with 0.1% Triton X100 in PBS for another 10 min cells were washed with DPBS and incubated with 50 µM **6** in PBST and with 500 nM DRAQ5 for 15 min. Pictures of fixed cells were taken at 405 nm excitation. Emission was collected at 525-648 nm. DRAQ channel: Exc. 633 nm, Em. 667-748 nm (Figure S22).

Negative control incubated with **6**

Incubated with ConA-dTCO, fixed, permeabilized and incubated with **6**

Incubated with ConA-dTCO, fixed, permeabilized and incubated with **6**

Crop picture, ConA-dTCO, fixed, permeabilized and incubated with **6**

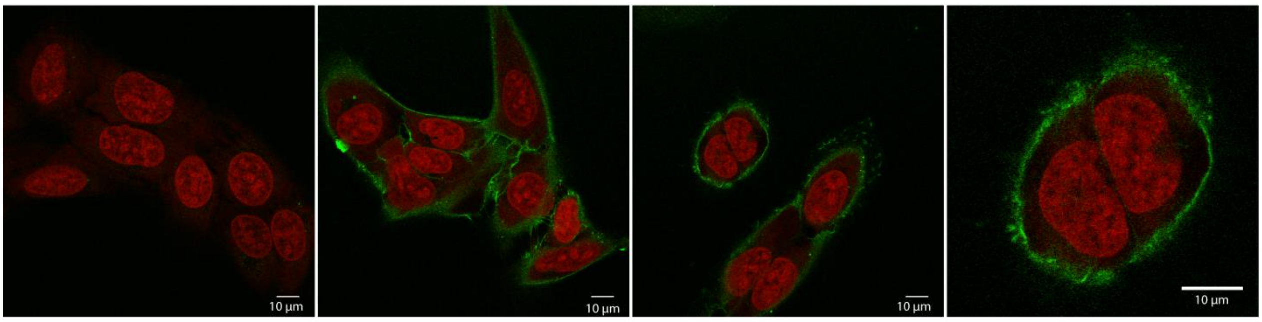

Merged channels

Merged channels

Merged channels

Merged channels

Figure S22. U2OS cell membrane labeling experiment using ConA-dTCO conjugate and triazine **6**.

ConA-dTCO Experiment 3

U2OS cells were fixed with 4% formaldehyde for 10 min. After permeabilization with 0.1% Triton X100 in PBS cells and 3x washing with PBS 0.05% TWEEN20 (PBST) cells were incubated for 10 min with ConA-dTCO (100x diluted) in PBST. After washing 3x with PBST cells were incubated with 50 µM **6** in PBST containing 500 nM DRAQ5 for 15 min. Pictures

of fixed cells were taken at 405 nm or 458 nm excitation. Emission was collected at 525-648 nm. DRAQ5 channel: Exc. 633 nm, Em. 667-748 nm (Figure S23).

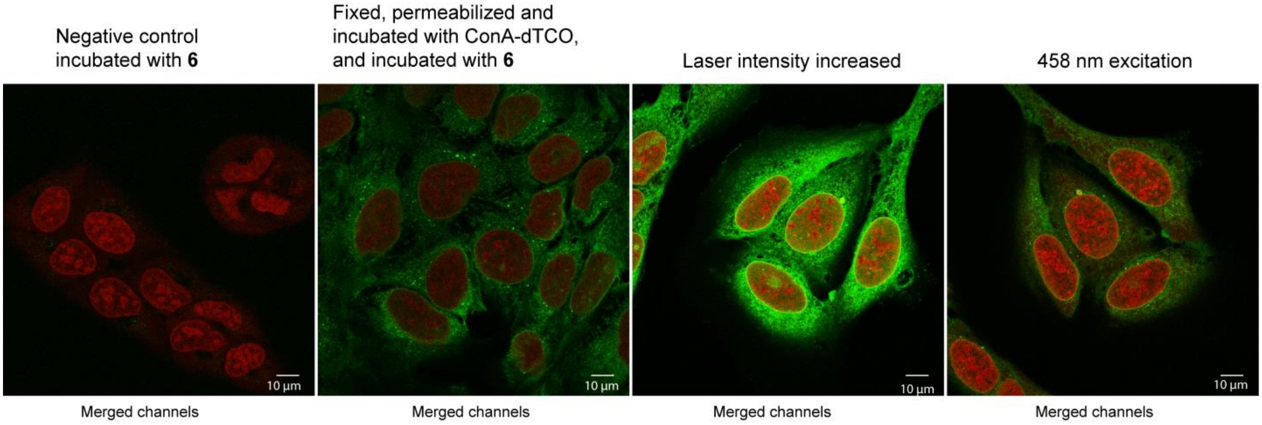

Figure S23. Fixed and permeabilized U2OS cell labeling experiment using ConA-dTCO conjugate and triazine **6**.

References

[1] M. Royzen, G. P. Yap, J. M. Fox, *J. Am. Chem. Soc.* **2008**, *130*, 3760-3761.

[2] A. Darko, S. Wallace, O. Dmitrenko, M. M. Machovina, R. A. Mehl, J. W. Chin, J. M. Fox, *Chem. Sci.* **2014**, *5*, 3770-3776.

[3] M. T. Taylor, M. L. Blackman, O. Dmitrenko, J. M. Fox, *J. Am. Chem. Soc.* **2011**, *133*, 9646-9649.

[4] *according to Patent: WO2004/85408 A1, 2004*.

[5] *according to Patent: WO2006/033551 A1, 2006*.

[6] V. N. Kozhevnikov, O. V. Shabunina, D. S. Kopchuk, M. M. Ustinova, B. König, D. N. Kozhevnikov, *Tetrahedron* **2008**, *64*, 8963-8973.

[7] N. T. Coogan, M. A. Chimes, J. Raftery, P. Mocilac, M. A. Denecke, *J. Org. Chem.* **2015**, *80*, 8684-8693.

[8] J. Cabal, F. Hampl, F. Liska, J. Patocka, F. Riedl, K. Sevcikova, *Collect. Czech. Chem. Commun.* **1998**, *63*, 1021-1030.

[9] *according to Patent: US2011/212967 A1, 2011*.

[10] D. N. Kamber, Y. Liang, R. J. Blizzard, F. Liu, R. A. Mehl, K. N. Houk, J. A. Prescher, *J. Am. Chem. Soc.* **2015**, *137*, 8388-8391.

[11] P. C. Conrad, P. L. Kwiatkowski, P. L. Fuchs, *J. Org. Chem.* **1987**, *52*, 586-591.

[12] W. Wang, S. Hong, A. Tran, H. Jiang, R. Triano, Y. Liu, X. Chen, P. Wu, *Chem. Asian J.* **2011**, *6*, 2796-2802.

[13] D. F. Eaton, *J. Photochem. Photobiol. B* **1988**, *2*, 523-531.

[14] J. Schindelin, I. Arganda-Carreras, E. Frise, V. Kaynig, M. Longair, T. Pietzsch, S. Preibisch, C. Rueden, S. Saalfeld, B. Schmid, J. Y. Tinevez, D. J. White, V. Hartenstein, K. Eliceiri, P. Tomancak, A. Cardona, *Nat. Methods* **2012**, *9*, 676-682.

Copies of NMR spectra

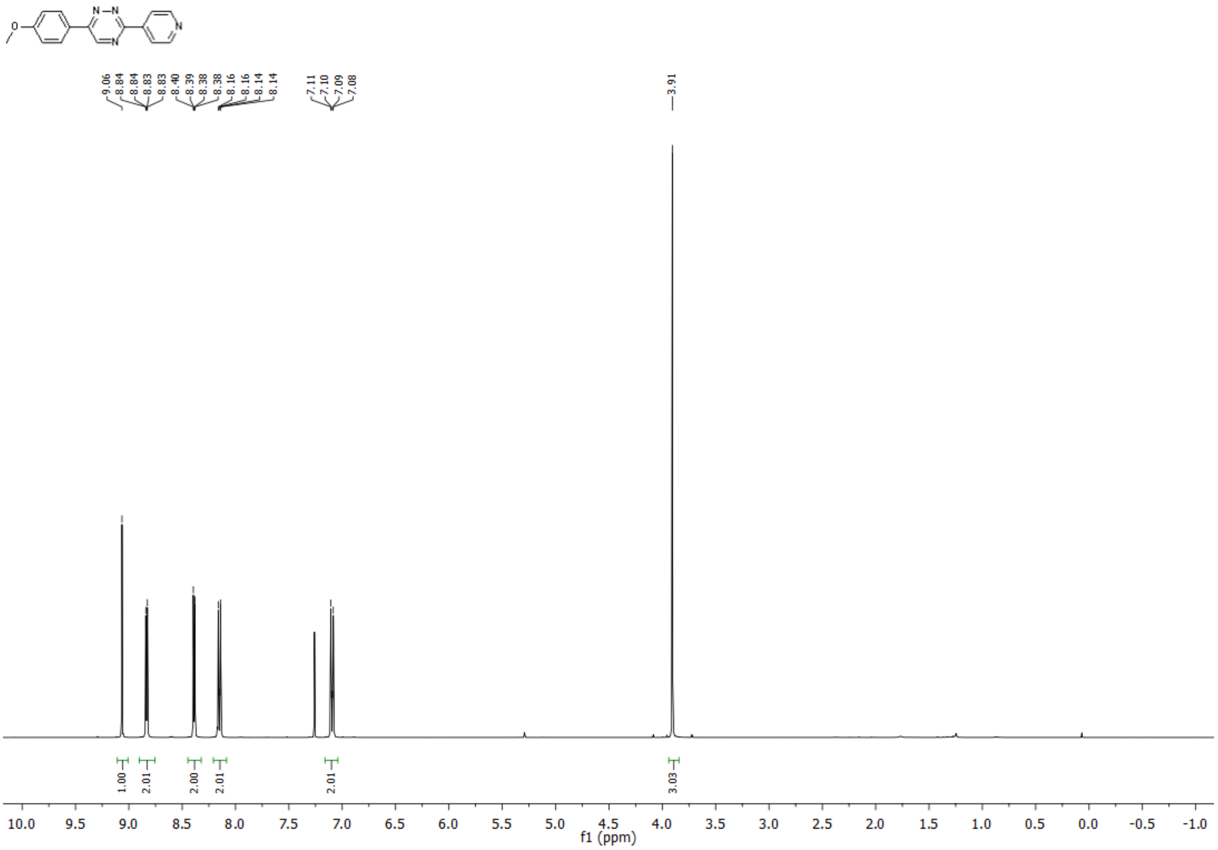

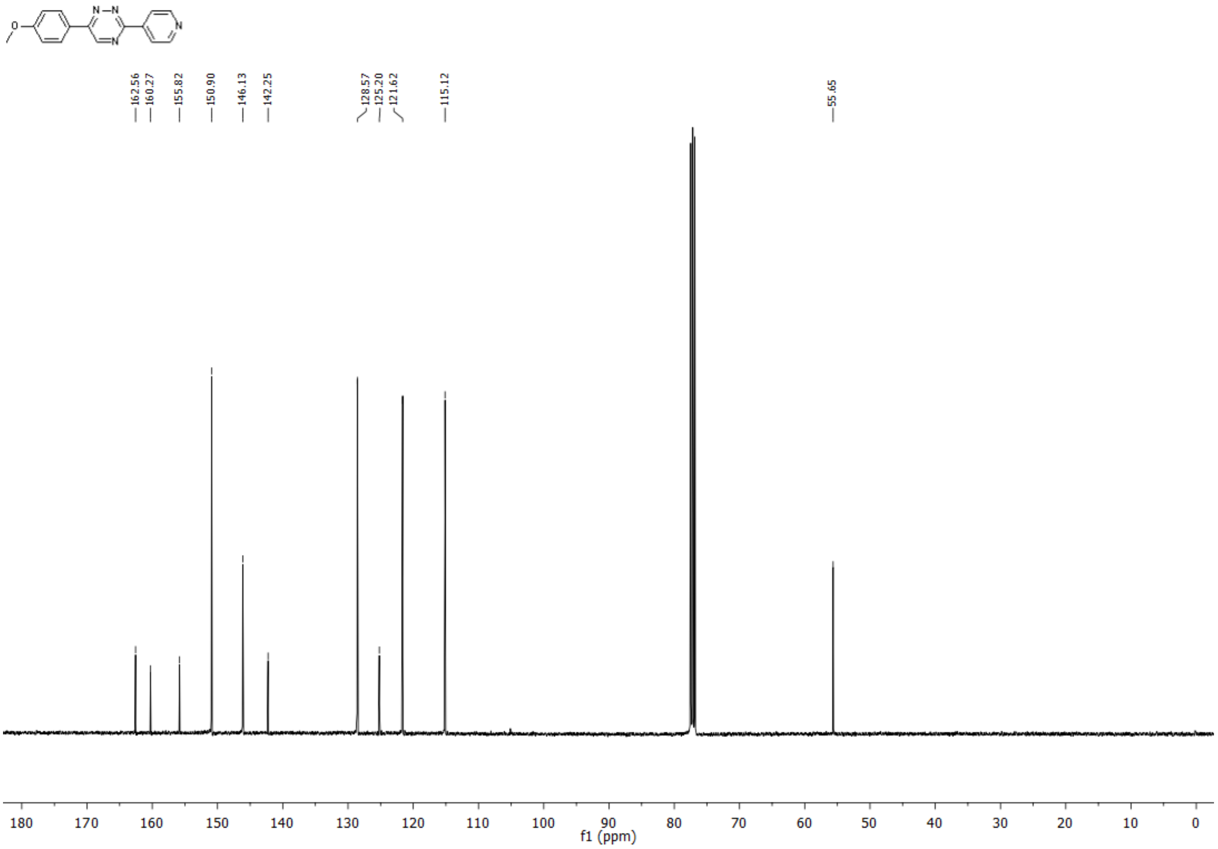

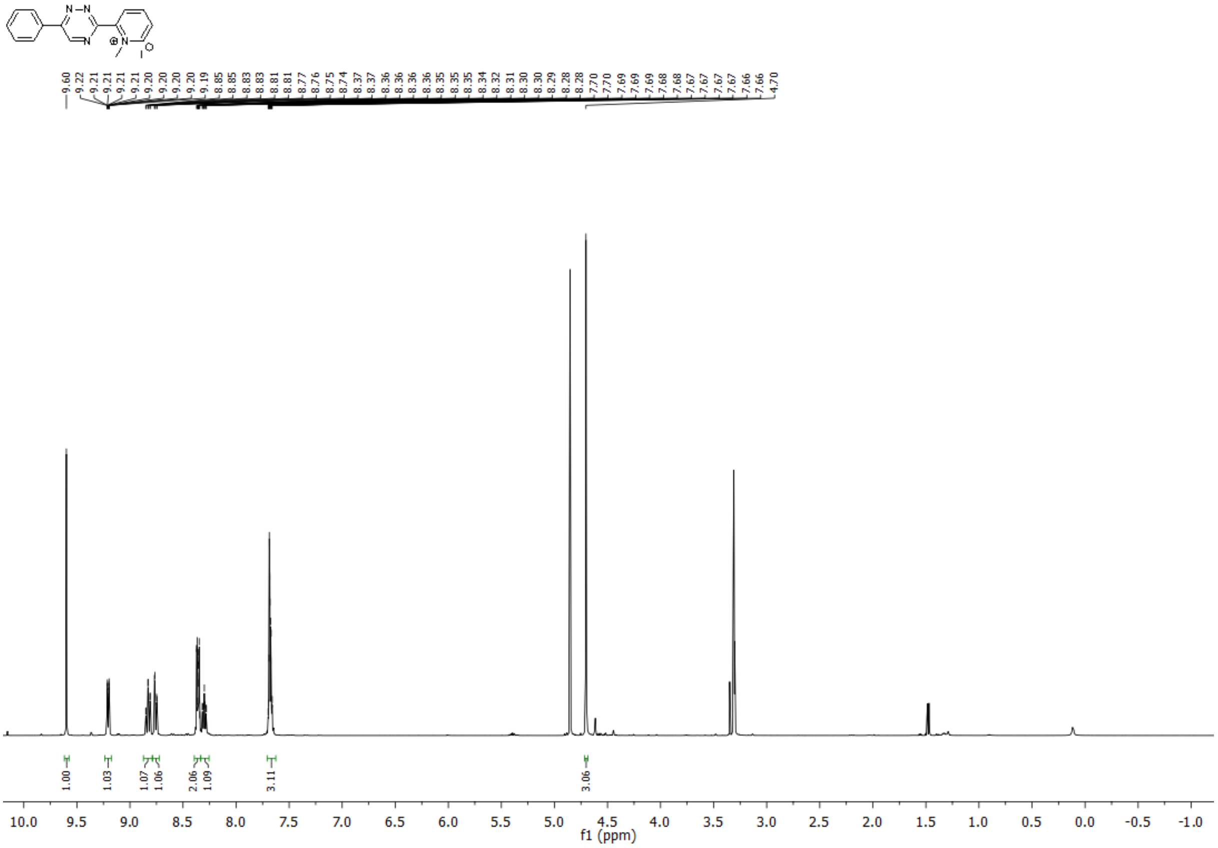

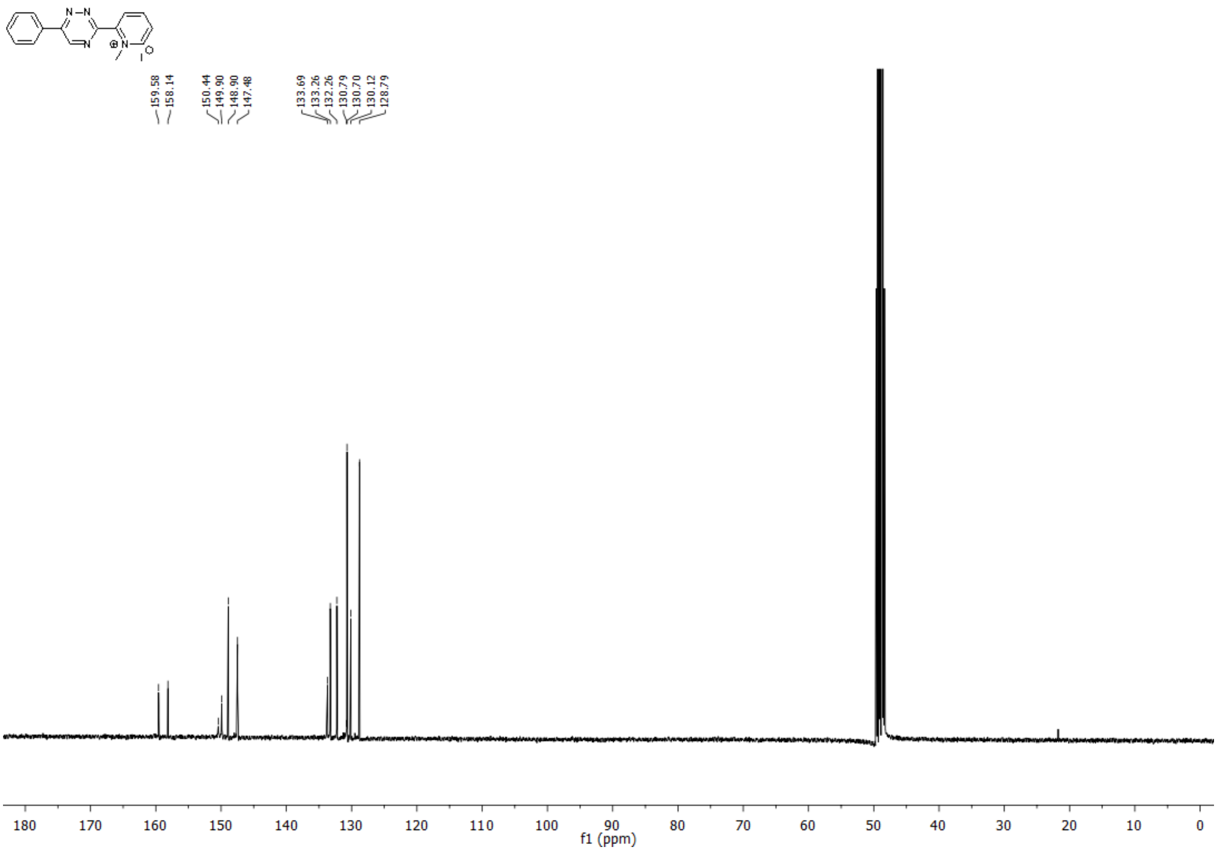

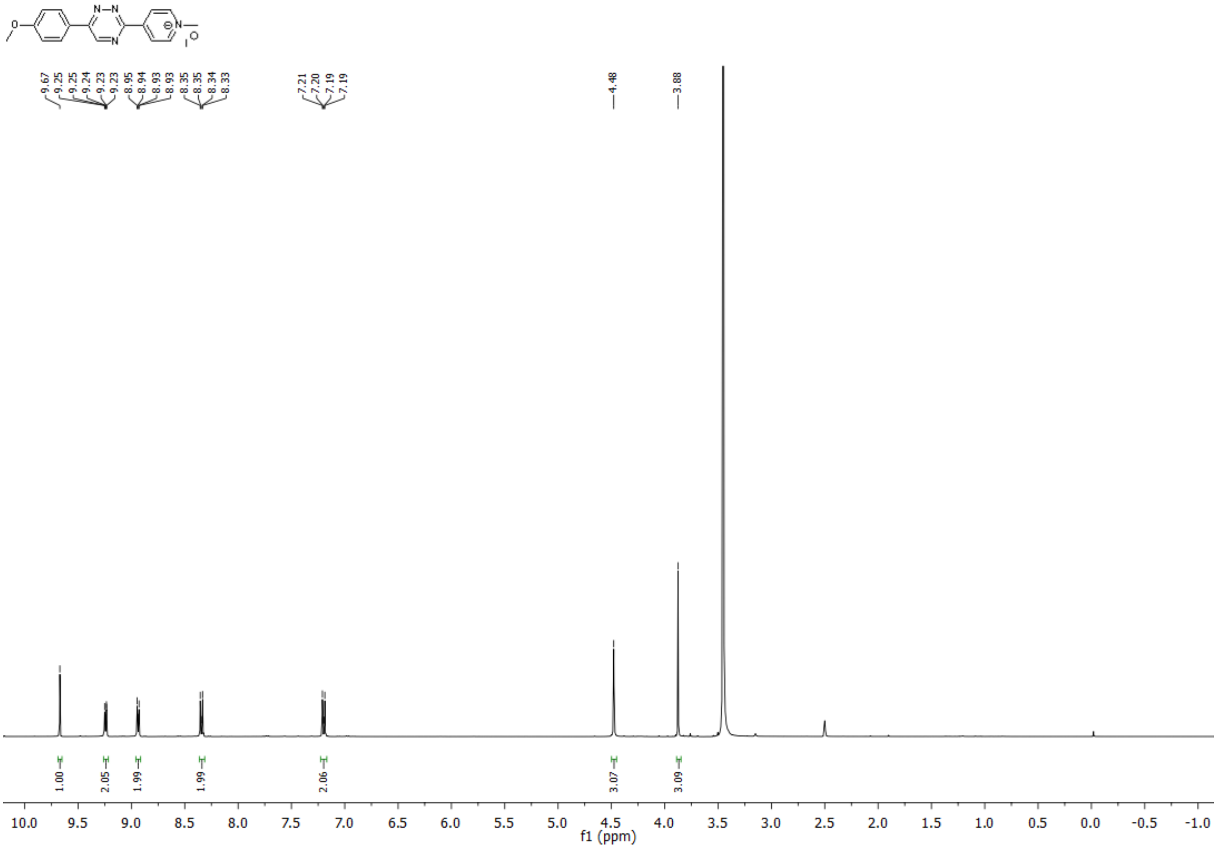
9.67
9.25
9.25
9.24
9.23
9.23
8.95
8.94
8.93
8.93
8.35
8.35
8.34
8.33
7.21
7.20
7.19
7.19
4.48
3.88
1.00
2.05
1.99
1.99
2.06
3.07
3.09
f1 (ppm)

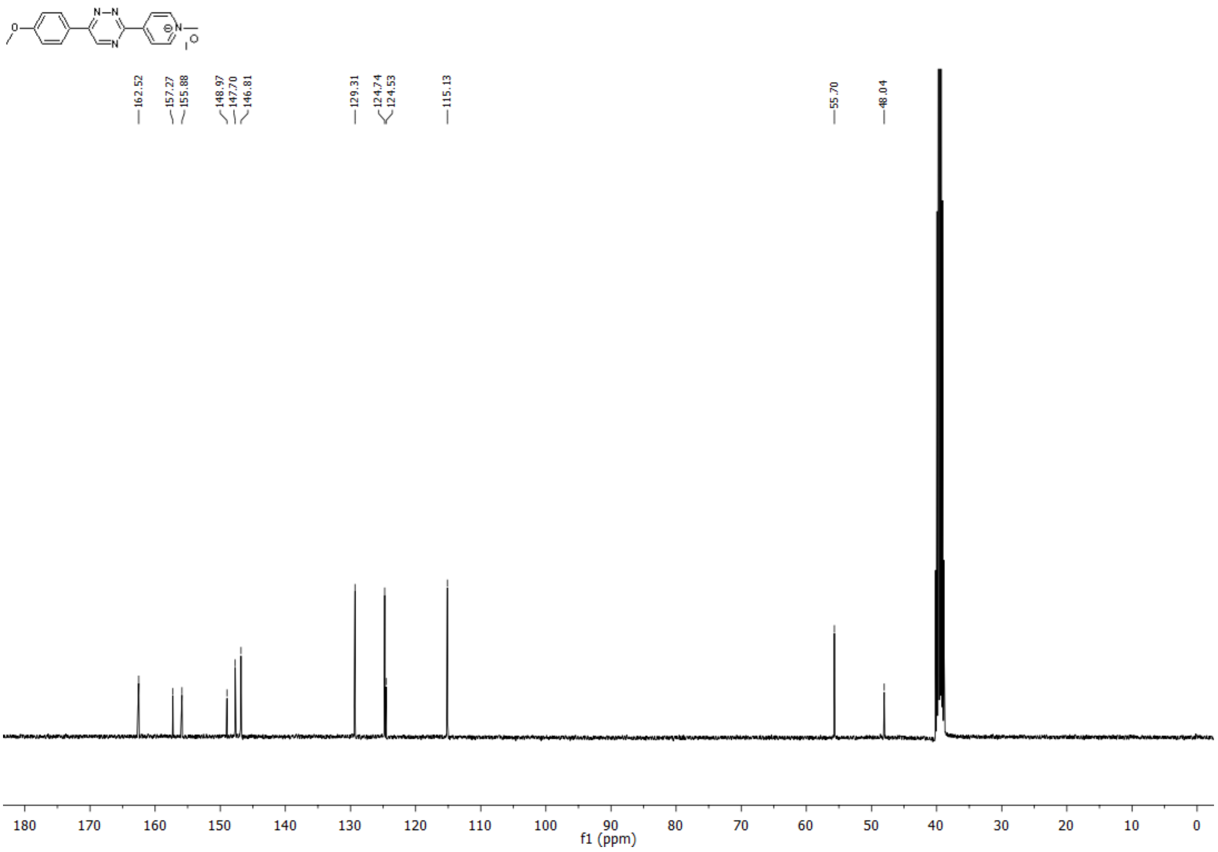
162.52
157.27
155.88
148.97
147.70
146.81
129.31
124.74
124.53
115.13
55.70
48.04
f1 (ppm)

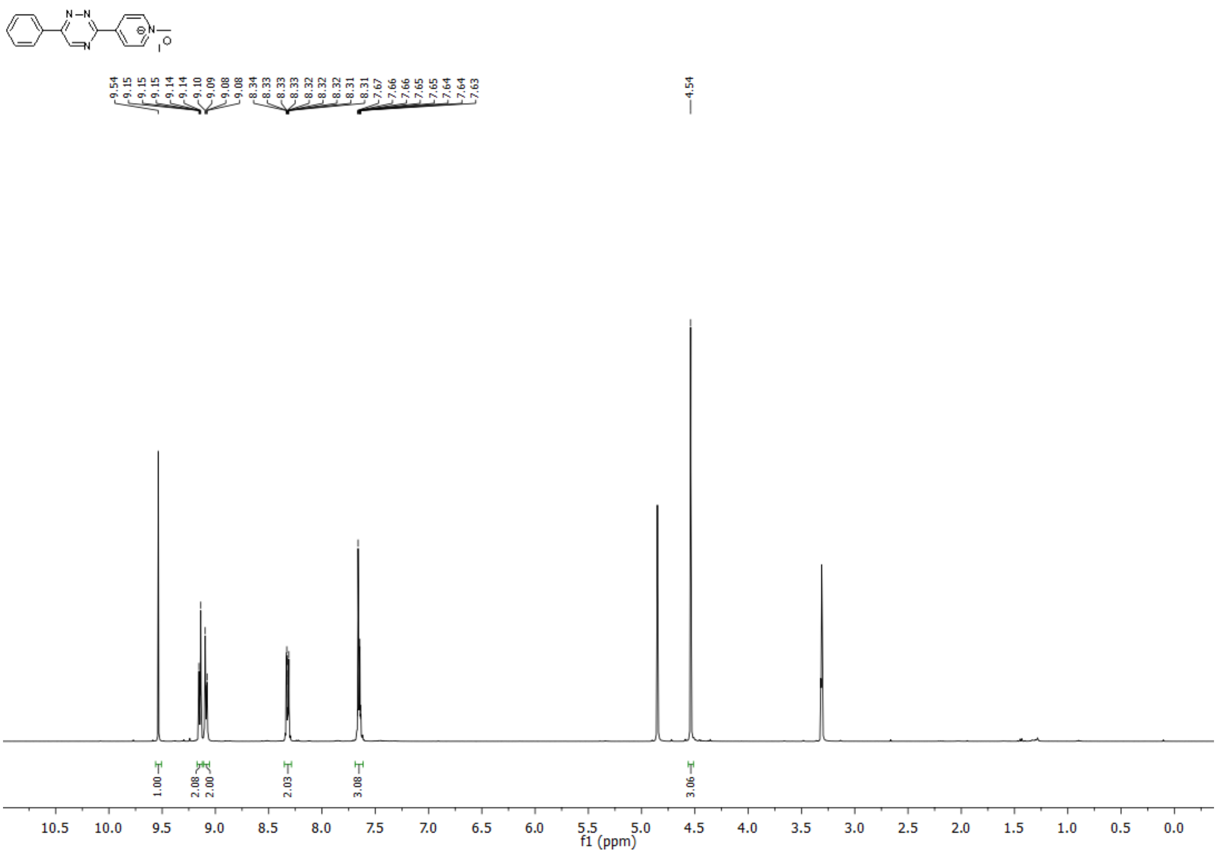

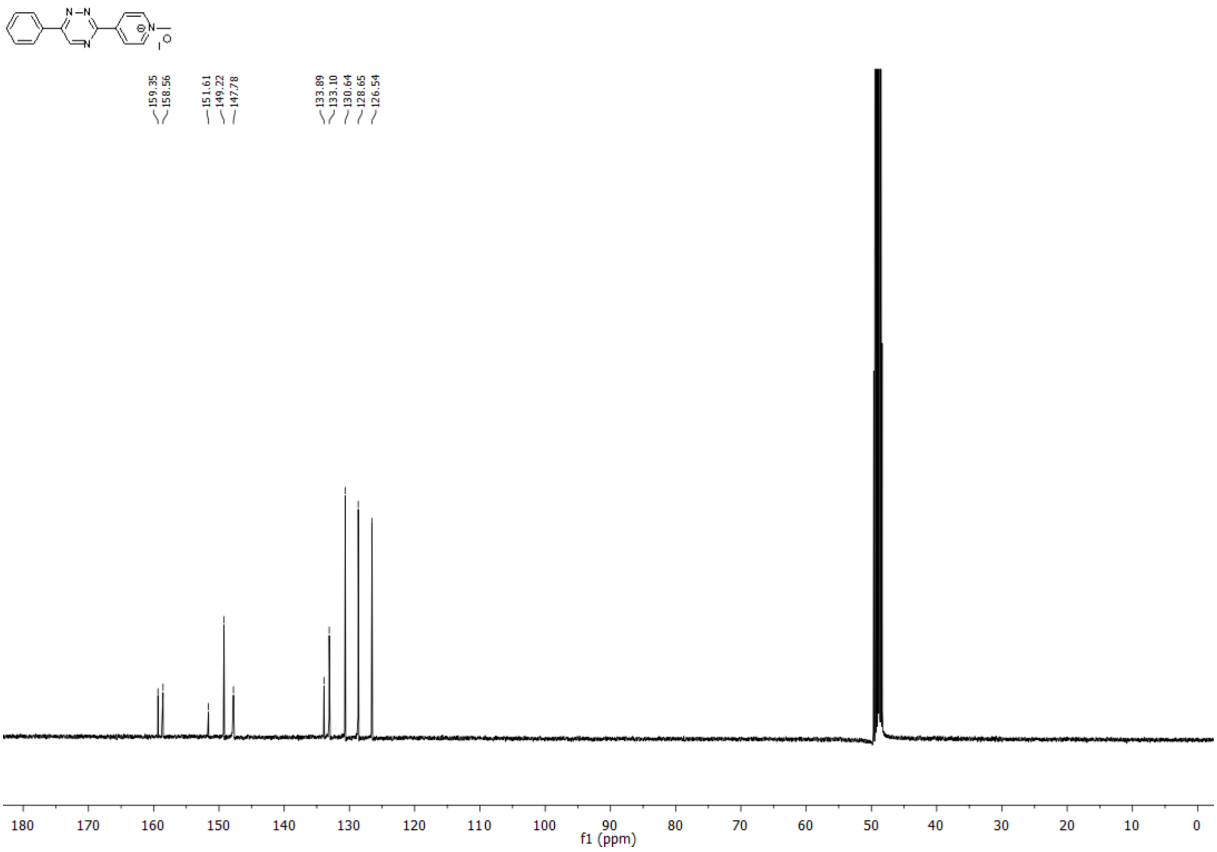

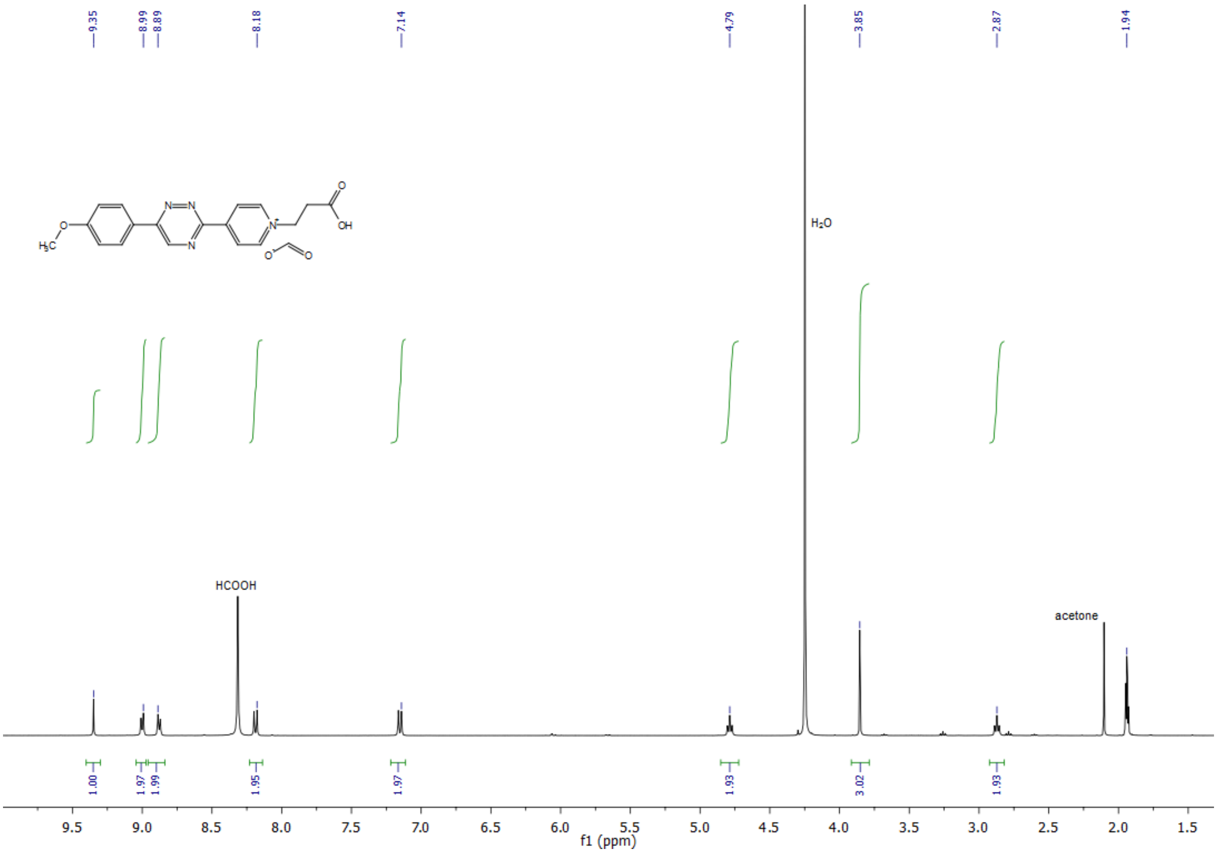
9.35
8.99
8.89
8.18
7.14
4.79
3.85
2.87
1.94
H₃C
O
N
N
N
N
N+
O
OH
O-
O
H₂O
HCOOH
acetone
1.00
1.97
1.99
1.95
1.97
1.93
3.02
1.93
9.5
9.0
8.5
8.0
7.5
7.0
6.5
6.0
5.5
5.0
4.5
4.0
3.5
3.0
2.5
2.0
1.5
f1 (ppm)

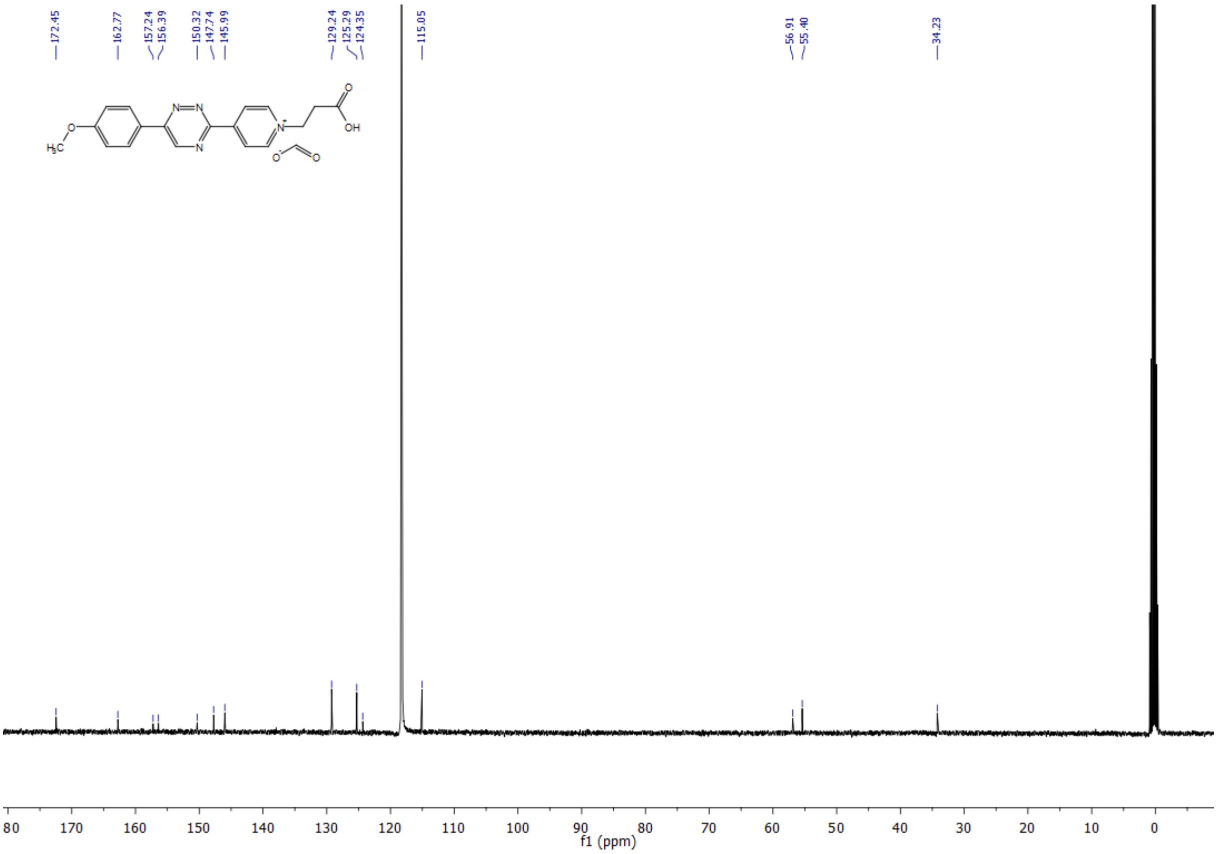
172.45
162.77
157.24
156.39
150.32
147.74
145.99
129.24
125.29
124.35
115.05
56.91
55.40
34.23
H₃C
O
N
N
N
N+
O
OH
O-
O
80
170
160
150
140
130
120
110
100
90
80
70
60
50
40
30
20
10
0
f1 (ppm)

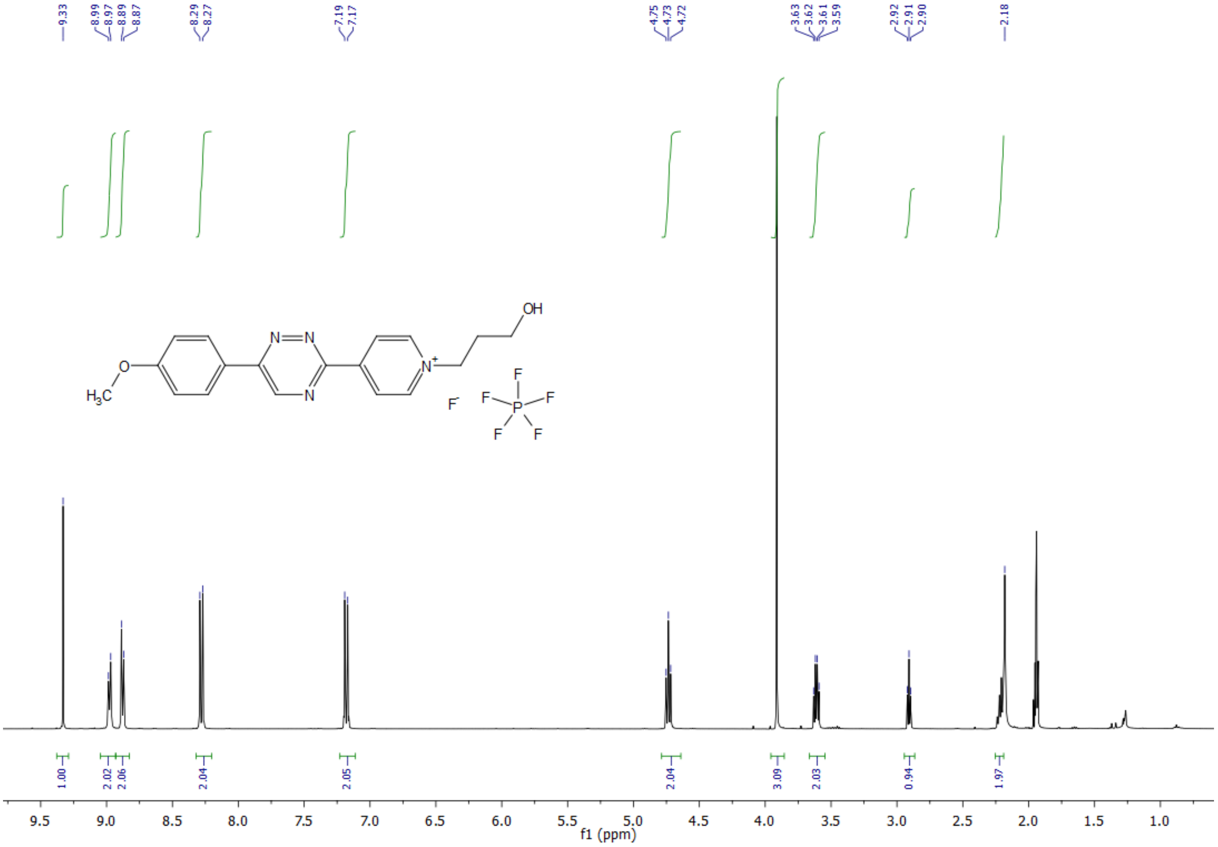
9.33
8.99
8.97
8.89
8.87
8.29
8.27
7.19
7.17
4.75
4.73
4.72
3.63
3.62
3.61
3.59
2.92
2.91
2.90
2.18
OH
N=N
N
N^+
O
H_3C
F^-
F
P
1.00
2.02
2.06
2.04
2.05
2.04
3.09
2.03
0.94
1.97
f1 (ppm)

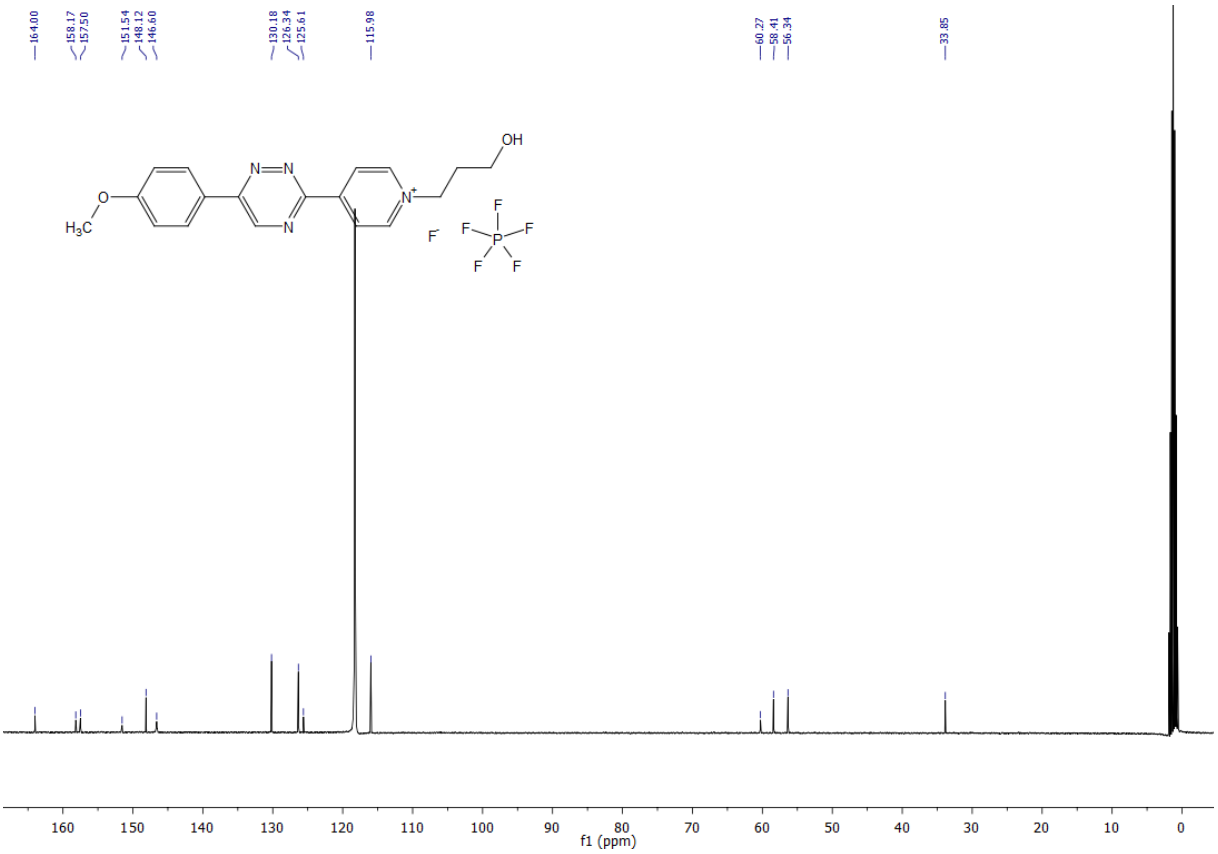
164.00
158.17
157.50
151.54
148.12
146.60
130.18
126.34
125.61
115.98
60.27
58.41
56.34
33.85
OH
N=N
N
N^+
O
H_3C
F^-
F
P
f1 (ppm)

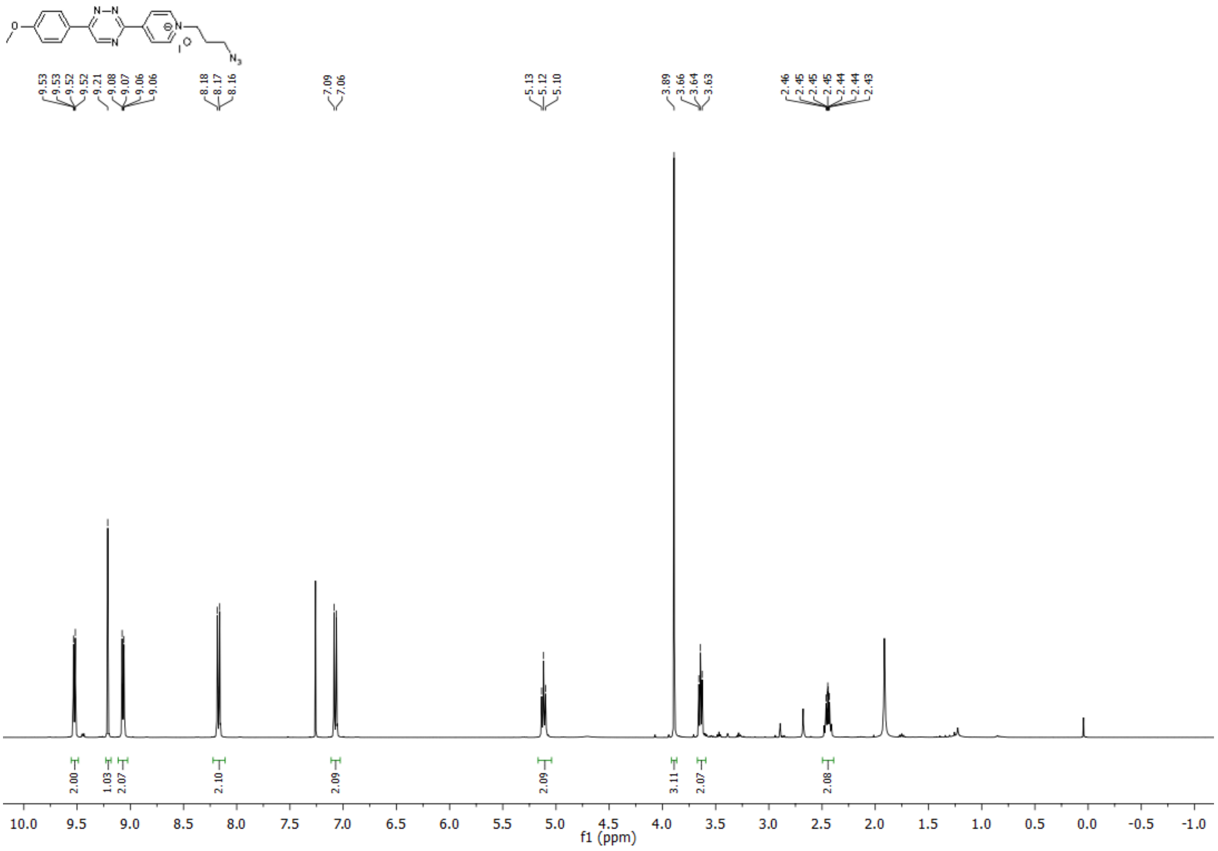
9.53 9.53 9.52 9.52 9.21 9.08 9.07 9.06 9.06
8.18 8.17 8.16
7.09 7.06
5.13 5.12 5.10
3.89 3.66 3.64 3.63
2.46 2.45 2.45 2.45 2.44 2.44 2.43
2.00 1.03 2.07 2.10 2.09 2.09 3.11 2.07 2.08
f1 (ppm)

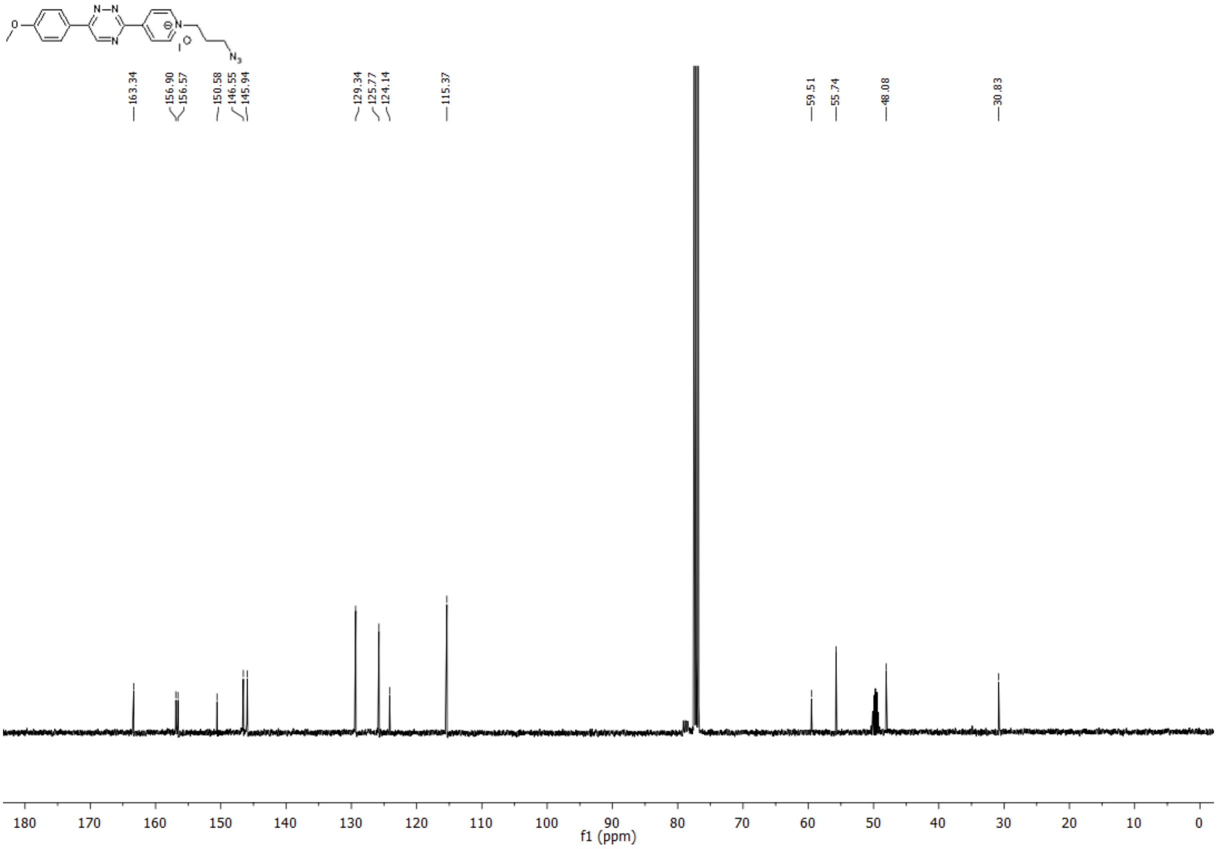
163.34
156.90 156.57
150.58 146.55 145.94
129.34 125.77 124.14
115.37
59.51
55.74
48.08
30.83
f1 (ppm)

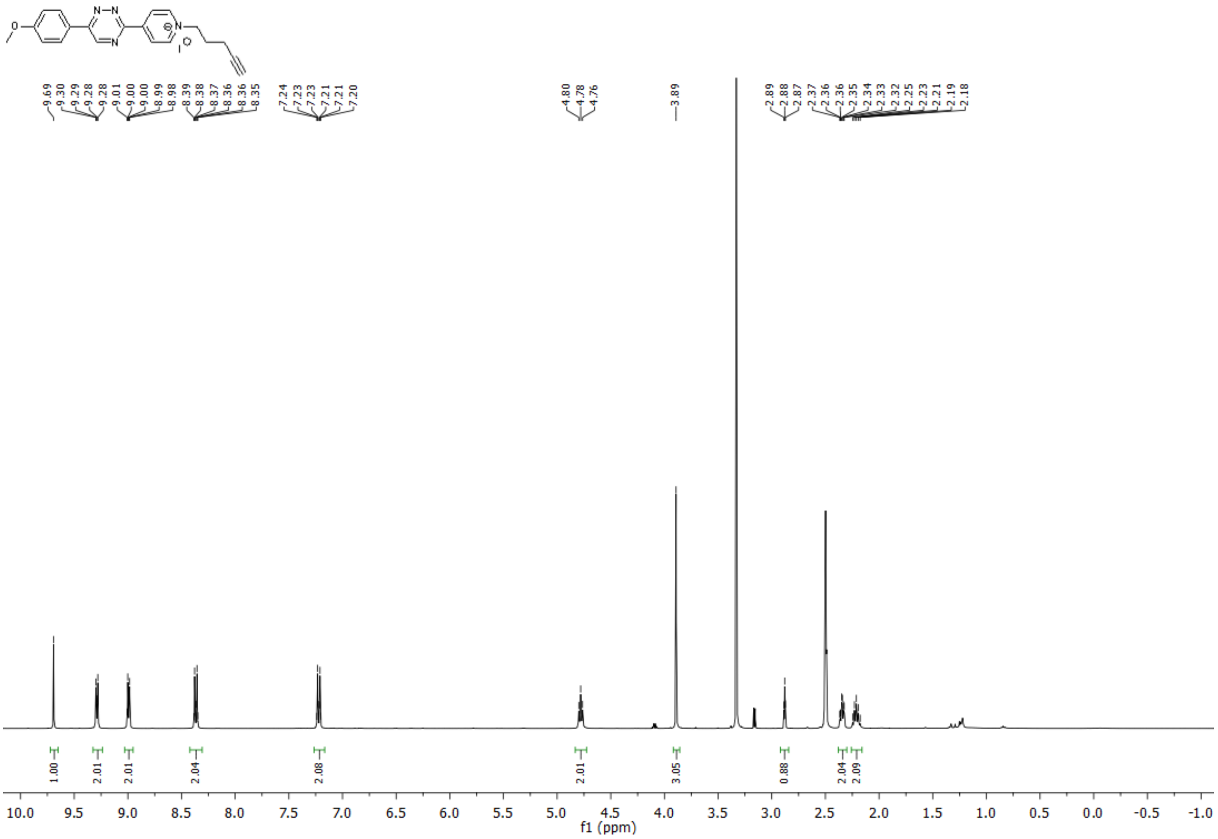
9.69
9.30
9.29
9.28
9.28
9.01
9.00
9.00
8.99
8.98
8.39
8.38
8.37
8.36
8.36
8.35
7.24
7.23
7.23
7.21
7.21
7.20
4.80
4.78
4.76
3.89
2.89
2.88
2.87
2.37
2.36
2.36
2.35
2.34
2.33
2.32
2.25
2.23
2.21
2.19
2.18
1.00
2.01
2.01
2.04
2.08
2.01
3.05
0.88
2.04
2.09
f1 (ppm)

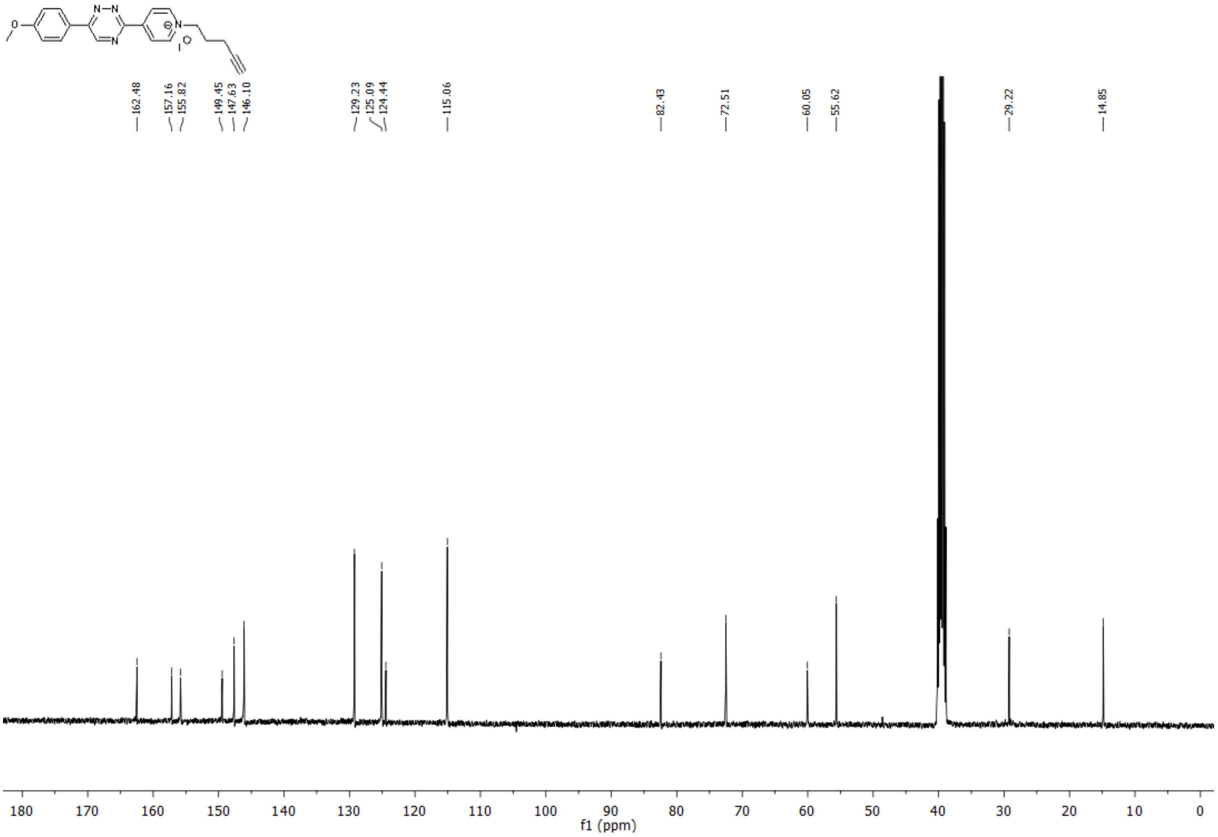
162.48
157.16
155.82
149.45
147.63
146.10
129.23
125.09
124.44
115.06
82.43
72.51
60.05
55.62
29.22
14.85
f1 (ppm)
